# Tumor Biology Hides Novel Therapeutic Approaches to Diffuse Large B-Cell Lymphoma: A Narrative Review

**DOI:** 10.3390/ijms252111384

**Published:** 2024-10-23

**Authors:** Romana Masnikosa, Zorica Cvetković, David Pirić

**Affiliations:** 1 Department of Physical Chemistry, Vinca Institute of Nuclear Sciences—National Institute of the Republic of Serbia, University of Belgrade, Mike Petrovica Alasa 12-14, 11000 Belgrade, Serbia; david.piric@vin.bg.ac.rs; 2 Department of Hematology, Clinical Hospital Centre Zemun, Vukova 9, 11000 Belgrade, Serbia; 3 Faculty of Medicine, University of Belgrade, Dr Subotića 8, 11000 Belgrade, Serbia

**Keywords:** diffuse large B-cell lymphoma, B-cell NHL, molecular classifications, knowledge map analysis, tumor lipidome remodeling, microbiome, cuproptosis, ferroptosis, immunotherapy

## Abstract

Diffuse large B-cell lymphoma (DLBCL) is a malignancy of immense biological and clinical heterogeneity. Based on the transcriptomic or genomic approach, several different classification schemes have evolved over the years to subdivide DLBCL into clinically (prognostically) relevant subsets, but each leaves unclassified samples. Herein, we outline the DLBCL tumor biology behind the actual and potential drug targets and address the challenges and drawbacks coupled with their (potential) use. Therapeutic modalities are discussed, including small-molecule inhibitors, naked antibodies, antibody–drug conjugates, chimeric antigen receptors, bispecific antibodies and T-cell engagers, and immune checkpoint inhibitors. Candidate drugs explored in ongoing clinical trials are coupled with diverse toxicity issues and refractoriness to drugs. According to the literature on DLBCL, the promise for new therapeutic targets lies in epigenetic alterations, B-cell receptor and NF-κB pathways. Herein, we present putative targets hiding in lipid pathways, ferroptosis, and the gut microbiome that could be used in addition to immuno-chemotherapy to improve the general health status of DLBCL patients, thus increasing the chance of being cured. It may be time to devote more effort to exploring DLBCL metabolism to discover novel druggable targets. We also performed a bibliometric and knowledge-map analysis of the literature on DLBCL published from 2014–2023.

## 1. Introduction

Diffuse large B-cell lymphoma (DLBCL), the most common type of non-Hodgkin lymphoma (NHL) worldwide, represents around 30–40% of all cases in different geographic regions. The tumor often manifests as a quickly growing mass in a single or several nodal or extranodal sites. Regardless of the standpoint, DLBCL is heterogeneous, whether clinical features, genetic alterations, response to therapy (RTT), or prognosis. The most common type of this malignancy, representing 80–85% of all cases, is DLBCL, not otherwise specified (NOS), which is the center of our present narrative review.

**Figure 1:**
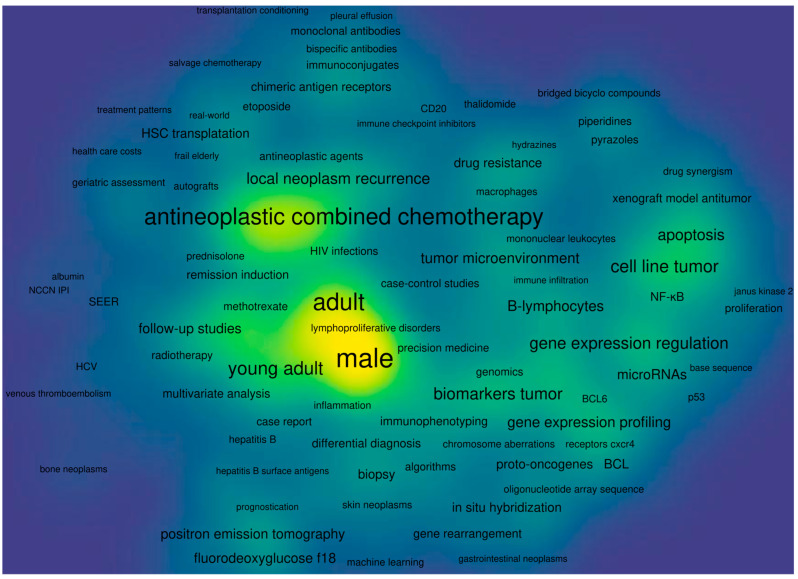
The co-occurrence density map of indexed keywords, chosen by authors who published their research and review articles on diffuse large B-cell lymphoma, according to PubMed database. The deeper the yellow color of a node, the more frequently keywords appear. The image was created in Vosviewer version 1.6.20.

The main idea behind this review is to tell a story of DLBCL that covers all critical aspects of this unimaginably heterogeneous blood malignancy. Taking into account that descriptions of clinical trials and the efficacy of anti-neoplastic combined chemotherapy have comprised the majority of the scientific literature on DLBCL (Figure 1), we present niches that have been much less explored. We first briefly describe clinical aspects of DLBCL, risk factors, disease classification according to the current standards, patient stratification, and disease classification schemes. We then outline the molecular features of DLBCL and the genetic, epigenetic, immunobiological, and metabolic aspects of the disease. A novel programmed cell death (PCD) modality, cuproptosis, and its involvement in B-cell lymphoma is also depicted. We outline another PCD form, ferroptosis, whose role in B-cell tumorigenesis has recently appeared. Finally, we depict ground research on gut microbiota’s (GMB) role in pathology and therapy of DLBCL.

The second theme covered by the present review encompasses therapeutic paradigms pertinent to B-cell NHL, particularly DLBCL. We present anti-tumor drugs that have been used for the frontline immuno-chemotherapy of B-cell malignancies, agents recently approved for treating relapsed or refractory (R/R) (D)LBCL, and investigational drugs evaluated in the recent or ongoing clinical trials. We explain the modus operandi of the above-mentioned therapeutics from the biochemical point of view, i.e., without plunging into clinical details of therapeutic regimens, applied dosages, safety and resistance-to-therapy issues, data on clinical outcomes, and so on. Moreover, we discuss the potential targets arising from aberrant genomic and epigenomic landscapes, altered immune cell signaling pathways, tumor-induced metabolic reprogramming, PCD mechanisms, and changed GMB composition in B-cell lymphoma and DLBCL, particularly those evaluated in pre-clinical settings.

Despite the progress in our biological understanding of DLBCL, we have yet to translate that into improved frontline therapy. The research community dealing with hematological malignancies (from now on, just community), and particularly DLBCL, has long been aware that fully deciphered intracellular and extracellular mechanisms involved in DLBCL pathogenesis, to a molecular level, and a deep understanding of all aspects of the DLBCL biology are absolute necessities if we want to design more effective drugs for treating this malignancy. Therefore, the immense molecular heterogeneity of DLBCL and the lack of deep knowledge of DLBCL biology are the main hurdles that block meaningful improvements of anti-lymphoma therapy.

The therapies in use to cure DLBCL encompass chemotherapy and targeted therapy, including immunotherapy, and cell adoption therapy. These therapeutic modalities have been comprehensively covered in an overwhelming number of recent reviews to the point that it is almost impossible to read them all. Therefore, our third goal was to perform a bibliometric and knowledge-map analysis of the published literature on DLBCL in order to extract the research hotspots in this field. We intended to write a “review of reviews”, aiming to reach a broad target audience, from newcomers to the field and busy clinicians, who lack the time to read the overwhelming amount of data being published on DLBCL by the day, to analytical biochemists and life scientists who perform diverse omics studies on tissue and plasma samples obtained from DLBCL patients in an effort to figure out the pathophysiological and biochemical mechanisms of the disease.

## 2. Current Classification Schemes of Large B-Cell Lymphomas

The tenth most common malignancy worldwide, NHL, is the most common hematological malignancy, which, according to Global Cancer Statistics, comprises 2.8% of all de novo (dn) cancer cases and 2.6% of all cancer deaths [1]. NHLs are malignancies of the elderly (30% of patients are over 75) and are more prevalent in males than in females. In Western countries, B-cell NHLs represent 85% of all NHLs and originate from various functionally distinct B-cell subsets, yielding over 70 currently recognized subtypes. DLBCL is the most common neoplasm of mature B-cells, accounting for 30–40% of NHLs [2]. DLBCL incidence is higher in males than females, which may be due to estrogen [3]. Moreover, being a female is a favorable prognostic factor [4,5].

Morphologically, DLBCL manifests as diffuse growth patterns of medium- to large-sized B-cells (e.g., centroblasts, immunoblasts) and blastoid (anaplastic) variants. Ever since the first purely morphologically oriented classifications of lymphomas, which are those of Gall and Mallory (1942) [6] and Rappaport (1966) [7], the examination of NHL cytology remains an unavoidable part of its classification [8]. The latest 5th edition of the World Health Organization (WHO) classification of hematolymphoid tumors (WHO-HAEM5, 2022) recognizes three morphological subtypes: centroblastic, immunoblastic, and anaplastic [9,10]. For cases where the diffuse growth pattern cannot be recognized, WHO-HAEM5 introduces the term “large B-cell lymphoma” (LBCL) [9,11]. Besides WHO-HAEM5, another system for classifying hematological malignancies, the International Consensus Classification (ICC), was proposed in 2022 [12]. Both coexisting classifications are based on pathological (cytology, histology, and tissue architecture), clinical, immunological, cytogenetic, and molecular features, resulting in an upgrade of previous provisional entities to definite entities, but WHO-HAEM5, unlike ICC, does not consider provisional categories anymore [9,11,12,13]. Regarding LBCL, WHO-HAEM5 features several major changes compared to the previous update of the 4th of edition of the WHO classification of malignant lymphomas (WHO-HAEM4R) [14,15].

Although LBCL may arise as the transformation of indolent lymphomas such as chronic lymphocytic leukemia/small lymphocytic lymphomas (CLL/SLL), marginal zone lymphoma (MZL), and follicular lymphoma (FL), or from nodular lymphocyte predominant Hodgkin lymphoma (HL), a large majority appear as dnLBCLs. WHO-HAEM5, for the first time, includes these as a separate entity. LBCLs under WHO-HAEM5 encompass 18 entities and include a few upgrades of previous provisional entities, such as high-grade B-cell lymphoma (HGBCL) with *11q* aberrations and LBCL with *IRF4* rearrangement. WHO-HAEM5 includes several previously unrecognized entities, such as fluid overload-associated LBCL, primary LBCL of immune-privileged sites (i.e., central nervous system, testis, and vitreoretina), fibrin-associated LBCL, and mediastinal gray zone lymphoma (Table 1). However, it still keeps most previously defined entities: DLBCL, NOS, T-cell/histiocyte-rich LBCL, primary cutaneous DLBCL-leg type, intravascular LBCL, Epstein–Barr virus-positive (EBV+) DLBCL, DLBCL associated with chronic inflammation, ALK-positive LBCL, plasmablastic lymphoma, primary mediastinal B-cell lymphoma, and HGBCL. Most DLBCL, NOS are of primary nodal origin and lack the criteria to be classified as one of the more specific entities. They most commonly involve lymph nodes but may also arise in extra-nodal lymphatic tissues (e.g., tonsils), Peyer patches of the gastrointestinal tract, or extra-lymphatic tissues throughout the body [10]. The concept of the subdivision of DLBCL, NOS, based on cell-of-origin (COO), into two molecular subtypes—germinal center B-cell-like (GCB-DLBCL) and activated B-cell-like (ABC-DLBCL)—has been retained in WHO-HAEM5 [9] and ICC [12]. Table 1 summarizes the taxonomic similarities and differences between WHO-HAEM5, WHO-HAEM4R, and ICC, along with the key pathological and clinical features of LBCL and DLBCL.

### Diffuse Large B-Cell Lymphoma, Not Otherwise Specified

Gene expression profiling (GEP) assays, e.g., Lymph2Cx GEP, have yet to be widely utilized in clinical practice, despite being the more accurate way to determine the DLBCL COO subtype. Instead, several algorithms based on immunohistochemistry (IHC) have been developed, such as the Hans algorithm, which relies on the staining of three proteins: CD10, BCL6, and MUM1 [16]. Patients assigned as non-GCB/ABC were considered to have a worse prognosis compared to patients with GCB-DLBCL [17]. A subgroup of DLBCL patients within the GCB-DLBCL subtype with dual *MYC* and *BCL2* rearrangements, representing 5% to 10% of cases, has the worst prognosis [18]. WHO-HAEM5 separates DLBCL/HGBCL with *MYC* and *BCL2* rearrangements from the previous entity—HGBCL with *MYC* and *BCL2* and/or *BCL6* rearrangements, also known as “double-hit”/“triple-hit” (DH/TH) lymphomas [9]. Meanwhile, ICC retains HGBCL with *MYC* and *BCL6* rearrangements as a provisional entity alongside the new entity, HGBCL with *MYC* and *BCL2* rearrangements [12]. The newly introduced categories underscore the importance of more accurately defined DLBCL entities and highlight their clinical distinction. Since DLBCL is a more general term than DLBCL, NOS, and also encompasses HGBCL, we shall use the term DLBCL whenever the cited articles did not specify DLBCL, NOS. It is worth noting that double-expressor lymphomas that overexpress MYC and BCL2 proteins are aggressive DLBCLs and are associated with a dismal prognosis [19]. DLBCL, NOS is a diagnosis of exclusion with an elusive and multifaceted etiology, and several factors contribute to the pathogenesis, particularly immune dysregulation as in B-cell-activating autoimmune diseases, family history of NHL, and viral, environmental, and occupational exposures [2,9,12,20,21].

Less than 20% of EBV+ DLBCL cases can be assigned to COO subtypes, pointing out the unique biology of this entity, which represents a rare, aggressive subtype with an adverse clinical outcome [22]. EBV is also related to DLBCL associated with chronic inflammation and fibrin-associated LBCL [23,24]. A higher incidence of DLBCL is also found in individuals affected by other oncogenic DNA viruses such as hepatitis B virus (HBV), human papillomavirus (HPV), and Simian virus 40, as well as RNA viruses: human immunodeficiency virus (HIV) and hepatitis C virus (HCV) [25]. The risk of developing DLBCL is 98 times higher in people living with HIV (PWH) than in the general population. The newest anti-retroviral therapy decreased the DLBCL incidence rate among PWH from 63% to 35% [26]. Timely vaccination against HBV and HPV, and HCV eradication will presumably contribute to a further decrease in DLBCL incidence. The potential roles of pathogens and chronic immune stimulation in developing DLBCL have been recently reviewed [27].

How many people die of DLBCL? The annual incidence in the USA has been around 7 cases per 100,000 persons [1] and is projected to increase by 4% by 2025 [28]. The 5-year relative survival rate has improved due to fewer PWH also having DLBCL and increased usage of intensive chemotherapy regimens and improved supportive care [29]. However, despite all of these achievements, we are still left with 40% of patients who will experience primary refractory disease or eventually relapse (R/R DLBCL), with only 50% of them qualifying for autologous hematopoietic stem cell transplantation, A(H)SCT (or auto-HCT) [28]. Specific mortality in patients with DLBCL has been recently assessed using data on 24,402 patients from the Surveillance, Epidemiology, and End Results database. The risk model was built on 13 variables identified as independent risk factors for cause-specific mortality, such as race, tumor site (extra-nodal or nodal), Ann Arbor stage, surgery, radiation therapy, chemotherapy, sequence of systemic therapy and surgery, treatment timing, B symptoms, whether it was the first primary tumor, age, marital status, and median annual household income. The area under the receiver operating characteristic (ROC) curve (AUC) for predicting 6-month, 1-year, and 3-year DLBCL-specific mortality was 0.747, 0.721, and 0.697, respectively. The most significant predictor of DLBCL-specific mortality was patients’ age, followed by the Ann Arbor stage and the administration of chemotherapy [30].

DLBCL staging and clinical response assessment should be performed using Ann Arbor staging and the Lugano classification criteria [31,32,33]. Highly sensitive 18F-fluorodeoxyglucose positron-emission tomography with computed tomography (PET-CT) has replaced CT [31]. Large B-cells in bone marrow biopsy are associated with a poor prognosis. However, bone marrow biopsy is becoming redundant for patients undergoing PET-CT staging [2]. For the patients who remain event-free for two years from the diagnosis, expected overall survival (OS) is comparable to OS in the general, age-matched population [34].

Even in the current molecular era, consensus within the research community on the optimal molecular technique for risk stratification of DLBCL patients is still missing. Three scoring systems, based on simple clinical parameters—age, lactate dehydrogenase (LDH) levels, number/sites of involvement, stage, and Eastern Cooperative Oncology Group performance status—have been in widespread use: the International Prognostic Index (IPI), revised IPI (R-IPI), and National Comprehensive Cancer Network IPI (NCCN-IPI). In a multicenter study on 2124 DLBCL patients treated with frontline rituximab (RTX) plus cyclophosphamide, doxorubicin, vincristine, and prednisone (R-CHOP) or its variant, NCCN-IPI outperformed IPI and R-IPI in predicting 5-year OS [35]. A prognostic model for R-CHOP-treated DLBCL patients requires an update, i.e., integrating biomarkers to improve discrimination to the acceptable level of, e.g., c-index 0.7. For example, *MYC* rearrangements (*MYC*-R) negatively affect the survival of DLBCL patients with Ann Arbor stage III/IV, but in stage I, the OS is excellent irrespective of the *MYC*-R status [36]. Recent efforts to stratify patients have emerged in the literature. A gene signature extracted from two independent DLBCL cohorts can predict the response to R-CHOP. *EBF1*, *MYO6*, and *CALR* expression levels were associated with a significantly worse OS [37]. In another study, a prognostic mutation signature was evaluated using 94 progression-free survival (PFS)-related genes incorporated in a prognostic index for long-term survival in DLBCL patients. The index accurately predicted the 12-, 36-, and 60-month PFS with AUC well over 95% [38].

Despite the immense molecular heterogeneity of DLBCL, no biomarkers predictive of R-CHOP response have been validated. The potential of molecular biomarkers in predicting the prognosis of DLBCL has been discussed in a review, in which the authors suggested that single nucleotide polymorphisms (SNPs) of genes that encode for proteins involved in the transport, actions, metabolism, and removal of anti-neoplastic drugs, as well as oxidative stress, apoptosis, DNA repair, immunity, and angiogenesis, may stand behind drug efficacy or toxicity. Two SNPs within genes involved in the transcriptional regulation and cell cycle progression were identified to be associated with the event-free survival and OS of R-CHOP-treated DLBCL cases by the genome-wide association study (GWAS) [39]. We are waiting for more studies to unveil novel genomic loci and SNPs associated with RTT by other-than-R-CHOP regimens, like the study of Rosenberger [40].

## 3. Diffuse Large B-Cell Lymphoma Biology, B-Cell Receptor

The defining features of B-cells, central players of the adaptive immunity responsible for long-lasting immunity after infection, are their ability to produce antibodies (Abs) and secrete cytokines to recruit other immune cells to the spot where Abs are bound to antigens. Abs are immunoglobulins (IgM, IgG, IgA, IgE, and IgD) that can be inserted in the plasma membrane of B-cells, where they are part of a multiprotein B-cell receptor (BCR) complex, or B-cells secrete them into extracellular space. B-cells may also play a role as antigen-presenting cells (APCs) when inactivated [41].

When a BCR recognizes a matching antigen, the B-cell that carries that BCR becomes “activated”. Each B-cell is equipped with a unique BCR, and the entirety of BCRs throughout our body is known as the “BCR repertoire” [42]. The specificity of B-cell responses relies on the uniqueness of the amino acid sequence in the antigen-binding domain of Ig, which is the consequence of a naturally smart phenomenon. Functional transcriptional units are created by gene recombination events that assemble κ light chains (Igκ), λ light chains (Igλ), and heavy chains (IgH) that comprise Ig molecules by using separate pools of gene segments and exons during B-cell development. Each variable domain (or fragment) of Ig (Fv) is encoded by more than one gene segment. The Fab fragment serves to bind antigen due to its antigen-binding site. The Fc region (crystalizable fragment, Fc) of an Ab is the tail that binds to and interacts with receptors on the surface of effector cells (FcRs). So, a combinatorial rearrangement of the separate gene segments and exons that make heavy and light chains is a crucial, though not the only, mechanism of Abs diversity [43]. This strictly programmed series of somatic gene recombination occurring in the bone marrow during early B-cell differentiation generates mature B-cells from hematopoietic stem cells, resulting in an almost unlimited spectrum of antigen receptors [44]. The second phase of gene rearrangements occurs only with the IgH chains following B-cell activation by an antigen and is known as H-chain class switching [45].

During the adaptive immune response to an antigen, B-cell clones specific to that antigen proliferate intensely, i.e., B-cell clonal expansion starts, which is coupled to somatic hypermutation (SHM). SHM leads to the amassing of mutations in Ig genes, thus generating an even greater number of unique antigen-binding sites on BCRs. SHM takes place in germinal centers (GCs), which are transiently formed microstructures within follicles in secondary lymphoid organs, where mature B-cells are activated, and then proliferate and differentiate, eventually producing long-lived plasma cells that produce Abs and memory B-cells [46]. Despite the tight control over the GC reaction, SHM can still miss the target, so the specific B-cell clones bearing the mutations will gain selective advantages [47]. DLBCLs may also evolve and progress due to errors in the *Ig* genes remodeling during normal B-cell differentiation. DLBCL may slowly progress to a more aggressive state over time owing to clonal evolution, i.e., selective growth and survival benefits of subclones. However, fast growth, such as that following catastrophic intracellular events, cannot be excluded. The latter may produce subclones with extensive DNA rearrangements, which confer a significant survival advantage [46].

Mlynarczyk pointed out that many healthy individuals may carry pre-malignant clones of B-cells. Still, we do not have ways to distinguish those at risk of developing B-cell lymphoma. As expected from neoplasms that originate from cells that normally experience rapid clonal diversification and, when needed, a massive proliferation, GC-derived lymphomas are vastly heterogeneous and can rapidly progress. Other hallmarks of neoplastic B-cells are the inactivation of tumor suppressors and cell cycle checkpoint genes, genome instability and mutagenesis, resistance to cell death, extensive replicative potential, metabolic reprogramming to support massive growth, blockade of terminal differentiation to plasma cells, ability to hide from immune surveillance, and so on [47]. To say it differently, DLBCL aims to hijack the differentiation/maturation events occurring in the GC [48]. The critical difference between the normal and neoplastic B-cells in GC is that the phenotype of the former is solely epigenetically programmed (meaning it can be reversed once the B-cell leaves the GC reaction). In contrast, the malignant phenotype is irreversible due to somatic mutations [49].

## 4. The Cell-of-Origin Distinction of DLBCL Subtypes

In 2018, we learned that 10,000 tumors from 33 tumor types are best classified according to their COO patterns [50]. However, almost two decades earlier, Alizadeh and co-authors, in a groundbreaking paper, suggested that the diversity in the natural history of DLBCL patients may reflect the previously unrecognized molecular heterogeneity of the tumors [51]. GEPs were assessed using DNA microarrays, and two broad molecular subtypes of DLBCL were discovered, each having a distinct gene expression pattern that pointed out different differentiation stages of B-cells: GCB-like and ABC-like DLBCL, with the former predominantly expressing genes characteristic of normal GCB phenotype and the latter expressing those characteristic of mitogen-activated blood B-cells. Of importance was that, of the two subtypes, GCB-DLBCL patients had better OS, which prompted the authors to propose that the two subtypes should be considered separate diseases. Unfortunately, not only did molecular heterogeneity persist within each DLBCL subgroup but a considerable percentage of patients also could not be assigned. As many more DLBCL patients are studied by GEP, more subgroups may emerge [51]. The article earned 8380 citations and is still being cited, showing its great relevance even today. The subsequent study on 240 DLBCL biopsies, subjected to hierarchical clustering to define distinct GEP subgroups, recognized, in addition to GCB- and ABC-DLBCL, a type 3 DLBCL; the latter was different from both COO subtypes [52].

ABC-DLBCL is a phenotypically complex, aggressive lymphoma whose transformation mechanisms still need to be solved entirely [53]. ABC-DLBCL has been long thought to source from B-cells that left the GC, i.e., partially differentiated antigen-secreting plasmablasts. These cell precursors of ABC-DLBCL are reportedly arrested during plasmacytic differentiation, but definite experimental confirmation for this is missing. GEP of the ABC subtype resembles that of end-stage memory B-cells or plasma cells [53,54]. Intriguing new findings support the hypothesis that ABC-DLBCLs actually arise from highly malignant and aggressive memory B-cell populations. Moreover, an interesting hypothesis links ABC-DLBCLs to aged/autoimmune B-cells [53]. We shall certainly not delve deeply into genetic alterations seen in ABC-DLBCL but mention only a few. *PRDM1* encodes BLIMP1 (B-lymphocyte-induced maturation protein 1), a zinc finger transcriptional repressor with a pivotal role in controlling the terminal differentiation of Ab-secreting/effector B-cells [55]. *PRDM1* truncation or homozygous deletions are only encountered in the ABC subtype [56]. ABC cells’ most widely known feature is a heightened incidence of “chronic active” BCR signaling, as opposed to tonic signaling in the GCB subtype [54]. Furthermore, *MYD99* mutations (conferring extra-nodal involvement), *TNFAIP3* inactivation (leading to uncontrollable NF-κB expression), and *NOTCH1* mutations are almost uniquely encountered in the ABC subtype. Unfortunately, the mutations mentioned above are not present in all or the majority of ABC-DLBCL cells, so these are not genuinely ABC-defining traits [54,57]. Otherwise, they could be a starting point for planning a rational, ABC-targeted therapy. In addition, the interferon (IFN) regulatory factor-4 (*IRF4*) gene is often upregulated, thus contributing to the unchecked division of ABC cells; they need this transcription factor (TF) for survival [58]. Interleukin (IL)10 expression, driven by oncogenic canonical BCR/NF-κB signaling, is a general feature of ABC-DLBCLs [59]. The IL10 receptor A gene (*IL10RA*) is amplified in 21% and *IL-10RB* in 10% of primary DLBCLs, whereas IL10 itself was markedly elevated in DLBCLs. Some hypothesized that DLBCLs depend on IL10-STAT3 signaling [60], thus making IL10R a novel therapeutic target. To summarize, the ABC-DLBCL subtype is known for its chronic BCR signaling and constitutive activation of the NF-ĸB pathway [61,62].

Another widely known lymphomagenic driver and transcription repressor is B-cell lymphoma 6 (BCL6), which is a zinc finger protein aberrantly expressed in DLBCL [47], where it was first detected [63]. It acts as a repressor of the tumor suppressor *TP53*, thus helping lymphoma cells to survive [64]. BCL6 is an absolute master regulator of B-cell phenotype. A DNA microarray analysis revealed numerous genes directly repressed by BCL6, including those encoding proteins involved in lymphocyte differentiation, inflammation, and cell cycle control [65]. Both ABC and GCB lymphoma cells are affected by the aberrant expression of BCL6, but the GCB subtype is hit by particular mutations [54]. In GCB-DLBCL, translocations of *BCL2* and/or *c-MYC* are commonly observed [66], leading to the constitutive activation of both c-MYC and the anti-apoptotic protein BCL2. One-fifth of GCB-DLBCLs have gain-of-function mutations of the histone methyltransferase (HMT) *EZH2* gene, which can be seen as a master regulator of the GCB phenotype, although in collaboration with BCL2 and BCL6. GCB-DLBCL is further characterized by the phosphatase and tensin homolog (PTEN) downregulation and phosphatidylinositol-3-kinase (PI3K) upregulation, thus intensifying the PI3K signaling pathway. Upon loss of the *PTEN* or repression of the *PTEN* promoter, phosphatidylinositol-(3,4,5)-trisphosphate (PIP3) accumulates and Akt/mTORC1 becomes activated, further promoting GCB-DLBCL phenotype [54]. Although it was GCB-DLBCL that was first associated with inactivating mutations and, thus, loss of sphingosine 1-phosphate (S1P) receptor-2 (*S1PR2*), *S1PR2* was identified as a tumor suppressor transcriptionally silenced by forkhead box protein 1 (FOXP1) in the aggressive ABC subtype [67]. The mutations in the GCB subtype often hit *CREBBP* and affect E1A-binding protein p300 (EP300) [54].

Signaling cascades operating through BCR in DLBCL, even without an identifiable antigen/ligand, have attracted many researchers, resulting in numerous reports on the distinct roles of antigen-dependent and antigen-independent BCR signaling in the DLBCL subtypes. The viability of ABC cells is strongly dependent on constitutive NF-κB activation, which is the hallmark of this subgroup and likely contributes to its resistance to immuno-chemotherapy. Constitutive BCR signaling in GCB differs from the autoantigen-dependent chronic active BCR signaling in the ABC subtype [68,69,70]. Lymphoma cells can become fully independent of the signaling via BCR and develop alternative activation strategies. Receptor tyrosine kinase-like orphan receptor 1 (ROR1) is the primary suspect in this reprogramming [71]. Absent in most normal tissues, ROR1 overexpression was primarily observed in tumor material from patients with primary refractory DLBCL, Richter’s syndrome, and transformed FL, but not that often in relapsed and non-relapsed DLBCL patients [72]. For a more detailed description of the genetics behind the pathology of DLBCL, the readers are referred to several excellent reviews, e.g., [47,53,68,69,73].

In GCB-DLBCL, when the translocation of *BCL6* co-occurs with the fusion with any of its Ig and non-Ig translocation partners, the enhanced expression of BCL6 follows [74], and this fact has been long known. Still, the mechanism of *BCL6* fragility was unknown until the most recent report on multiple non-canonical DNA structures, such as single-stranded DNA hairpin, triplex DNA, and intramolecular parallel G4, all of them located at the *BCL6* cluster II breakpoint region. These non-canonical structures cause fragility that helps *BCL6* translocation [75]. In an exciting article, recurrent focal deletions of the chr14q32.31-32 locus, correlating with reduced TRAF3 expression in 24/324 dnDLBCL cases, were revealed; furthermore, *TRAF3* loss was associated with enhanced non-canonical NF-κB signaling in primary DLBCL and, also, TRAF-3-deficient DLBCL cells require NF-κB kinase (NIK) for growth and survival. The results corroborated a direct relationship between *TRAF3* genetic alterations and non-canonical NF-κB activation, opening a venue for NIK as a potential therapeutic target in a defined subset of DLBCL [76]. A novel immunological driver in ABC-DLBCL was found, and it was an autonomous BCR signaling associated with the IgM isotype. This intrinsic active BCR signaling mechanism could only be detected through functional BCR testing but cannot be reliably detected by sequencing or GEP, at least not at present. The discovery of this non-genetic oncogenic driver broadens our views on the malignant transformation of mature B-cells. In addition, it can help identify candidate DLBCL patients for the potential inhibition of BCR signaling [77].

## 5. Biological Heterogeneity and Subtyping of DLBCL

DLBCL patients have variable RTT and OS, reflecting this tumor’s biological heterogeneity [78]. The primary sources of DLBCL heterogeneity are different COO and associated genomic lesions; many cell types residing within the tumor mass and in its surroundings (the tumor microenvironment, TME); and different cell states. DLBCL intra-tumor heterogeneity encompasses a selection of genetic events and epigenetic reprogramming of physiological transcriptional programs [79,80,81].

ABC and GCB subtypes comprise up to 85% of cases (Table 2); the others are “unclassified (UC)” [52]. Although somewhat helpful for anticipating the RTT in DLBCL patients treated with ibrutinib [82], the COO system never fully accounted for the variable outcomes following R-CHOP or some other targeted therapy. RTT could differ even when ABC and GCB cases share the same mutations, say a *TP53* deletion and *MYC* mutations [54]. This discrepancy could be because a GEP, employed to stratify a particular case into a COO subtype, gives us a phenotypic description, whereas we need a genuinely genetic description of the malignancy, which is closer to its origin/cause [83]. This issue can be resolved if one clusters the genetic alterations (mutations, translocations, and copy number variations—CNVs) occurring together but does not focus on individual gene/pathway aberrations. The latter seems more or less randomly spread over DLBCL subpopulations [54].

Wright’s classification scheme is a statistical model estimating the probability of DLBCL biopsy samples falling into the COO subtypes (Table 2); being based on the Bayes rule, the model enabled the cases that could not fall into two groups to fall into the UC group [84].

Then, let us mention the work that (i) assumes several subsets of DLBCL with different pathogenetic mechanisms, (ii) employs comprehensive GEP, covering approximately 33,000 genes, (iii) applies different unsupervised clustering methods, e.g., self-organizing maps, and (iv) describes three discrete subsets of DLBCL, which were interpreted based on the affected pathways: the oxidative phosphorylation (OxPhos) cluster, the BCR/proliferation cluster, and the host response (HR) cluster of tumors (Table 2). The hallmarks of these clusters were enrichment in genes encoding for proteins involved in oxidative phosphorylation, mitochondrial activities, and the electron transport chain, and increased expression of multiple components of 26S proteasome in OxPhos; more abundant expression of cell cycle regulatory genes and many components of the BCR signaling cascade in the BCR/proliferation cluster; and the increased expression of T-cell receptor (TCR), natural killer (NK) cell receptors and activation pathway components, complement cascade members, macrophage/dendritic cell markers, and inflammatory mediators in the HR cluster. The third cluster was defined by the associated HR rather than by the intrinsic features of the tumor. Hence, it shares similarities with histologically defined T-cell/histiocyte-rich B-cell lymphoma, which was included in the newest DLBCL classification. The importance of this study is looking beyond genetic aberrations as the cause and fate of DLBCL, introducing a metabolic axis and immune response in the TME, which are reflected through OxPhos and HR clusters, respectively. In addition, rational treatment targets in specific DLBCL subsets were suggested [85]. The study triggered numerous investigations of the molecular and cellular heterogeneity of DLBCL tissues, where genomic and transcriptomic alterations were examined separately or combined with other venues, e.g., the extent of immune cell infiltrations, subtyping the T-cells, B-cells, NK, and macrophage cell populations, and including epigenetic investigations, to mention just a few [86,87].

Schmitz’s/National Cancer Institute (NCI) classification arose from an established relationship between specific clusters of mutations (mutational pattern) and disease course/severity/RTT with R-CHOP or ibrutinib [56]. Leveraging GEP, whole exome sequencing (WES), and DNA copy number analysis, four distinct genetic subtypes appeared: MCD, BN2, N1, and EZB (Table 2), based on the co-occurrence of *MYD88* (L265P) and *CD79B* mutations (MCD); *BCL6* fusion or *NOTCH2* mutation (BN2); *NOTCH1* mutations (N1); and *EZH2* mutation or *BCL2* translocation (EZB). The four subtypes differed in their RTT with R-CHOP, with favorable survival in the BN2 and EZB but inferior outcomes in the MCD and N1 subtypes. Other findings were as follows: ABC-DLBCL patients in the MCD cluster responded pretty well to ibrutinib, a Bruton’s tyrosine kinase (BTK) inhibitor (BTKi); the genetic fingerprint of the N1 cluster appeared in the ABC subtype 95% of the time; and *EZH2* mutations and *BCL2* translocations frequently happened in tandem in GCB [56]. The NCI model classified 46.6% of DLBCL tumors.

Chapuy–Shipp’s classifier/Harward algorithm, relying on the WES in 304 samples of dnDLBCL, created six groups, C0 to C5, each having its own genetic signatures with defining drivers (Table 2). They started with 98 genes with the potential to be tumor drivers, and some of them have already been on the list of mutational drivers such as *TP53*; chromatin modifiers: *KMT2D*, *CREBBP*, and *EP300*; genes encoding for the components of the BCR, Toll-like receptor (TLR), NF-κB, or RAS pathways; other tumor-related genes also affected by genetic alterations such as *KRAS*, *BRAF*, *NOTCH2*, and *SPEN* (Notch signaling modifier); and players in the immunomodulatory pathway such as *B2M*, *CD58*, *CD70*, and *CIITA* [88]. Unexpectedly, a small subset of DLBCLs, C0, lacked defining genetic drivers. Still, this group was enriched with morphologically defined tumors having a brisk inflammatory/immune cell infiltrate, which comprised the HR cluster of Monti et al. (2005) [85]. The absence of detectable drivers in C0 DLBCL still did not mean there were none; other pathogenetic events may have occurred. The Harward algorithm not only “invented” a novel group of low-risk ABC-DLBCLs but also divided GCB cases into two distinct subsets, C3 and C4, each with a different outcome; there was also a group that could not be related to the ABC/GCB division, and their hallmarks were biallelic inactivation of *TP53*, loss of *CDKN2A*, and associated genomic instability. C0/C1/C4 groups meant longer PFS; C2 cases had an intermediate prognosis, whereas C3/C5 had a poor prognosis. A significant proportion of the C1 cluster consisted of ABC tumors [51] and was enriched with *BCL6* structural variants and frequent mutations affecting the NF-κB pathway and *FAS* (fatty acyl synthase); *TP53* mutations and loss of *CDKN2A* and *RB1* occurred in the C2 cluster; the C3 cluster showed *BCL2* mutations and frequent mutations of *KMT2D*, *CREBBP*, and *EZH2*; and C4 showed mutations in four linker genes and four core histone genes. Although C3 and C4 both alter the common pathways such as PI3K, they do so via different mechanisms, which is of enormous interventional significance [54]. Furthermore, C5 was characterized by 18q and chromosome 3 gains and mutations of *CD79B*, *MYD88*, and *Pim-1*, overlapping with the transcriptomic profile of ABC-DLBCL. Additionally, 64% of tumors had at least one structural variant, the most often being translocations that juxtaposed genes to strong regulatory elements. One-quarter of C1 tumors exhibited *MYD88* mutations, albeit these seldom hit Leu265, i.e., they were not *MYD88*^L265P^. In contrast, *MYD88*^L265P^ was a major mutation found in C5 ABC-DLBCL [88].

If we imagine DLBCL as a continuum of evolving neoplastic cells, a strict division into clusters is impossible. This concept explains why one DLBCL may fall into more than one cluster and how different tumor subtypes may be simultaneously present in the same patient. However, there is a system that addresses such challenges, such as Wright’s classification from 2020 [83]. The probability that a particular tumor belongs to the specific genetic subtype may be computed while not excluding the probability that it also belongs to another subtype. For this to happen, a DLBCL tumor must have acquired more than one genetic program during its progression. The authors first defined seven genetic DLBCL classes, with their characteristic genetic aberrations that hit a panel of predictor genes, and then built a classifying algorithm, LymphGen, that determines the probability that a patient’s tumor belongs to one of seven, according to its genetic features. The seven prior-defined genetic classes were BN2, A53, EZB-MYC+, EZB MYC-, ST2, MCD, and N1 (Table 2). Genetically composite tumors were defined as those falling into more than one class. The LymphGen correctly classified 63% of cases used in the study to build the model. The remaining 37% comprised tumors with a few features characteristic of one or more genetic classes but not enough to be classified, then tumors with specific features not recurrent in DLBCL, and tumors with a few genetic features [83]. The reproducibility of the LymphGen algorithm in assigning DLBCL tumors to genetic subtypes was then validated using two cohorts. The first cohort (n = 304), previously used to identify DLBCL subtypes (denoted as “Harvard”) was analyzed for exomic mutations, copy number in selected genomic regions, and *BCL2*/*BCL6* rearrangements [88]. A second cohort (n = 332), used previously to identify signatures of poor prognosis in DLBCL and denoted as “BCC” (British Columbia Cancer agency), was analyzed for mutations in 82 lymphoma-associated genes as well as for whole-genome copy number aberrations [66,89].

A recent report on the mutational landscape in Chinese nodal DLBCL, assessed by targeted next-generation sequencing (NGS), depicted 66% of samples that could not be classified by LymphGen [90]. This finding is of great importance and underscores the need to genetically type many more patients belonging to all known ethnic groups, preferably by comprehensive NGS followed by machine learning (ML), so that we could delve deeper into understanding the genetics behind DLBCL.

Lacy’s or the UK Hematological Malignancy Research Network (HMRN) classifier was designed to group DLBCL cases in a novel way by employing a targeted panel with 293 genes, which were chosen as the hallmark of hematological malignancy according to a large, unselected population-based DLBCL cohort (n = 928) with complete clinical follow-up. Employing Bernoulli mixture-model clustering, five non-overlapping DLBCL genetic clusters were extracted from the data: MYD88, BCL2, SOCS1/SGK1, TET2/SGK1, and NOTCH2 (Table 2). As a rule of thumb with DLBCL, there was a UC group, and this one contained 27% of cases [91]. The more genes analyzed in the cohort, the fewer cases ended up as UC. However, increasing the number of patients brings many more unique mutations affecting just a few, thus enlarging the UC group. The MYD88 cluster was dominated by mutations of *MYD88* (L265P), *PIM1*, *CD79B*, and *ETV6* and frequent loss of *CDKN2A*; the BCL2 cluster showed frequent mutations of *EZH2*, *BCL2*, *CREBBP*, *TNFRSF14*, *KMT2D*, and *MEF2B*. The majority of cases had a t(14:18) of *BCL2*. Many mutations, including *SOCS1*, *CD83*, *SGK1*, *NFKBIA*, *HIST1H1E*, and *STAT3*, appeared in the SOCS1/SGK1 group. An intriguing fact is that the *TP53* mutation had no effect on the NOTCH2 subtype but meant poor prognosis in the MYD88 subtype [91]. Lacy’s genetic classes provided some prognostic ability with 5-year OS rates.

Shen’s classifier, LymphPlex, is a 38-gene algorithm created by using the information on mutations of 35 genes, but it also included information on rearrangements of the famous trio: *BCL2*, *BCL6*, and *MYC*. Six distinct genetic classes were anticipated: TP53^Mut^ (*TP53* mutations); MCD-like (*MYD88*, *CD79B*, *PIM1*, *MPEG1*, *BTG1*, *TBL1XR1*, *PRDM1*, and *IRF4* mutations); BN2-like (*BCL6* fusion, *NOTCH2*, *CD70*, *DTX1*, *BTG2*, *TNFAIP3*, and *CCND3* mutations); N1-like (*NOTCH1* mutations); EZB-like (*BCL2* fusion, *EZH2*, *TNFRSF14*, *KMT2D*, *B2M*, *FAS*, *CREBBP*, *ARID1A*, *EP300*, *CIITA*, *STAT6*, and *GNA13* mutations, with or without *MYC* rearrangement); and ST2-like (*SGK1*, *TET2*, *SOCS1*, *DDX3X*, *ZFP36L1*, *DUSP2*, *STAT3*, and *IRF8* mutations). Half of the patients in the training cohort (that is, 337 dnDLBCL patients taken from the Ruijin cohort encompassing 1001 DLBCL patients) were assigned, while the others were termed NOS (Table 2). The LymphPlex algorithm’s reproducibility was evaluated on the Ruijin cohort (n = 1001), BCC cohort (n = 320), and HMRN cohort (n = 928). Its prognostic relevance was demonstrated, e.g., TP53^Mut^ and MCD-like subtypes had relatively inferior PFS, while ST2-like had relatively favorable PFS [92]. The novel LymphPlex algorithm was concordant with LymphGen developed earlier [83].

Single-cell RNA sequencing (scRNAseq) became the state-of-the-art technology employed to decipher the heterogeneity and complexity of the transcriptome in individual cells. scRNAseq also helps to reveal the composition of different cell types and their functions within highly organized tissues/organs/organisms [93]. An analysis of single-cell transcripts in two DLBCL biopsies disclosed extensive inter-tumor and TME heterogeneity. Seven malignant subpopulations of B-cells were observed, e.g., those exhibiting an IFN response-dominant signature, cell proliferation-dominant signature, metabolism-dominant signature, or hypoxia-dominant signature. In addition, thirteen T-cell subpopulations were also there [94]. The recent single-cell atlas of DLBCL created from transcriptomes of 94,324 cells from 17 DLBCLs using scRNAseq confirmed, once again, the high intra- and inter-tumor heterogeneity, but also brought novel insights into DLBCL pathogenesis [95].

The role of artificial intelligence (AI) in medicine has been expanding, and new ways to analyze the clinical and multi-omics data are being developed almost daily. AI applies computer science to complex datasets and makes predictions or classifications, displaying the immense potential to revolutionize biological research and clinical practice. Carreras employed artificial neural networks (ANNs) to predict the OS and molecular subtypes of 106 cases of DLBCL, leveraging the data obtained from the GEP of 730 genes belonging to the pan-cancer immuno-oncology panel. The ANN model predicted the clinical outcome with high accuracy, with an AUC of 0.98, and ranked the genes according to their importance for the prediction. While *ARG1*, *TNFSF12*, *REL*, and *NRP1* correlated with favorable survival, *IFNA8*, *CASP1*, and *CTSG* were concordant with poor survival, making them high-risk genes. The model subtyped the DLBCLs into GCB, ABC, and UC (81.5% accuracy in the testing set). Interestingly, the model displayed an absolute accuracy of 100% in the training set when “asked” to divide cases into two groups: GCB and non-GCB (ABC + UC), which was a bit lower (96.4%) in the testing set. The top 10 genes extracted for their importance in the binary classifier were *CD37*, *STAT6*, *ATF2*, *ROPN1*, *C4B*, *NOTCH1*, *CTAG1B*, *ICAM3*, *CEACAM1*, and *NOD2* [96]. The premium success of the ANN model in the classification of DLBCL offers a new perspective for future patient stratification into clinically/therapeutically meaningful groups. The same research group then applied ANN to build a model for predicting B-cell NHL types, employing data on 290 NHL cases retrieved from the gene Expression Omnibus (GEO) database. This time, the input data included the complete GEP array of 20,863 genes and a cancer transcriptome panel of 1769 genes. The model successfully classified the cases into FL, mantle cell lymphoma (MCL), DLBCL, Burkitt’s lymphoma (BL), and MZL with an AUC of 0.87–0.99. The model also accurately predicted the OS of 414 DLBCL cases, whose data were retrieved from the GEO (dataset GSE10846) [97].

We are now entirely sure that metabolic adjustments of malignant cells play crucial roles in oncogenesis and tumor progression. A bioinformatics pipeline that can tell malignant from healthy tissues was created based on differential GEP of 114 genes coding for enzymes and proteins engaged in metabolic pathways [98]. From the genomics data on 10,528 tumors of 32 different types, the alterations in genes involved in various metabolic pathways were extracted and leveraged to obtain a pattern of metabolic changes in tumors [99]. It was found that mutations and CNVs of metabolic genes are pervasive across all human malignancies. Moreover, based on the frequencies of metabolic gene alterations, two different tumor supertypes were discovered that were likely to display different RTT and clinical outcomes [99]. The metabolic heterogeneity of DLBCL was reported long ago [100]. The existence of three metabolic DLBCL subtypes, A, B, and C, has been recently reported (Table 2). The classifier was based on the metabolic clustering of the GEP of 1916 metabolic genes, picked up from the seven major metabolic pathways. The added value of this study lies in a prognostic 15-gene model able to predict the potential RTT of the high-risk and low-risk groups to targeted drugs such as BCL2 inhibitor (BCL2i) and PI3K inhibitor (PI3Ki), based on differentially expressed genes among metabolic subtypes [101].

AI is soon expected to be incorporated into lymphoma classification as an additional tool [102]. The avoidance of neural networks due to their “black-box” performance (we do not know how it works) is diminishing, as we currently know how to extract the variables from GEP and clinical data so that we learn which variables contribute the most to the distinction of DLBCL subgroups. These variables are candidates for future research on disease mechanisms and novel therapeutic approaches.

Translating the aforementioned genetic algorithms to a single patient in the clinic is now possible using the LymphGen classifier [83] or the code linked to Lacy’s study [91]. However, the significant challenges are choices: which type of analysis/sequencing is required to assign a patient to a DLBCL group, how much it costs per patient, and which classifier to use [81]. We are confident that deep learning-based software will soon be developed to help assign DLBCLs to the newly discovered classes/subgroups, such as the algorithms described by Carreras and colleagues [96,97,102,103,104].

For the takeaway message, we shall cite Morin et al. (2022): The actual value of a genetic classifier does not lie in its predictive power regarding patient’s response to R-CHOP but instead to identify groups of tumors that may be amenable to specific targeted therapies. Likely, many cases will still not fit into the current genetic subtypes, and we will continue to discover further subtypes [81]. It is high time to include features beyond genomic alterations in the classification schemes for DLBCL subsets, starting from the epigenetic status and host immuno-metabolic status/TME features. Then, we can proceed to the systems-level studies of DLBCL tumors, such as evaluating redox status, the involvement of viral pathogens, and alterations in the proteome, metabolome/lipidome, and N-glycome of cell surface antigens.

Nevertheless, the current state-of-the-art molecular research on DLBCL leaves hope for targeted DLBCL therapy based on differential genetic phenomena happening in DLBCL (sub)populations. An example is a case report on a patient resistant to multiple therapies, including R-CHOP plus enzastaurin and gentamicin + oxaliplatin (GemOx) with lenalidomide and selinexor, who harbored a *CD274* amplification and exhibited a high PD-L1 tumor proportion score. However, after being treated with sintilimab, a monoclonal antibody (mAb) against programmed cell death protein 1 (PD-1), the patient showed a response lasting for 12 months of follow-up [105].

**Table 2:** Genetic and metabolic heterogeneity of DLBCL.

Basis of Classification	Classification	# of Samples & Subtypes Proportions	Subtypes/Subgroups/Clusters	Distinctive Features in Subtypes/Clusters		Classifying Algorithm	Technology/Method of Molecular Profiling	Specimen Type	Gene Expression Signatures/Study Features
cell-of-origin (COO)	COO subtyping; Alizadeh et al., 2000. [51]	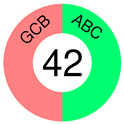	**GCB**	markers of GC differentiation (CD10, CD38), NF A-myb, OGG1, higher BCL-6 mRNA expression		hierarchical clustering	GEP by cDNA microarray (Lymphochip)	Lymph-node DLBCL biopsies	Proliferation signature, GC B-cell signature genes, lymph node genes, T-cell signature genes
			**ABC**	constitutive expression *of IRF-4* and *FLIP*, BCL-2 mRNA levels 4x higher than in GCB subtype					
	COO subtyping; Rosenwald et al., 2002. [52]	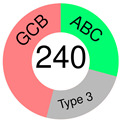	**GCB**	*BCL-2* t(14;18) translocation, *c-rel* amplification exclusively in GCB; *BCL-6*, *CD10*, *LMO2*, *Jnk3*, *A-myb*		hierarchical clustering	GEP by cDNA microarray (Lymphochip)	Lymph-node DLBCL biopsies	16 genes selected from GCB cell signature, MHC II complex signature, stromal & immune cells in lymph-node signature, proliferating cells signature
			**ABC**	*IRF-4*, *FLIP*, *CD44*, *Cyclin D2*					
			**Type 3**	*BCL-2* t(14;18), or *c-rel* amplification not present; do not express either BCG or ABC set of genes at a high level					
	Wright et al., 2003. [84]	(I) 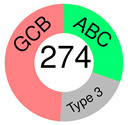 (II) 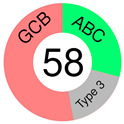	**GCB**	*c-rel* amplification, SHM of *Ig* genes, higher expression of BCL-6, LMO2, MYBL1/A-myb, CD21, CD10, HDAC1, FAK, lck…		linear predictor score for each sample computed and used to estimate the likelihood of a tumor belonging to GCB or ABC	GEP by Lymphochip array or by oligonucleotide microarray Affymetrics HU6500	Data from reference [52] and lymph-node biopsies	GC B-cell signature, MHC II signature (27 genes after selection) [84]
			**ABC**	*IRF-4*; higher expression of *PKCβ1*, *XBP-1*; lower expression of *BCL-6*					
			**Type 3**	samples with <90% likelihood of being in ABC or GCB					
genetic subtyping based on genetic alterations & affected signalling pathway(s)	Schmitz’s/NCI institute classification [56]	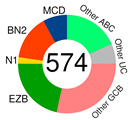	**MCD** 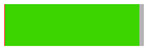	co-occurrence of *MYD88^L265P^* & *CD79B* mutations		automated method starts with a set of seed classes and iteratively moves cases into and out of the classes to optimize a genetic distinctiveness metric	multiplatform genomic analysis using: WES (n = 556), targeted amplicon re-sequencing of 372 genes (n = 530), RNA seq (n = 562), array-based DNA copy number analysis (n = 560)	DLBCL biopsies (574)	79 genes identified whose aberrations characterized each genetic subtype including gene expression signatures were: B-cell differentiation, oncogenic signaling, proliferation & TME
			**BN2** 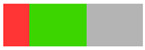	based on *BCL-6* fusions or *NOTCH2* mutations					
			**N1** 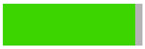	based on *NOTCH1* mutations					
			**EZB** 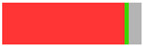	based on *EZH2* mutations or *BCL-2* translocations					
	Chapuy-Shipp’s classifier/Harward algorithm [88]	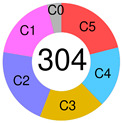	**C0** 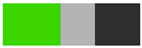	DLBCLs lacking defining genetic drivers; increased numbers of T-cell/histocyte-rich LBCLs		non-negative matrix factorization consensus clustering	hybrid approach: WES expanded to capture known structural variants, DNA copy number analysis, somatic SNVs, Indels	DLBCL biopsies	98 CCGs: *TP53, KMT2D, CREBBP, EP300, BCR* components, *TLR*, NF-κB pathway, *CD79B*, *MYD88, CARD11, TNFAIP3*, RAS pathway components, *KRAS, BRAF, NOTCH2, SPEN, B2M, CD58, CD70, CIITA*; +40 previously undescribed CCGs in DLBCL
			**C1** 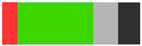	ABC-DLBCLs with genetic features of an extrafollicular, possibly marginal zone origin; enriched with *BCL6* SVs and frequent mutations affecting the NF-κB pathway & *FAS*					
			**C2** 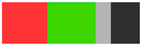	clonal loss of *17p*; *TP53* mutations; *18q21.33* copy gain, loss of *CDKN2A* and *RB1*; *MYC* SVs					
			**C3** 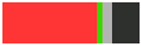	GCB-DLBCLs with *BCL2* mutations with structural variants (juxtaposition *of BCL2* to *IgH* enhancer); mutations in *KMT2D, CREBBP* and *EZH2*; and alterations of *PTEN* epigenetic enzymes					
			**C4** 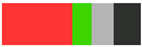	GCB-DLBCLs with mutations in 4 linker and 4 core histone genes; also in *CD83, CD58, CD70*, BCR/PI3K signaling intermediates, NF-κB modifiers (*CARD11, NFKBIE, NFKBIA* and RAS/JAK/STAT pathway members (*BRAF* and *STAT3)*					
			**C5** 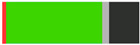	defining mutations of *CD79B MYD88 & TBL1XR1; 18q* copy gains; mutations of *ETV6, PIM1, & BTG1*; highest aberrant SHM					
	Wright’s probabilistic classification (2020)/LymphGen algorithm developed [83]	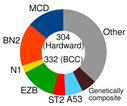	**MCD** 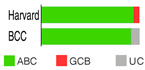	including *MYD88^L265P^* and *CD79B* mutations		GenClass algorithm first used to pre-defined genetic classes; LymphGen, a Bayesian predictor model was built that determines the probability (P) that a patient’s tumor belongs to the subtype; tumors with *p* > 90% are “core” members of subtypes, 50–90% are “extended” members, core members of more than one subtype are “genetically composite”	training data taken from NCI cohort, ref. [56]; LymphGen validation data taken from Harward cohort, ref. [88] and from BCC cohort, ref. [66]		The subtypes showed distinct malignant attributes; MCD & EZB-MYC+ highly expressing signatures of proliferation and MYC activity; N1 expressed a signature of quiescence; EZB-MYC+ tumors highly expressed a ribosomal protein signature; metabolic distinctions between the subtypes included high expression of glycolytic pathway enzymes in ST2 (Warburg effect) and high expression of lipid synthetic enzymes in EZB-MYC+
			**BN2** 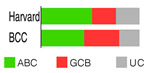	including *BCL6* translocations and *NOTCH2* mutations; resembles MZLs					
			**N1** 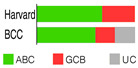	including *NOTCH1* mutations (gain-of-function)					
			**EZB** 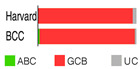	-MYC+	*EZH2* mutations and *BCL2* translocations; with DHIT signature				
				-MYC-	*EZH2* mutations and *BCL2* translocations; without DHIT signature				
			**ST2** 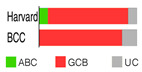	recurrent truncating *SGK1* and *TET2* mutations; inactivating mutations targeting *P2RY8* and *GNA13*; resembles both NLPHL and THRLBCL					
			**A53** 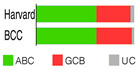	characterized by *TP53* mutations and deletions; frequent mutations or deletions of *B2M*					
	Lacy’s/HMRN classifier [91]	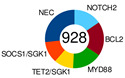	**NEC** 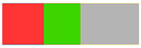	not elsewhere classified DLBCL tumors (27% of patients)		Bernoulli mixtured-model clustering	DNA sequencing, DNA copy number analysis, FISH	DNA extracted from DLBCL tumors diagnosed in patients residing in a catchment population of ~4 million (14 centers) were sequenced with a targeted 293-gene hematological-malignancy panel (n = 928), the HMRN cohort	study confirmed the existence of molecular subtypes of DLBCL; evidence that genomic tests have prognostic significance in non-selected DLBCL given; novelty was the division of the previously identified SGK1 cluster into SOCS1/SGK1 and TET2/SGK1 subgroups; the biological validity supported by the enrichment of *JAK/STAT* and *ERK* gene expression signatures; limitation: the lack of *BCL6* fusion data
			**SOCS1/SGK1** 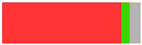	mutations of *SOCS1, CD83, SGK1, NFKBIA, HIST1H1E*, *STAT3*. Several of these targets of aberrant SHM; mutations present common to PMBCL; represents a subdivision of the C4 cluster described by [88]					
			**TET2/SGK1** 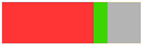	largely GCB-DLBCLs with characteristic mutations including *TET2*, *SGK1* & *KRAS*, representing another subdivision of the C4 cluster [88]; mutation of multiple components of the ERK pathway					
			**MYD88** 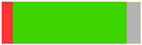	*MYD88* (66.2%), *CD79B* (50.0%), *PIM1* (92.5%), *HLA-B* (73.8%), *BTG1* (70.0%), *CDKN2A* (62.0%), *ETV6* (55.0%), *SPIB* (51.9%), *OSBPL10* (51.2%), *TOX* (48.1%), *BCL2* (48.1%), *BTG2* (43.8%), *MPEG1* (43.8%), *HLA-A* (43.0%), *HLA-C* (42.5%), *SETD1B* (41.8%), *KLHL14* (41.2%), *TBL1XR1* (35.0%), *GRHPR* (33.8%), *PRDM1* (32.5%), *CD58* (31.6%), *TAP1* (26.6%), *PIM2* (25.0%), *FOXC1* (21.2%), *IRF4* (20.0%); encompasses the majority of observed primary CNS lymphomas and primary testicular lymphomas					
			**BCL2** 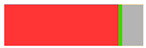	predominantly had characteristic t(14;18) and other mutations in BCL2 pathways; *BCL2* (68.4%), *EZH2* (44.7%), *TNFRSF14* (66.2%), *KMT2D* (53.9%), *CREBBP* (52.7%), *REL* (34.3%), *FAS* (30.1%), *IRF8* (28.9%), *EP300* (27.8%), *MEF2B* (26.3%), *CIITA* (25.0%), *ARID1A* (22.9%), *GNA13* (22.5%), *STAT6* (21.1%), *PTEN* (20.0%)					
			**NOTCH2** 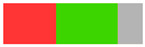	*BCL6* (72.8%), *NOTCH2* (41.8%), *TNFAIP3* (51.6%), *DTX1* (50.0%), *CD70* (41.3%), *BCL10* (39.6%), *UBE2A* (30.4%), *TMEM30A* (26.7%), *KLF2* (21.7%), *SPEN* (21.7%) *					
	Shen’s classifier/ LymphPlex [92]	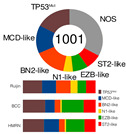	**TP53^Mut^** 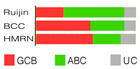	p53 signaling dysregulation, altered PI3K signaling (activation) and lipid synthesis; deficiency of anti-tumor immunity due to low expression of immune signatures including T-cells, NK cells, macrophages, and dendritic cells		a given DLBCL sample was assigned into one of the defined genetic subtypes (TP53^Mut^, MCD-like, BN2-like, N1-like, EZB-like with or without *MYC* rearrangements, & ST2-like) by PAM clustering using mutation data of 35 genes and rearrangement data of three genes: *BCL2*, *BCL6.*	WES (n = 337), WGS (n = 337), DNA copy number analysis, targeted sequencing of the lymphoma-related genes (n = 664); RNA-sequencing (n = 268), FISH	training data (n = 337) taken from Ruijin cohort; LymphPlex-validation data taken from: (i) Ruijin cohort, n = 1001; (ii) BCC cohort [66], n = 320; (iii) HMRN cohort [91], n = 928.	affected were cell proliferation signature, B-cell differentiation genes, B-cell TFs, oncogenic signaling pathways (NF-κB, p53, Notch, PI3K, JAK), TME signature (CD4+, CD8+, T_reg_, NK, M1, M2, DC, stromal-1). TP53^Mut^ and MCD-like subtypes highly expressing signatures of cell proliferation and MYC oncoprotein; TP53^Mut^ and EZB-like-MYC+ subtypes expressing low level signatures of quiescence; metabolic also observed with highly expressing signatures of glycolytic pathway in the TP53^Mut^, MCD-like & BN2-like subtypes; high expressing signatures of lipid synthesis in the TP53^Mut^; classification based on mutations of 35 genes and rearrangements of three genes (*BCL2*, *BCL6*, *MYC*)
			**MCD-like** 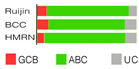	co-occurrence of *MYD88* & *CD79B* mutations, *BCL2*/*MYC* double expression, strong enrichment in ABC-DLBCL signatures, NF-κB activation, IRF4 & MYC upregulation;					
			**BN2-like** 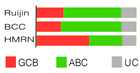	*FBCL6* & *NOTCH2* mutations, signatures of BCR-dependent NF-κB activation					
			**N1-like** 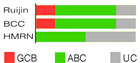	dominated by *NOTCH1* mutations					
			**EZB-like** 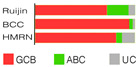	characteristic *FBCL2* & *EZH2* mutations, EZB-like-MYC+ characterized by an immunosuppressive TME; EZB-like-MYC- by NOTCH activation;					
			**ST2-like** 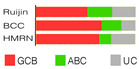	featured with stromal-1 modulation; mutations in *SGK1*, *TET2* & SOCS1; favorable outcome within GCB-DLBCLs					
			**NOS**	166/337 patients with known WES/WGS data (49.3%) not categorized into six subtypes were grouped under NOS					
genetic signature of metabolic heterogeneity	Hou et al., 2023. [101]	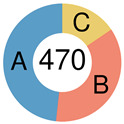	**A**	43 genes downregulated and one gene upregulated in A; 21 genes downregulated and one gene upregulated in B; functional analyses of those DEGs via KEGG pathway and GO analyses were not able to provide any results for clusters A and B (due to the small sizes of DEGs sets)		PAM clustering (unsupervised consensus algorithm) of metabolic genes using the training cohort	microarray GEP, i.e., transcriptomic data of one training dataset (GSE31312) and four validation datasets from DLBCL cohorts GSE10846, GSE53786, GSE87371, GSE23501 were retrieved from the NCBI GEO database		three subtypes of DLBCL identified based on expression levels of 1916 genes engaged in seven metabolic pathways: lipid metabolic pathway (766), carbohydrate metabolism (286), amino acid metabolism (348), the integration of energy pathway (110), nucleotide metabolism (90), vitamin cofactor metabolism (168), TCA cycle (148)
			**B**						
			**C**	102 genes downregulated; 1743 upregulated genes; DEGs in cluster C were more related to neuroactive ligand-receptor interaction, cytokine-cytokine receptor interaction, and calcium signaling pathway					
	Monti et al., 2005. [85]	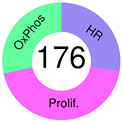	**OxPhos** 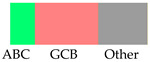	cluster signatures: NADH dehydrogenase complex, COX complex, ATP synthase (mitochondrial), ATP binding protein, ATP binding cassette subfamily, ATPase H+ transporter, TIMM, BFL-1/A1, MIHC, TNFA1P8, FAS, apoptosis-related protein 3, proteasome subunits, PTEN, etc.		three unsupervised clustering algorithms were used and compared: hierarchical clustering, self-organizing maps & model-based probabilistic clustering	GEPs by oligonucleotide microarrays (Affymetrix U133A & U133B) containing probe sets from 33,000 genes, FISH, morphologic analysis of TILs, IHC	DLBCL specimen from 176 patients	increased incidence of t(14;18) in OxPhos tumors; BCR/proliferation cluster had more abundant expression of cell-cycle regulatory genes, increased expression of DNA repair genes and p53, higher levels of many components of the BCR signaling cascade (CD19, Ig, CD79a, BLK, SYK, PLCγ2, MAP4K), additional B-cell–specific or essential TFs; the HR cluster was extensively studied: increased expression of T/NK cell receptor and activation pathway components, complement cascade members, macrophage/DC markers & inflammatory mediators; shared features of histologically defined T-cell/histiocyte-rich B-cell lymphoma, including fewer genetic abnormalities
			**BCR/Proliferation** 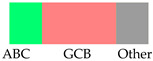	cluster signatures: CD22, CD19, Igμ, CD79α, BLK, SYK, PLCγ2, IP3 R type 3, IP3 kinase B, MAP4K1, CD74; TFS: PAX5, FOXO1A, BCL6, STAT6, NFAT, TCF3, Ikaros, MYC, CD37, BC11A, Ki67, CDK2, p53, H2AX, PTIP, HDAC1, etc.					
			**Host response (HR)** 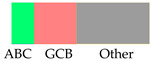	cluster signatures: TCR, CD2, CD3 δ, ε, γ; CD6, Cd28, GATA3, cMAF, CXCR6, NKp30, LAT, FYN, SLAP, LAG3, CD100, perforin, TIRC7, LAIR-1, complement 1qB, 1S, 4A; complement 3α receptor, C1 inhibitor, CD14, CD163, FGR, BIT/SIRPα, granulin, LAMP1, cathepsins B, D; GILT, IFN-induced transmembrane proteins 1, 2; STAT1, HLA A, C, E, F; TNFRS 1A, 1B; IL2R, IL6R, IL15R, Cd31, caspase 4, Notch2, etc.					

Abbreviations: GEP, gene expression profiling; GC, germinal center; ABC, activated B-cell; NF, nuclear factor; OGG1, DNA repair protein 8-oxoguanine DNA glycosylase; BCL, B-cell lymphoma; FLIP, FLICE-like inhibitory protein; SHM, somatic hypermutation; NCI, National Cancer Institute; WES, whole-exome sequencing; TME, tumor microenvironment; SNV, single nucleotide variants; CCG, candidate cancer genes; SV, structural variants; FAS, fatty acyl synthase; MZL, marginal zone lymphoma; DHIT, double hit; NLPHL, nodular lymphocyte-predominant Hodgkin lymphoma; THRLBCL, T-cell/histiocyte-rich large B-cell lymphoma; HMRN, UK Hematological Malignancy Research Network; NEC, not elsewhere classified; CNS, central nerve system; FISH, fluorescence in situ hybridization; PMBCL, primary mediastinal B-cell lymphoma; WGS, whole-genome sequencing; NOS, not otherwise specified; PAM, Partitioning Around Medoids; TFs, transcription factors; Treg, regulatory T-cells; NK, natural killer cells; M1, M2, macrophages; DC, dendritic cells; NCIB, National Library of Medicine; GEO, Gene Expression Omnibus; TCA, tricarboxylic acid pathway; DEGs, differentially expressed genes; OxPhos, oxidative phosphorylation; BCR, B-cell receptor; HR, host response; TILs, tumor-infiltrating lymphocytes; γIFN, gamma interferon; TCR, T-cell receptor; IL2R, interleukin-2 receptor; HLA, human leukocyte antigen, SYK, spleen tyrosine kinase; PLC, phospholipase; IP3, inositol-1,4,5-triphosphate; COX, cytochrome c oxidase; TIMM, translocases of inner mitochondrial membrane; TOMM, translocases of outer mitochondrial membrane; IHC, immunohistochemistry; UC, unclassified; M, missing. * genetic alterations (% of prevalence) according to refs. [54,83]. ABC-DLBCL are in green, GCB-DLBCLs in red, missing (M) in black, and other/UC in gray.

## 6. Genomic Landscape of Diffuse Large B-Cell Lymphoma

The pathogenesis of DLBCL involves a gradual gathering of multiple genetic lesions, eventually changing the structure and/or the expression pattern of proto-oncogenes, tumor-suppressor genes, and other molecules of pathogenetic importance. Data repeatedly show that each DLBCL biopsy typically displays 70 lesions affecting exons, most often mutations and CNVs, with a significant proportion of genes mutating in <10% of patients [73]. We do not intend to delve into a detailed explanation of the genetics of DLBCL since this topic has been heavily covered in the literature [21,47,106]. Mlynarczyk and co-authors have listed the lymphoma-mutated genes for which mechanistic studies have yielded insight into their role in the GC reaction and lymphomagenesis [47].

In a “Herculean effort” to unriddle the DLBCL [107], Reddy and colleagues employed and integrated WES and transcriptomic data with the functional and clinical data obtained by analyzing 1001 DLBCL samples in parallel with the 502 paired germline DNA samples [108]. Quite unexpectedly, the study unveiled only 27 (out of 150) differentially expressed genes in DLBCL vs. normal tissues that had not been identified earlier as implicated in the disease. The most often affected were *KLHL14*, *MGA*, and *SPEN* [107]. The researchers revealed novel, exciting associations, such as the link between *NOTCH2* and cholesterol biosynthesis [108], implying the involvement of lipid metabolism in DLBCL, which will be discussed later.

Mechanisms that give rise to oncogenesis in DLBCL are shared with other tumors and lymphomas, including somatic non-silent point mutations and gene CNVs. Apart from the physiological genetic mechanisms occurring during the lifetime of B-cells, which are *Ig* gene remodeling, class switch recombination, and SHM, the genome of DLBCL is ordinarily altered by two mechanisms that cause genetic damage: inaccurate chromosomal translocations and aberrant SHM [73,109]. Chromosomal translocations in DLBCL tumors usually do not produce fusion proteins; they rather induce aberrant expression of physiologically normal proteins. For example, a proto-oncogene tightly regulated in the preneoplastic history of B-cell may become constitutively expressed in the B-cell lymphoma, such as *BCL6* after its translocation. On the other hand, a proto-oncogene, commonly not expressed in the precursor B-cell, may become activated when this cell turns into a B-lymphoma cell, e.g., upon translocations of *MYC* and *BCL2*, which encode two proteins normally absent in most GC B-cells. Dysregulated SHMs cause hoarding of mutations around the 5′ sequences of genes otherwise not mutated in normal GC B-cells. The physiologic SHM is under the control of high-accuracy repair mechanisms that fight against random mutations of multiple DNA sequences throughout the genome, keeping SHM mostly at the *Ig* loci. However, aberrant SHM in >40 transcribed genes (besides SHM at *Ig* loci) was witnessed in over half of the dnDLBCL samples, irrespective of their molecular subtype, particularly in proto-oncogenes *PIM1* and *MYC*. Now, the panel of genetic changes spotted in each patient often converges on common cellular metabolic and/or signaling pathways, implying their key roles in DLBCL pathogenesis [73,110].

Many GEP studies could not identify molecular signatures robustly correlating with the OS and RTT in DLBCL. Furthermore, if one study reports DLBCL survival-predicting genes, it does not mean the finding will be confirmed across multiple studies. Despite the comprehensiveness of NGS, which offers deep insight into the genetic heterogeneity of DLBCL, it still cannot unveil the full mutational spectrum of these malignancies, likely owing to the low number of patient biopsy samples, which may prevent the discovery of rare transforming genomic lesions [107]. To systematize the ever-growing literature on clinically relevant genomic alterations in DLBCL, NOS, an international expert group was summoned, which reviewed over 200 on-topic articles published from 2008 to 2021. The genes with recurrent abnormalities, which (could) have diagnostic and/or prognostic significance, were listed [21]. Nevertheless, the community seems to be satisfied with 150 non-randomly distributed protein-coding driver genes that are recurrently mutated in DLBCL, apart from genes affected by somatic CNVs [21,47,81]. We urgently need a list of genomic alterations that could aid therapeutic decisions. Through WES, we learned about genetic aberrations in coding regions of genes. However, the non-coding portion of the genome has been neglected, and it likely holds many interesting alterations that may help uncover additional pieces in the DLBCL puzzle. When we finish that, the overall mutation load in DLBCL will undoubtedly be higher.

## 7. Epigenetics and Diffuse Large B-Cell Lymphoma

Epigenetics deals with changes in gene expression excluding those affecting the DNA sequence, occurring as a response to multiple intrinsic or extrinsic events. Epigenetic processes govern cell differentiation via spatial and temporal gene regulation [111]. The epigenetic mechanisms involve DNA methylation, chromatin modifications, loss of imprinting, and various non-coding RNAs (ncRNAs). The epigenetic world is ruled by epigenetic writers, readers, and erasers, all proteins by nature, where writers covalently modify DNA bases and amino acid residues in histones; the expertise of the readers is to identify and interpret those modifications through the attachment to DNA or histones due to the specialized binding domains; erasers are mostly enzymes proficient in removing these chemical tags [112].

Aberrant epigenetics is a hallmark of hematologic malignancies, including B-cell lymphomas [113,114,115]. The perturbation of epigenetic mechanisms lies at the basis of DLBCL [47,114,116]. A comprehensive recent review encompassed the change and function, when known, of each epigenetic molecule that takes part in physiological or oncogenic operations in hematopoiesis, referencing over 750 articles. Moreover, the authors presented therapeutic paradigms based on epigenetic regulation alongside innovative epigenetic-targeted treatments [116]. We shall, therefore, give an overview of several layers of epigenetic changes described in DLBCL while omitting details and focusing on the most recent findings.

Naïve B-cells bear active chromatin marks, which are required to start gene expression when the time comes for B-cells to differentiate into Ab-producing plasma cells upon receiving activation signals. To put it differently, GC B-cells are epigenetically programmed to resemble tumor cells because the GC B-cells are prone to instability in cytosine methylation patterns. Of course, when GC B-cells are converted into DLBCL cells, the latter inherit this epigenetic instability, exhibiting variable degrees of epigenetic heterogeneity [113]. The greater the epigenetic heterogeneity, the worse the clinical outcome [113,117]. The epigenetic landscape of the GC reaction is concerted by the major transcription repressor BCL6 and dominated by transient silencing of promoters and enhancers of genes engaged in proliferation checkpoints, BCR and CD40 signaling, IFN response, antigen presentation, and differentiation into plasma cells. BCL6 is induced solely in the GC reaction but turned off upon GC exit [49].

Histones reside in the cell nuclei, serving as spools around which DNA winds to create nucleosomes, which, in turn, wrap into nanofibers, thus forming tightly packed chromatin. Not only do histones prevent DNA damage but they also attend to gene regulation and DNA replication. H1 and H5 are linker histones; H2, H3, and H4 are core histones. Histones are alkaline proteins abundant in positively charged lysine (Lys) and arginine residues, which enable them to keep close to DNA strands via electrostatic interactions with negatively charged phosphate groups across DNA [118]. On their side, histones are often post-translationally modified by acetylation, methylation, phosphorylation, ubiquitynation, etc. [119,120]. The acetylation of Lys in histones diminishes its positive charge, thus weakening the histone/DNA interaction and causing open euchromatin status, which becomes ready for transcription. That is why histone acetylation marks are often seen in promoters and enhancers of actively transcribed genes. Histone modifications are essential for hematopoiesis and lymphomagenesis [116].

One particular family of epigenetic writers plays a pivotal role in sustaining normal hematopoiesis, which is the p300/CBP family. Two proteins from the family important for DLBCL are the CREB-binding protein (CREBBP, or just CBP or KAT2A) and EP300 (P300 or KAT2B). These enzymes acetylate histone H3, so they are histone acetyltransferases (HATs), which operate as transcriptional cofactors, regulating gene expression [121]. CREBBP/EP300-containing complexes are critical for the initiation, progression, and immune responses to DLBCL [116]. *CREBBP*/*EP300* abnormalities are frequent in DLBCL and include gene mutations, CNVs, and structural variations [116,122,123,124]. Mutations in *CREBBP* were found in up to 30% of DLBCL patients [56,125], while mutations in *EP300* occur in 5.4–9.7% of DLBCL [56]. Epigenetic readers recognize histone acetylation due to the highly conserved binding domain-bromodomain, but evidence of the role of readers in hematological malignancies is still lacking.

Histone deacetylases (HDACs) are erasers that remove the acetyl groups from Lys. In hematological malignancies, the abundance of the HDAC protein, i.e., protein expression, is altered, not mutations affecting the *HDAC* [116]. HDAC1 and HDAC2 are upregulated in DLBCL compared to normal lymphoid tissues [116,126].

KMT2D is a family member of histone H3 Lys4 (H3K4) N-methyltransferases [127]. Highly recurrent mutations of *KMT2D* occur early in DLBCL lymphomagenesis, thus perturbing B-cell development in GC [128]. In B-cells, KMT2D maintains the H3K4 acetylation mark at specific enhancers and enables their functionality. These enhancers, in turn, normally respond to CD40 and BCR signaling. Therefore, the lack of functional KMT2D leads to the aberrant expression of CD40 and BCR, the failure of B-cells to differentiate and exit the GC reaction, and eventually, lymphomagenesis [128,129].

The consequence of inactivating somatic mutations of *CREBBP* is promoting the role of HDAC3, which encourages the development of HDAC-3-dependent lymphomas [124]. The mechanism behind the role of HDACs in tumorigenesis and the progression of DLBCL has yet to be dissected. However, the expression of several HDACs was significantly higher in the lymph node samples of DLBCL than in whole-blood cell controls [130]. *CREBBP* and *KMT2D* mutations frequently come in tandem, maybe owing to an oncogenic cooperation, and this phenomenon was observed in cohorts of the EZB/cluster 3 subset of DLBCL (Table 2). A severe lymphoma phenotype was produced upon the synergistic appearance of these two mutations in mouse phenotype vs. either allele alone. In addition, the phenotype brought an immunoevasive TME [131].

Enhancer of zeste homolog 2, EZH2, is the enzymatic catalytic subunit of polycomb repressive complex 2 (PRC2), which is responsible for establishing and maintaining histone H3K27 (tri)methylation during cell differentiation and proliferation. *EZH2* has a dual role in different hematological malignancies. It can also operate as an oncogene owing to the gain-of-function mutations affecting its catalytic domains. That way, EZH2 amplifies H3K27 methylation, which stops B-cell evolution to plasma cells, i.e., lymphomagenesis [113,116]. Somatic mutations of *EZH2* have been massively identified in hematologic malignancies [132,133]. Heterozygous somatic mutations of *EZH2* are encountered in up to 30% of DLBCLs and are usually missense mutations that lead to the inactivation of the EZH2 catalytic domain [108].

DNA is methylated almost exclusively at the C5 position of cytosine in CpG dinucleotides (CpG sites), which is performed by DNA methyltransferases (DNMT). The CpG sites are unevenly distributed throughout the genome, usually accumulating in clusters known as CpG islands. Two things to know about normal cells under physiological conditions are that they have mostly unmethylated CpG and 70% of gene promoters harbor CpG islands [116]. The aberrant dynamics of DNA base methylation (DNAm) opens another venue for the emergence of a hematological malignancy, but it often has a helping hand from another epigenetic error [134,135]. DNMT1 keeps an established DNA methylation scheme, and DNMT1 protein abundance is increased in half of all DLBCLs [136].

DNA demethylases, including ten-eleven translocation (TET) methyl-cytosine dioxygenase, are epigenetic erasers that remove the methyl groups from CpG sites [116]. TET2 is a member of the TET family of proteins. *TET* mutations and/or dysregulation of TET proteins contribute to the development of multiple hematological malignancies [137]. *TET2* is a DLBCL tumor suppressor gene. *TET2* mutations in DLBCL are among the earliest, occurring in HSCs, setting the stage for B-cell lymphoma, albeit in circa 10% of patients [138,139]. *TET2* and *CREBBP* mutations are, in the majority of cases, mutually exclusive, so *TET2* loss-of-function generates lymphoma cells that show a dependence on HDAC3 [140]. Cytosine methylation variability defines six new DLBCL subsets with significantly different clinical outcomes in human DLBCL [141]. In their latest study, Carlund and co-authors dissected DNAm profiles in 66 DLBCL, NOS tumors, with the significant findings as follows: DLBCL is characterized by sizeable DNAm diversity in comparison to normal cells and other B-cell neoplasms; DNAm patterns did not differ between GCB- and non-GCB malignancies; and ample global hypomethylation in DLBCL cases under R-CHOP-like regimens correlated with the worse disease-specific survival. The authors hypothesized that DLBCL cases with poor prognosis and high global hypomethylation could be candidates for alternative treatment regimens, including hypomethylating agents [142].

All RNAs that will not be translated into proteins are under the umbrella of ncRNA [143]. To add to the complexity of the ncRNAs landscape, recent studies reported some ncRNA transcripts translated into small peptides [144]. Originally, ncRNAs were thought to regulate gene expression at the post-transcriptional level. Besides that, some considered ncRNAs as “junk” RNAs. Currently, findings on the role of ncRNAs in NHL are on the rise [145]. ncRNAs are divided into housekeeping and regulatory ncRNAs. The latter is broadly divided into two categories: short-chain ncRNAs, including small inhibitory RNAs (siRNAs), microRNAs (miRNAs), and piwi-interacting RNAs (piRNAs), and long non-coding RNAs (lncRNAs). Intensifying research has been focusing on the link between miRNAs and human neoplasms [146]. A recent review presented a comprehensive list of dysregulated lncRNAs in malignancies and also described the roles of lncRNAs and circular RNAs (circRNAs) in NHL etiology, including DLBCLs. The review mentioned a therapeutic anti-tumor potential that lies in oligonucleotide-based agents such as specific anti-sense oligonucleotides, small interfering RNAs, and small-molecule inhibitors, SMIs [145].

LncRNAs are generally over 200 nucleotides in length, may reside both in the nucleus or cytoplasm, and are rarely translated into proteins [146]. Emerging studies imply their participation in the mutual regulation of cells in TME, thereby affecting the anti-tumor immune response [147]. The expression of lncRNA during B-cell evolution in humans was investigated, and 272 long intronic RNAs, 471 anti-sense RNAs, 376 pseudogene RNAs, and 64 lncRNAs were found in association with distinct stages of B-cell development [148]. The ground study of lncRNAs in DLBCL uncovered 2632 novel multi-exonic lncRNAs in 116 samples of dnDLBCL tumors. Two-thirds of lncRNAs were uniquely present in DLBCL tissues, i.e., could not be detected in normal B-cells. Noteworthily, one-third of lncRNAs were differentially expressed between the ABC and GCB subtypes and enriched at DLBCL super-enhancers. Interestingly, point mutations in lncRNAs frequently stabilized their structures instead of destabilizing them. The authors anticipated that these novel lncRNAs represent new targets for anti-sense oligonucleotide pharmacology [149]. Starting in 2017, a flood of articles on various aspects of lncRNAs in DLBCL samples surfaced. As expected, and following the previous patterns, many of these studies attempted to create various predictive formulas based on the lncRNA signature instead of GEP data. Let us mention just a few, e.g., SubSigLnc-17 could discriminate GCB from ABC subtypes. SubSigLnc-17-based patient clusters had significantly different clinical outcomes. Then, an epigenetic signature based on nine lncRNAs (ELncSig) could predict the five-year OS independently of traditional prognostic factors in DLBCL patients [150]. Particular roles of lncRNAs in the pathobiology of DLBCL have been recently discovered: lncRNA termed FIRRE is linked to BCR stabilization, thus facilitating DLBCL development [151]. Finally, DLBCL patients had increased serum levels of two lncRNAs, HOTAIR and HOTTIP, which positively correlated with unfavorable clinical outcomes, opening a window for potential serum biomarkers [152]. In a recent systematic review, the capacity of lncRNAs to become diagnostic and prognostic biomarkers in aggressive B-cell NHL, including DLBCL, was elaborated [153]. We shall soon witness the appearance of articles suggesting that lncRNAs may become new immunotherapeutic targets, and many more of those dealing with the prediction algorithms for RTT with R-CHOP and similar regimens.

miRNAs are small non-coding RNA (sncRNAs) engaged in gene silencing and control of post-transcriptional gene expression programs. miRMAs may also be involved in tumorigenesis [154], including DLBCL [155,156]. The investigations of miRNAs in DLBCL started with testing whether some species of miRNA are differentially expressed in the serum of (n = 60) DLBCL patients vs. healthy persons. Three miRNAs were chosen for the comparisons: tumor-associated miR-155, miR-210, and miR-21. miR levels were higher in patients than in the control sera, and increased miR21 abundance was associated with the relapse-free survival. This work first described circulating miRNAs as candidates for non-invasive diagnostic markers of DLBCL and possibly other malignancies [156]. A recent review (2024) shed light on the involvement of miRNAs in adjustments of immune checkpoints such as PD-1/PD-L1 (ligand of PD-1) and cytotoxic T-lymphocyte-associated protein (CTLA)-4 [157]. Potentially significant miRNAs in DLBCL were systematically reviewed, and the authors listed a total of 140 differentially expressed miRNAs in DLBCL tumor samples vs. controls, of which 93 miRNAs were differentially expressed in ABC- and GCB-DLBCL; three miRNAs were found to be associated with a favorable RTT [158].

siRNAs, sometimes termed short interfering RNAs or silencing RNAs, are derived from long double-stranded (ds)RNAs, including RNAs originating from virus replication, transposon activity, or gene transcription. The Dicer enzyme usually cuts long dsRNAs into 19–24 nucleotide-long fragments. The latter exercise their functions after being loaded onto Argonaute proteins. siRNAs hinder the expression of genes that have complementary nucleotide sequences and do it by degrading mRNA after transcription, the classic example of post-transcriptional gene silencing. RNA interference is expected to become a novel approach in treating various hemato-oncological conditions because of the siRNA specificity and efficiency in knocking down disease-related genes. Major obstacles to be solved include the low cellular uptake of siRNAs and their susceptibility to nuclease-mediated degradation [159].

CircRNAs are relatively novel endogenous lncRNAs, with their 5′ and 3′ termini covalently linked [160]. CircRNAs appear to be ubiquitously expressed in mammalian cells since thousands have already been discovered in the eukaryotic transcriptome. CircRNAs show cell type-, tissue-, and developmental-specific expression [160,161]. These RNAs may operate as miRNA sponges, regulators of splicing and transcription, or modifiers of parental gene expression [161]. Evidence shows them taking part in adjusting the resistance of cells to anti-tumor immunotherapy. Certain circRNAs may sponge specific miRNAs or interact with proteins, thus changing the expression of immune-related genes and crucial immune checkpoint molecules. These events, in turn, shape the TME and significantly impact the response to immunotherapy [162]. CircPCBP2 was increased in human DLBCL specimens and DLBCL cells in culture, accompanied by decreased levels of miR-33a/b. The important findings in the context of strengthening the therapy of DLBCL were that circPCBP2 promoted the stemness of DLBCL cells and reduced their responsiveness to CHOP treatment due to capturing miR-33a/b, thereby facilitating the upregulation of PD-L1. The opposite result was also displayed, whereby the knockdown of circPCBP2 (or overexpression of miR-33a/b) interfered with the stemness of DLBCL cells, thus promoting tumor cell apoptosis upon CHOP treatment [163]. piRNA-30473 contributes to DLBCL tumorigenesis and poor prognosis by regulating m6A RNA methylation [164].

With NGS leaping forward, over 160 covalent RNA modifications were detected in each RNA type. Hence, a novel layer to the gene regulation scene has been introduced, resulting in novel RNA epigenetics, or epitranscriptome [165]. Pseudouridine, N7-methylguanosine, 5-hydroxymethylcytosine, 5-methylcytosine, N1-methyladenosine, and N6-methyladenosine (m6A) are the most common chemical alterations in RNA, but this list will sure grow. The concerted work of epigenetic tandem, writer + eraser, RNA methyltransferase, and RNA demethylase can reversibly methylate RNA. The “readers” are proteins that specifically recognize modified RNA and stabilize its methylation sites, thus influencing the splicing, stability, or translation of modified RNAs [165,166]. m6A type RNA methylation is involved in the metabolic reprogramming of tumor cells [167]. A recent report explained how dysregulated methylation of RNA at m6A in tumor cells destroys immune cells and facilitates immunosuppression in TME, thereby regulating tumor immune evasion [168]. Considering that epitranscriptomics is a relatively novel research field, we expect more studies to follow on the involvement of modified RNAs in B-cell lymphomagenesis and progression, as well as in RTT. One such study revealed that FAT1, which inhibits the division of DLBCL cells, operates by facilitating the m6A modification of the YAP1 mRNA [169].

Tumor antigens beyond the human exome are particularly promising neo-epitopes for targeted therapies since the expression of neo-antigens is tumor-specific. The most recent survey explained the diverse processes that create canonical, noncanonical, and viral neo-epitopes. Canonical neo-antigens are well-known since they originate from somatic mutations. Moreover, noncanonical and viral neo-epitopes present exciting future targets for anti-neoplastic drugs [170].

## 8. Hitting Epigenetic Targets in Diffuse Large B-Cell Lymphoma

Epigenetic diversity accompanies the immense genomic diversity of DLBCL. Therefore, different cell populations in DLBCL tumor tissues display a broad spectrum of epigenetic modifications throughout the DNA or at particular genes [113]. Indeed, the epigenetic diversity in DLBCL brings an additional fitness to tumor cells, regardless of the underlying genetics. With that in mind, it is no wonder that epigenetic therapies have appeared as promising new therapeutic modalities for DLBCL patients [116]. Epigenetic anti-tumor strategies chiefly exploit SMIs that target epigenetic writers, mostly DNMT, readers, e.g., the bromodomain and extra-terminal-containing (BET) protein family, and erasers such as HDACs [112]. Two drugs that inhibit DNMT (DNMTi), azacitidine and decitabine, were approved by the US Food and Drug Administration (FDA) and the European Medicines Agency for treatment of myelodysplastic syndrome and acute myeloid leukemia (AML). Whereas evidence that decitabine may promote hypomethylation in DLBCL is still lacking [171], there is a recent report on priming DLBCL cell lines to the venetoclax-induced killing by the pre-treatment with decitabine. The effect of decitabine was due to the suppression of the pro-survival PI3K/Akt pathway. Decitabine induced the dependence of DLBCL cells on BCL2, thus augmenting the action of BCL2i, venetoclax. The venetoclax + decitabine combination hampered DLBCL growth both in vitro and in vivo; hence, it is worth testing in clinical settings in patients with DLBCL [172].

The function of an HMT, EZH2, in the GC formation is well-documented. Tazemetostat is a selective and reversible small-molecule EZH2 inhibitor (EZH2i), approved for treating adults with R/R FL after at least two prior therapies, but only when there is a documented *EZH2* mutation or no satisfactory alternative drug [173]. DLBCLs with *EZH2* mutations manifest silencing of *MHC I* and *MHC II* and reduced CD4+ and CD8+ tumor-infiltrating lymphocytes (TILs), hence introducing EZH2i in the standard of care therapeutic regimen for DLBCL may be beneficial [174]. The tazemetostat + venetoclax combination significantly enhanced death in a panel of DLBCL cell lines, including those with *EZH2* mutations. The effect was much stronger than the effect of either drug alone. The combination was most effective in DLBCL cells carrying *EZH2* and *BCL2* alterations, but the effect was missing in cells free of these genetic alterations [175]. This study, once again, spoke of the need to explore potential precision therapies in DLBCL patients who harbor specific gene alterations. In line with this work, tazemetostat exerted a favorable safety profile in the phase II clinical trial with three patients with the *EZH2* mutation and DLBCL, but the patients achieved only a partial response [176]. Another study enrolled 17 dnDLBCL patients with poor prognosis, who were given tazemetostat with R-CHOP, but the clinical outcome was comparable to that of R-CHOP alone [177].

Chidamide, epidaza, or tucidinostat is the world’s first inhibitor of HDAC (HDACi), developed and approved in China for treating R/R peripheral T-cell lymphomas [178]. The transcriptomic landscape of DLBCL cells subjected to chidamide profoundly changed because of the drug-induced overexpression of more than 2000 mRNAs, including those coding for proteins involved in raft/cytoskeleton formation and internalization. The significant finding of the study is that the co-treatment of DLBCL cells with chidamide and RTX counteracted RTX-induced loss of CD20, which was also shown in the mouse xenograft model of DLBCL [179]. The ability of chidamide to kill DLBCL cells in culture was confirmed by others, who demonstrated that chidamide operated in a dose-dependent manner and interfered with the activation of STAT3 [178]. Chidamide impacted the PI3K/Akt/mTOR signaling cascade, too. However, the expression of most HDACs, such as HDAC1, HDAC2, HDAC3, and HDAC10, was not altered in chidamide-treated cells, which meant that chidamide inhibited the catalytic activity of HDACs, but not their expression directly [130]. The therapeutic potential of chidamide was demonstrated in a clinical trial where elderly dnDLBCL patients, who very often experience poor clinical outcomes and cannot endure intensive chemotherapy, were given chidamide plus R-CHOP (CR-CHOP). CR-CHOP was proven to be an effective and safe regimen for 49 patients, where the complete response (CR) and overall response rate (ORR) were 86% and 94%, respectively. The negative prognostic effect of *CREBBP*/*EP300* mutations was mitigated by CR-CHOP [180]. The experimental and clinical data on chidamide-based therapeutic modalities for hematological malignancies were compiled in a recent review [181].

Predicting patients’ responses to epigenetic inhibitors is very difficult because our knowledge of epigenetic mechanisms in hematological malignancies and DLBCL is still limited. The drugs targeting precise epigenetic writers, readers, or erasers will likely be used in combination with other therapy regimens, most likely immunotherapeutics. The epigenome consists of many layers of functionally interdependent mechanisms. Hence, it is challenging to fully correct aberrant epigenetic programs by targeting only one target [113,114,115]. When we investigate the biological response of DLBCL cell lines to experimental epigenetic drugs, the findings cannot be directly translated into human DLBCL, mainly owing to the absence of TME in cell cultures. The TME affects every aspect of tumor development, progression, and immune evasion. Mouse xenograft models, on the other hand, offer insight into the interplay of the TME and malignant cells, but mice are not humans, so the more we move from genome to phenotype, the bigger the differences. Epigenetics is also very different between humans and mice. A recent article comprehensively compared the epigenetic events between mice and humans, breaking new ground and strongly suggesting that scientists should make informed decisions regarding the suitability of mouse models for their experiments [182].

## 9. Targeting Major Pathways in Diffuse Large B-Cell Lymphoma

We shall briefly describe the chief signaling and metabolic pathways often affected by DLBCL. Basic knowledge of these pathways is needed to understand the modus operandi of the anti-tumor therapeutics used to treat DLBCL. The mutated genes and/or altered epigenetic mechanisms and events may enhance or inhibit the pathways.

### 9.1. B-Cell Receptor Signaling

The signaling cascade triggered by the activation of BCR is one of the best-known pathways in humans, and data on BCR signaling have been described with a high level of granularity. BCR signaling supervises transcriptional programs associated with B-cell activation, cell fate decisions, and the BCR-dependent antigen presentation of T-cells [183]. BCR is a transmembrane receptor that protrudes from the surface of a B-cell into the extracellular space. BCR comprises a membrane-bound Ig (mIg) and a signal transduction moiety, Igα/β. Oligomers of dozens of ‘monomeric BCR units’ residing within so-called protein islands in the plasma membrane were observed through imaging [184]. Based on these findings, it was postulated that the activation of intracellular signaling cascades occurs upon the opening/dissociation of preformed BCR oligomers after the BCR stimulation. These events lead to the unmasking of the immunoreceptor tyrosine-based activation motifs (ITAMs), which are located in the intracellular Igα (or CD79A) and Igβ (or CD79B) domains. Then, ITAMs are phosphorylated by a series of cytoplasmic protein tyrosine kinases [185,186]. A B-cell is triggered when its BCR encounters, for the first time, its ‘cognate antigen’. BCR then binds to the antigen, resulting in B-cell clonal proliferation and differentiation, giving rise to a population of Ab-secreting plasma cells and memory B-cells. Two types of signals may emanate from the activated BCR. One is a continuous, low-strength ‘tonic’ signal, which occurs via PI3Kα and PI3Kδ and is assumed to be independent of antigen binding; the second is the so-called ‘active’ signal that moves forward in response to antigen binding and employs a plethora of downstream signaling molecules, primarily proteins and lipid mediators, to support the proliferation, survival, and differentiation of B-cells [186]. There is evidence of DLBCL hijacking both active/chronic and tonic BCR signaling [47]. The widely acknowledged feature of the ABC-DLBCL subtype is its chronic active BCR signaling, proceeding via the BTK/CARD11 pathway; hence, the deletion of BTK is deleterious for ABC-DLBCL cells [61].

On the other side, the C3 and EZB subsets of GCB-DLBCL subtype, instead, are enriched with errors that indirectly affect the PI3K pathway, being closer to ‘tonic’ BCR signaling [47,187]. In brief, physiological signaling in B-cells starts with BCR binding to its matching ligand; a dimer CD79A/CD79B is formed, with their ITAMs being phosphorylated; spleen tyrosine kinase (SYK) is then recruited and SYK activates BTK, which spreads the activation signal downstream, unfolding the chain of events, including the activation of NF-κB and mTOR pathways [188]. BTK is a non-receptor tyrosine kinase that resides in the cytoplasm but is recruited to the plasma membrane when needed; once there, BTK can become activated by the interaction with SRC kinases or SYK [189].

### 9.2. Targeting Bruton’s Tyrosine Kinase

BTK is a critical downstream kinase of the BCR signaling pathway [186,188,189]. Aberrant BTK signaling is a master oncogenic driver in a spectrum of B-cell malignancies [188]. Research on BTK has been gaining momentum since the approval of BTK-targeted inhibitors as a first-line treatment for B-cell lymphomas. Cysteine 481 (C481) in the ATP-binding pocket of BTK is the target of many BTKi [190,191], which will be described.

Ibrutinib is a small-molecule BTKi of the first generation, which attaches covalently (irreversibly) to C481, therefore inhibiting the BCR pathway downstream of BTK. Ibrutinib is an FDA-approved, orally administered drug usually used to treat transplantation-ineligible adults after at least one prior therapy [191]. The tandem administration of ibrutinib with other anti-neoplastic drugs, e.g., lenalidomide and/or RTX, is more effective than ibrutinib alone [192]. Ibrutinib produces high response rates in ABC-DLBCL; these patients benefit from ibrutinib therapy owing to the selectively acquired mutations that foster chronic active BCR signaling in ABC-DLBCL. Exceptional RTT with ibrutinib has been noted in ABC-DLBCL subsets harboring tandem *CD79B* and *MYD88*^L265P^ mutations. Phelan et al. unriddled the molecular scheme behind the oncogenic BCR signaling, which is controlled by a multiprotein MYD88/TLR9/BCR complex. These data illustrate a possibility for the rational design of SMIs of the proteins involved in oncogenic signaling cascades, albeit in molecularly defined subsets of DLBCL [193]. There is evidence of ibrutinib modulating the TME to overcome immunoevasion mechanisms [194]. BTKi inhibits the BCR signaling pathway and other downstream pathways, e.g., NF-κB, MAPK, NFAT, and mTOR. The inhibition of these cross-linking pathways is accompanied by the activation of effector T-cells and eventually leads to tumor eradication. The limitations to ibrutinib application in DLBCL are off-target toxicity and the development of resistance. The accurate mechanisms of the resistance of DLBCL to ibrutinib need further investigation [195]. Of note, a C481S mutation of *BTK* was detected in ibrutinib-resistant patients [196].

Whereas all the second-generation covalent BTKis, namely, tirabrutinib, acalabrutinib, and spebrutinib, bind to the same residue in BTK, C481, tirabrutinib is the most selective in terms of hitting potential off-target enzymes (Table 3). The latter encompasses over ten tyrosine kinases that contain a Cys at a position homologous to that of C481 in BTK, such as BMX (bone marrow-expressed kinase), EGFR (epidermal growth factor receptor), ITK (interleukin-2-inducible T-cell kinase), and TEC. The nuances in the selectivity of BTKi for binding off-target kinases could potentially be translated into improved clinical efficacy and safety [195]. The anti-neoplastic mechanisms of tirabrutinib against ABC-DLBCL cells and xenograft model were investigated by phosphoproteomics and transcriptomics. Tirabrutinib, but not ibrutinib, was selective in targeting only B-cells. It was encouraging to learn that tirabrutinib targeted only four off-target kinases, namely TEC, BMX, HUNK, and RIPK2, which, according to the known literature data, are not involved in the pathology of hematological malignancies [197]. A phase Ib trial of acalabrutinib (NCT02112526) demonstrated a 33% ORR in R/R ABC-DLBCL patients vs. 16.7% in R/R GCB/UC cases [198], similar to the results obtained in trials of effects of ibrutinib and tirabrutinib in R/R DLBCL patients [199]. TL-895 (formerly M7583) is a potent, highly selective, second-generation irreversible BTKi that interferes with the growth of a subset of activated DLBCL cell lines. TL-895 also negatively affected the tumor growth in 5/21 DLBCL xenograft models. Importantly, since TL-895 did not inhibit the Ab-dependent cellular cytotoxicity (ADCC) triggered by the RTX, these two drugs can be used in tandem [200].

Combinatorial approaches might be a better strategy to accomplish deeper and faster remission in R/R DLBCLs, particularly for treating aggressive subtypes of B-cell lymphoma [195]. A phase II clinical trial of R2A used a combination of RTX, lenalidomide, and acalabrutinib, and patients with aggressive R/R B-cell NHL, mostly DLBCL, demonstrated a 1-year OS rate of 67.5%, with controllable adverse events (AEs). Moreover, patients harboring *MYD88* mutations (known for NF-κB activation) and elevated BTK expression responded well [201]. In an interesting novel work that combined an epigenetic inhibitor—AZD5153—and a BTKi—acalabrutinib, in the pre-clinical models of ABC-DLBCL, it was observed that AZD5153 enhanced the efficacy of BTKi, operating through PAX5 (TF for BCR signaling genes) and BCR itself. The synergism of the two inhibitors can be briefly described as follows: while AZD5153 decreased PAX5 expression, acalabrutinib disrupted the BCR signaling, thus inhibiting PAX5 activation [202]. The synergistic action of chidamide and BTKi orelabrutinib in killing DLBCL cell lines in culture justifies their further exploration in clinical settings for the treatment of R/R DLBCL [203].

### 9.3. Targeting NF-κB Signaling

Nuclear factor NF-κappa B signaling (NF-κB) is recruited transiently when normal B-cells respond to antigens. However, B-cell-derived lymphomas accumulate genetic lesions, leading to the constitutive activation of the NF-κB signaling cascade. Many factors, including persistent infections, the pro-inflammatory TME, immune receptors turned against auto-antigens, and mutations that alter the functions of key signaling effectors, may promote constitutive NF-κB activity in DLBCL [204].

NF-κB signaling pathways in lymphocytes may be triggered in response to upstream antigen receptors, tumor necrosis factor (TNF) receptors, IL1Rs, and TLRs, so the NF-κB cascade plays a critical role in the development, survival, and acquisition of lymphocyte effector functions. Some of the vital functions of NF-κB proteins require their homo- or heterodimerization, their interaction with an inhibitor of κB (IκB) proteins, and their movement to nucleus, where they bind DNA. NF-κB activation in response to extracellular hints may proceed by canonical or non-canonical pathways. In the canonical NF-κB activation pathway, the IκB kinase (IKK) complex becomes activated, leading to the phosphorylation and subsequent ubiquitin-mediated degradation of the IκB proteins in proteasome. With no inhibitory proteins to keep them confined in the cytoplasm, NF-κB proteins translocate to the nucleus, where they operate as TFs. Many lymphoid malignancies erroneously activate NF-κB because of the genetic lesions that affect critical proteins in the NF-κB signaling cascade [204,205]. ABC-DLBCL, too, has been associated with aberrant NF-κB signaling [61,204,205]. Mutational frequencies for genes encoding proteins involved in the NF-κB pathway are as follows: *TNFAIP3* (9–18%), *TBL1XR1* (7–13%), *KLHL6* (9–10%), *NFKBIE* (3–8%), *ZC3H12A* (3–7%), and *NFKBIA* (5%) [206]. Rare germline polymorphisms enriched in ABC-DLBCL patients were detected, which enable polyubiquitin-dependent NF-κB activation [56]. While mutations affecting genes coding for NF-κB proteins are relatively rare in DLBCL, erroneous NF-κB activation is often observed due to other oncogenic events affecting the upstream signaling network. The latter culminates in enhanced activity of NF-κB [69,70,205]. Numerous signaling pathways converge on NF-κB regulators, providing ample means by which tumors can aberrantly stimulate NF-κB [205]. The oncogenic role of the aberrant NF-κB network in DLBCL and mutations hitting the players of the NF-κB pathway or their upstream partners have been covered in the literature, e.g., [204,207,208].

To summarize, the constitutive activation of NF-κB is a hallmark of ABC-DLBCL. NF-κB-activating mutations in DLBCLs predominantly involve the canonical NF-κB pathway. The constitutive activation of the canonical NF-κB pathway facilitates ABC-DLBCL via the inactivation of BLIMP1. An important target of NF-κB in ABC-DLBCL is IRF4, an essential TF that drives plasmacytic differentiation. NF-κB signaling in ABC-DLBCL cells induces the elevated production of the cytokines IL6 and IL10, which in turn, through an autocrine loop, activate another TF, STAT3. The simultaneous occurrence of *CARD11* and *CD79* mutations, when it happens, could account for the NF-κB activation in roughly one-third of ABC-DLBCLs. The list of genetic alterations affecting NF-κB in the ABC-DLBCL may be longer [205]. There is still room for in-depth molecular investigations of mechanisms affecting canonical and non-canonical NF-κB signaling in ABC-DLBCL, which could give birth to new ideas for precision medicines.

Lenalidomide alters the signaling via the NF-κB pathway owing to targeting Cereblon, a component of the E3 ubiquitin ligase multiprotein complex. Primarily, lenalidomide helps drag proteins to an E3 ubiquitin ligase for subsequent proteasomal degradation; hence, it acts as small-molecule protein degrader. Lenalidomide, sold as Revlimid, is an oral immunomodulatory imide anti-neoplastic agent with a diverse spectrum of therapeutic effects, even beyond anti-tumor activity, such as immune modulation and anti-angiogenic actions [209]. The tumoricidal action of lenalidomide is related to the downregulation of IRF4 protein downstream of Cereblon, the lenalidomide target. Furthermore, when the production of IRF4 diminishes, downregulation of BCR-induced NF-κB signaling follows [210]. In addition, lenalidomide stimulates the proliferation and activation of NK cells, thereby enhancing NK cell-mediated cytotoxicity and ADCC. These effects appear secondary to cytokine production from T-cells [209,211]. Lenalidomide exhibited enviable activity in R/R DLBCL patients, alone or in combinatorial regimens [212,213,214,215,216,217,218]. The drug exerted the most significant effect on non-GCB-DLBCL, as repeatedly reported [212,215]. Adding lenalidomide to GCB-DLBCL cell line (OCI-Ly1) suspensions or the patient-derived mouse xenograft model (CY-DLBCL), both resistant to third-generation CD19 CAR T-cells (CARTs), enabled the cells to overcome the resistance to CARTs. The regimes combining different CARTs with lenalidomide diminished the tumor burden in various DLBCL mouse models [219].

### 9.4. Targeting Spleen Tyrosine Kinase and Protein Kinase C

From a clinical perspective, besides BTK, but still within the BCR signaling cascade, two other targets seem to be druggable by current anti-neoplastic agents, SYK and protein kinase C (PKC)β [54]. SYK, a cytosolic non-receptor protein tyrosine kinase, represents an attractive drug target in neoplasms [220,221]. The oral SYK inhibitor (SYKi) fostamatinib had reached a phase II clinical study that recruited 68 R/R DLBCL patients; albeit, while the drug was generally well tolerated, its efficacy at the applied doses and schedule was poor (Table 3). Of note, none of the patients who benefited from the therapy had an ABC genotype [222]. The results of the phase II trial of the oral, selective SYKi entospletinib (GS-9973) in 43 patients with R/R DLBCL were poor since not a single patient achieved a CR or partial response and 60% had progressive diseases [223]. In a recent phase I study, a dual SYKi/FMS-like tyrosine kinase-3 inhibitor, mivavotinib, was administered to 89 R/R DLBCL patients; the observed responses were deep and durable [224]. While SYK appears to be a desirable candidate for treating R/R DLBCL patients, more research is needed. Enzastaurin is an oral drug initially developed to treat solid and hematological malignancies, an isozyme-specific PKCβi. PKCβ is involved in both the Akt and MAPK signaling that is augmented in many malignancies. Although enzastaurin had desirable pre-clinical results, preventing angiogenesis, inhibiting the proliferation, and inducing apoptosis, all accompanied by limited cytotoxicity, when translated into phase I clinical trials, it failed to unleash its full potential [225]. There have been more clinical trials of PKCi in tumors, but none have demonstrated significant clinical benefits. As proposed, there may be a limitation to designing an anti-tumor therapeutic strategy by targeting PKC alone [226].

### 9.5. Notch Signaling in Diffuse Large B-Cell Lymphoma

Notch signaling is an evolutionarily conserved pathway [227]. The Notch receptor and its ligands are transmembrane proteins containing EGF-like repeat sequences. The Notch receptor–ligand interaction initiates a classical Notch signaling cascade [228]. *NOTCH2* codes for the large Notch2 protein with 2471 amino acids [229]. Although structurally similar to Notch1, Notch2 signals are not as strong as Notch1 signals. In addition, Notch2 displays unique functions during the evolution of T- and B-cells [230,231]. Notch receptors are activated through concerted proteolytical cleavages at three specific sites within certain functional domains of the Notch, thus releasing one intracellular fragment, which moves to the nucleus, where it regulates specific gene expression [232]. Notch receptors are involved in a plethora of processes that link the fate of one cell to the fate of its neighbor cells via physical interactions of Notch receptors with their cognate ligands expressed on the adjacent cells [227,228]. Notch1 and Notch2 are expressed on the surface of mature B-cells, whereas the cognate ligands are expressed by neighbor stromal cells. Importantly, in DLBCL, constitutive Notch activation is rare despite the possible *NOTCH* mutations. The differences between *NOTCH1*-mutated and *NOTCH2*-mutated DLBCL tumors cannot be explained solely by the intrinsic differences between activated Notch1 and Notch2. There have to be other factors that contribute to this distinction, most likely the differences in the TME [233]. The pieces of the Notch puzzle in B-cell lymphoma are slowly appearing in the literature; for example, in DLBCL, Notch signaling mediates M2 polarization of tumor-associated macrophages (TAMs) through the CREBBP/EP300-FBXW7-Notch-CCL2/CSF1 pathway [122]. Then, lymphoma cells that mediate the Notch1 signaling in bone marrow-derived macrophages facilitate the activation of STAT3 and STAT6 in the same macrophages. Many more experiments should be conducted to dissect the mechanisms of the Notch1/IRE1/XBP1s signaling pathway in DLBCL development and immune escape [234]. The potential therapeutic strategy to indirectly target Notch is doing so at the posttranscriptional level, e.g., by interfering with the cleavage-mediated maturation of Notch2 [232]. The response of B-cell neoplasms to monotherapy exerted by a Notch pathway inhibitor was restricted to tumors with elevated levels of nuclear Notch. Therefore, we do not expect *NOTCH2*-mutated DLBCLs to respond to Notch-directed therapeutics. In contrast, Notch inhibitors may have a say in *NOTCH1*-mutated DLBCL, particularly in combination with other agents [233]. However, further studies will address these issues.

### 9.6. Targeting BCL2, BCL6, and MYC Proteins in Diffuse Large B-Cell Lymphoma

The genetic alterations that affect *BCL2* are of great importance in DLBCL, influencing the clinical outcome. Venetoclax is an SMI of BCL2 (BCL2i) and is the only FDA-approved anti-tumor drug developed against a BCL2 family member [235]. Venetoclax is utilized for treating AML [236] and CLL [237]. A novel investigation of the combined efficacy of venetoclax plus R-CHOP in patients with DLBCL overexpressing both MYC and BCL2 proteins could not detect prolonged PFS in comparison to R-CHOP alone, but instead resulted in higher toxicity [238]. A novel candidate for targeting surfaced upon the detection of robust glutaminase-1 (GLS1) expression in DLBCL biopsy samples and cell lines. It was proposed that GLS1i, such as CB-839, may enhance the anti-neoplastic activity of venetoclax by promoting oxidative stress [239].

BCL6 is a TF whose expression in DLBCL is partly maintained due to chromosomal translocations, but most DLBCLs express BCL6 regardless of genetic lesions. For lymphoma cells, including DLBCL, BCL6 is a lineage factor, which controls B-cell differentiation, migration (via S1PR), DNA damage response, the cell cycle, and cell death. Activating mutations of *BCL6* or inactivating mutations targeting BCL6 protein inhibitors (*EP300*, *CREBBP*) will cause suppression of these processes [206]. An innovative workflow combined with the structure-based drug design was applied to discover novel SMIs that could disrupt the BCL6–corepressor interaction, which led to several novel BCL6i [240]. In another study, a small-molecule BCL6i, WK500B, was developed, which blocked BCL6 repression complexes, reactivated BCL6 target genes, killed DLBCL cells, and caused apoptosis and cell cycle arrest (Table 3). In animal models, WK500B inhibited GC formation and DLBCL tumor growth without toxic side effects; hence, it may be a promising (oral) therapeutic agent for DLBCL [241].

MYC proteins, encoded by the *MYC* gene family, are powerful TFs that activate the expression of many pro-proliferative genes and may trigger oncogenesis. Protein c-Myc has been implicated in around 40% of malignancies, including B-cell lymphomas and DLBCL. The clinical significance of MYC in DLBCL has been investigated for a long time and reviewed elsewhere [242]. Genetic alterations of *MYC*, overexpression, translocations, mutations, and increased copy number in DLBCL were comprehensively summarized [243]. *MYC* mutations were observed in 750 dnDLBCL patients by Sanger sequencing. Specifically, 33.3% of these patients carried *MYC* mutations in their DNA, while another 16.1% had it at the protein level (nonsynonymous mutations). Most *MYC* mutations are not driver mutations but rather passenger mutations taking place during the genesis and development of DLBCL [244]. Despite being investigated for decades, MYC proteins seem “undruggable”. However, there may be indirect ways to tackle MYC, one of which is through the BET family, for which assorted compounds have been developed. BET proteins are epigenetic readers that enhance *MYC* transcription by binding to the acetylated histones; hence, one could inhibit MYC actions by blocking the complex formation between a BET and acetylated histone, e.g., by administering a BET inhibitor (BETi). While tackling overactive MYC via BETi seems attractive, most BETi are pan-BETi, even if they manifest some preferences for binding specific BET proteins [245]. Table 3 summarizes the major pathways affected by DLBCL along with the established or potential therapeutics that target the pathways.

### 9.7. BET Degraders and PROTACs in Diffuse Large B-Cell Lymphoma

Nonetheless, there is still hope in targeting BET as an anti-neoplastic strategy because of encouraging pre-clinical and early clinical evidence of the cytotoxicity of BET degraders in DLBCL [246]. BET degraders are chimeras containing a BETi moiety (which allows binding to BETs) and an additional small molecule that recruits an E3 ubiquitin ligase complex, thus marking BET for ubiquitination and then for proteasomal degradation. BETi and the small molecule similarly hijack the ubiquitin machinery that degrades target protein(s) [247]. The linker connects BETi and a small-molecule agent. Another popular term for these heterobifunctional degraders is proteolysis-targeting chimeras (PROTACs), which have been extensively reviewed, e.g., [248,249,250,251,252,253].

A parallel study of the BET degrader MZ1 and pan-BETi birabresib activities in seven established ABC-DLBCL cell lines and mouse xenograft models demonstrated MZ1-induced cell death, which was accompanied by the drug’s broad effects on the transcriptome of ABC-DLBCL cells. The study justified the further development of BET degraders in DLBCL. Moreover, regarding efficacy in killing DLBCL cells in vitro, MZ1 induced cell death in all the ABC-DLBCL cell lines, while the BETi was cytotoxic only in a subset. Of note, the MZ1 effect overlapped with that induced by cyclin-dependent kinase 9 (CDK9) inhibition [246].

Whilst many human E3 ligases exist, PROTACs are chiefly constructed to hit Cereblon and VHL [253]. PROTACs can be highly efficient as anti-neoplastic drugs, but they suffer a few limitations, e.g., their large size. The large-sized drugs are very often difficult to transport into the cells. Smaller molecules that induce targeted protein degradation are desirable alternatives to PROTACs. Small molecules have already been created for this purpose and act via different modus operandi: some recruit an E3 ligase to their target protein(s) (molecular glues) while others introduce the hydrophobic areas into targets or cause local unfolding of the targets, and all these events trigger degradation [254]. The history of developing protein degraders, from thalidomide to the latest PROTACs and related technologies, has been recently published [255]. Wang and co-authors portrayed new-generation PROTACs, including small-molecule PROTAC prodrugs, conjugates of biomacromolecules and PROTACs, and nano-PROTACs, all designed to improve the anti-tumor actions of PROTACs or alleviate the undesirable physicochemical characteristics inherent in the first-generation PROTACs, thereby reducing off-target hits [256].

Protein degraders finally entered clinical testing in 2019 after being scrutinized in pre-clinical models, and over 18 protein degraders are currently in phase I or phase I/II clinical trials, having been administered to patients with various tumor types. We shall not mention every one of these novel potential drugs because they have been described in detail recently [257]. Several of them have been investigated in DLBCL cell lines and models: Nx-2127, a BTK degrader, blocks the proliferation of ibrutinib-resistant DLBCL cells in vitro [258]; Nx-5948 selectively breaks down BTK, which was confirmed by proteomics in DLBCL cells [259]; and KT-413 is a protein degrader that hits IL1 receptor-associated kinase 4 and displays preference towards *MYD88*-mutant DLBCL models [260]. The newest survey elaborated on PROTACs in the clinical context of hematological malignancies [261]. Targeted degradation of BTK, a crucial target in R/R B-cell lymphomas, by PROTACs, has been explored in three completed clinical trials, which were presented at the American Society of Hematology Meeting in 2023 [262]. These three, BGB-16673 [263], Nx-5948 [264], and Nx-2127 [265], were tested in R/R DLBCL patients.

### 9.8. Targeting Mammalian/Mechanistic Target of Rapamycin (mTOR) in DLBCL

Mammalian/mechanistic target of rapamycin, mTOR, is an *MTOR*-encoded member of the PI3K-related kinase family. mTOR is a hub of mTOR complex 1 (mTORC1) and mTORC2, which manage distinct cellular processes [266]. The multifaceted role of mTOR signaling in human health and disease has been reviewed elsewhere [267]. mTORC1 is nutrient sensitive, i.e., it serves as a central hub for nutrient signaling and, hence, cell growth. Proteins operating as amino acid sensors can directly bind to amino acids and convey the information on their levels to mTORC1, e.g., SLC38A9 is a lysosomal arginine sensor and sestrin is a cytosolic leucine sensor, to name a few. mTORC1 plays a vital role in cellular catabolic programs, e.g., it can directly modulate autophagic machinery. PIK3C3, required for autophagosome nucleation and maturation, is also a mTORC1 target [266]. mTORC1 is a downstream effector of the PI3K/Akt oncogenic signaling pathway and is frequently activated in diverse malignancies. The most recent article elaborated on the mTOR pathway from a novel perspective with new conclusions [268]. The biological role of mTOR in B-cell lymphomas, anti-neoplastic agents that target the mTOR pathway, and the respective therapeutic context have also been reviewed [269].

### 9.9. Targeting Phosphatidylinositol-3-Kinase in DLBCL

PI3Ks come from a family of lipid kinases that can be classified according to their specificity and structure. p110δ and p110γ are restricted to hematopoietic cells. The activation of PI3K-dependent pathways represents a crucial event of BCR/pre-BCR signaling [270]. The primary function of PI3K is the phosphorylation of phosphoinositide PIP2 into PIP3. PTEN reverses the PI3K activity by dephosphorylating PIP3 back to PIP2. Mutations or deletions resulting in the loss of PTEN are observed in DLBCL relatively often [271], and we are now aware of PTEN’s roles in hematopoiesis and lymphoma development. PI3K/Akt/mTOR pathway activation in lymphomas is associated with the enhanced expression of various cytokines and growth factors, such as IL6, IL10, and the platelet-derived growth factor [269]. *PIK3CA* mutations, including amplification, were found in 8% of DLBCLs, chiefly affecting the catalytic domain [272]. PI3K/Akt/mTOR is a commonly active pathway in DLBCL. When in a state of chronic BCR or PTEN deficiency, DLBCL may turn to constitutive activation of PI3K [273]. We have recently reported how female DLBCL patients manifested significant alterations in plasma levels of phosphatidylinositols (PIs), which could be explained by the depletion of plasma depots of PI34:1 to produce second messengers of the PI3K pathway [274]. Since PI3Kδ is expressed predominantly in leukocytes and overexpressed in B-cell malignancies, where it is involved in BCR signaling, PI3Kδ has been investigated as a candidate for targeted lymphoma therapy. In DLBCL, selective PI3Kδis were inefficient when used as a monotherapy [275]. Idelalisib, the first PI3Kδ-selective inhibitor, did not produce a desirable response in R/R DLBCL patients [276]; duvelisib, a novel dual inhibitor of PI3Kδγ, showed clinical activity in advanced cases of indolent NHL [277]. Since pan-PI3Ki may cause severe side effects, the anti-lymphoma efficacy of the specific PI3Kβ/δ inhibitor AZD8186 was explored in a subset of DLBCL models. On the molecular level, PI3Kβ/δ inhibition decreased pro-survival NF-κB activity or led to the downregulation of the oncogenic MYC [273]. Bayer’s Copanlisib, targeting PI3Kα and PI3Kδ and approved for recurrent FL in 2017, is being tested in several phase II clinical trials, but in combination with nivolumab, R-CHOP, bendamustine + RTX, or venetoclax [278].

### 9.10. Targeting p53 Tumor Suppressor in DLBCL

*TP53* encodes a famous tumor-suppressor protein, p53, which plays a critical role in activating genes that regulate cell death and cell cycle progression [54,206]. Protein p53 triggers apoptosis in cells with damaged DNA. Virtually all tumors inactivate p53 using diverse strategies, e.g., by mutations/deletions of *TP53* or by amplifying negative regulators of p53 activity. p53 is inactivated in 8–24% of DLBCLs owing to *TP53* deletion. *TP53* mutations mark poor prognosis in DLBCLs and can be associated with therapeutic resistance [279]. In addition, even if *TP53* mutations were absent at the time of diagnosis, DLBCL patients often presented with *TP53* alterations when relapsing following R-CHOP therapy, which implied that *TP53* is prone to mutations under immuno-chemotherapy [280]. Fortunately, *TP53* mutation status does not influence outcomes in DLBCL patients, at least not in those treated with CARTs [281]. The p53 pathway in lymphoma may be indirectly blocked by other mechanisms. For example, in a healthy state, tumor suppressor p14ARF, encoded by *CDKN2A*, inhibits the murine double minute 2 (*MDM2*) oncogene so MDM2 would not inactivate p53. In other words, p14ARF interferes with the MDM2-mediated ubiquitination and degradation of p53 by sequestering MDM2 in the nucleolus and preventing MDM2–p53 interaction and nuclear export of p53. MDM2 is an E3 ligase that breaks down NF-κB, HIF-1, FOXO3a, and others. However, in the case of mutations that inactivate *p14ARF*, p53 will be constitutively blocked by MDM2. The mutations that activate *MDM2* lead to p53 inactivation, which, in turn, causes the failure of apoptotic pathways [54]. Not only is MDM2 the best-documented negative regulator of p53 but it also has p53-independent activities, which is a rationale for developing MDM2-based anti-neoplastic agents [282]. The only MDM2 antagonist that has entered phase III clinical trials so far is idasanutlin, which first demonstrated remarkable anti-DLBCL activity as a single agent in the pre-clinical setting. The modus operandi of idasanutlin involved decreasing mitochondrial membrane potential and levels of MDM2 and XIAP proteins. Moreover, idasanutlin augmented the anti-tumor actions of other SMIs [283]. Whilst the combination of idasanutlin and venetoclax with an anti-CD20 mAb obinutuzumab seemed feasible and promising, there have been other options in the field, making this combination redundant [284].

### 9.11. JAK/STAT (Signal Transducer and Activator of Transcription) Signaling in DLBCL

The JAK/STAT (Signal transducer and activator of transcription) signaling pathway operates downstream of cytokines, including IL4 and IL21, which work in synergy to sustain elevated BCL6 expression. The concomitant loss of IL4 and IL21 signaling wholly abolishes the GC response [285]. Apart from that, IL4 can upregulate CD79A/B expression and strengthen BCR signaling via STAT6, making the STAT6 protein an important participant in the GC reaction [47]. Half of all EZB DLBCL patients carry either activating mutations/amplification of *STAT6* or deletions of genes encoding suppressors of STAT6, as is SOCS1 [56]. So, the constitutive activation of STAT6-sent signals facilitates the GC response, preventing apoptosis and favoring lymphoma survival [47]. The most novel finding is that IL4-induced signaling may directly downregulate the BCL6 protein, thus releasing B-cells from the GC program and promoting the formation of memory B-cells [286]. Also, *STAT6*^D419^ mutations are enriched in R/R DLBCL samples, as per a recent report, which implied the involvement of JAK/STAT signaling in therapeutic resistance [287]. Recurrent mutations activating the oncogenic JAK-STAT (and Notch) pathways and frequent amplifications of 9p24.1 contributing to immune escape via PD-L1 overexpression were described in EBV+ DLBCL patients. These findings may further propel the exploration of therapeutic potential in targeting such aberrations in patients with EBV+ DLBCL [22].

CDK9 inhibitors, CDK9is, are relatively new drugs that inhibit the transcription of anti-apoptotic and pro-survival proteins, such as MYC, MCL1, and cyclin D1. The activity of CDK9is and their synergism with other anti-neoplastic drugs were investigated in animal tumor models. In the first-in-human trial of enitociclib monotherapy on a small cohort of DH/TH HGBCL patients, who are difficult to treat, the CR was achieved in two of seven subjects. The data implied the probable use of CDK9is as a combination partner with other therapeutic approaches for hematological malignancies [288].

**Table 3:** Representative therapeutics targeting major signaling pathways in DLBCL.

Major Pathway Affected		Drug Name/Designation	Drug Features	Modus Operandi	Refs.
BCR signaling	BTKi (1st gen.)	Ibrutinib *	high response rate in ABC; excellent RTT in ABC subsets with tandem *CD79B* & *MYD88*^L265P^ mutations; drug not selective	covalent attachment to Cys481 in ATP-binding pocket of BTK	[191]
	BTKi (2nd gen.)	tirabrutinib	targets only B-cells; targets off-target kinases: TEC, BMX, HUNK, RIPK2		[197]
		acalabrutinib	33% ORR in R/R ABC vs. 16.7% in R/R GCB/UC		[191,198]
		TL-895 (formerly M7583)	interferes with the growth of a subset of ABC cell lines & 5/21 xenograft models	potent, highly selective, ATP-competitive, irreversible BTKi	[200]
		orelabrutinib	approved for MCL, CLL/SLL	irreversible highly selective BTKi	Ref. in [203]
	SYKi	fostamatinib	poor efficacy in phase II trial on 68 R/R DLBCL patients	binds to the ATP binding pocket of SYK and inhibits its kinase activity as an ATP-competitive inhibitor; blocks BCR-mediated activation of B-lymphocytes	[221,222]
		entospletinib (GS-9973)	poor results in phase II trial on 43 R/R DLBCL patients	selective, reversible, ATP-competitive SYKi, blocks BCR signaling & proliferation in B-lymphocytes	[223]
	PKCβi	enzastaurin	desirable pre-clinical results but not so effective in phase I trials	isozyme-specific PKCβi involved in AKT & MAPK pathways	[225,226]
Notch signaling	potential Notch2i	not known	NOTCH2 activation is rare, even in *NOTCH2*-mutated *DLBCLs*	*NOTCH2*-mutated *DLBCLs* unlikely to be responsive to Notch-directed therapeutics	[233]
	Potential Notch1i	under investigation	Notch target gene expression is elevated in *NOTCH1*-mutated *DLBCLs*	Notch1i combined with other agents may be effective in *NOTCH-1* mutated *DLBCLs*	[233]
NF-κB signaling		lenalidomide (Revlimid)	downregulation of IRF4 protein downstream of Cereblon; downregulation of BCR-induced NF-κB signaling; the most significant effect in non-GCB *DLBCLs*	targets Cereblon-substrate recognition protein in E3 ligase ubiquitin complex	[209,210]
PI3K	PI3Kδi	idelalisib	did not produce desirable response in R/R DLBCL patients	isoenzyme-specific inhibitor of PI3 kinase δ	[276]
	Dual PI3Kδi & PI3Kγi	duvelisib	dual inhibitor shows clinical activity in advanced cases of indolent NHL		[277]
	PI3Kβ/δi	AZD8186	PI3Kβ/δ inhibition decreased the pro-survival NF-κB activity or led to downregulation of the oncogenic MYC		[273]
	pan-PI3Ki	copanlisib	approved for recurrent FL after two lines of therapy; limited but encouraging data in clinical trials	inhibits all four PI3K isoforms (P110α, P110β, P110δ, and P110γ), but the highest selectivity for the PI3Kα and PI3Kδ	[278]
BCL2	BCL2i	venetoclax	utilized for treating AML & CLL	Venetoclax is a BH3-mimetic that helps restore the process of apoptosis by binding directly to the BCL2 protein	[236,237]
Glutaminase (GLS)1	GLS1i	CB-839	cytotoxicity of CB-839 largely mediated by ROS induction, and not by a decreased energy metabolism or restricted supply of amino acids and nucleotides		[239]
MYC	MYC protein inhibitors MYC proteins investigated for decades, but seem ‘undruggable’				[242,243,244]
BCL6	BCL6i	9 novel structures of SMIs revealed by multiple biophysical techniques & X-ray crystallography	SMIs able to disrupt protein-protein interactions between BCL6 and its co-repressor proteins (BCOR, NCOR, SMRT)		[240]
		WK500B	a synthetic small-molecule compound, directly binds to BCL6 BTB domain, significantly inhibiting the BCL6/SMRT interaction, reactivating BCL6 target genes & killing DLBCL cell lines via cell cycle arrest and apoptosis		[241]
p53 tumor suppressor	MDM2 antagonist	idasanutlin	MDM2 is negative regulator of p53; entered Phase III clinical trials	blocks the MDM2 protein-XIAP RNA interaction, led to both MDM2 and XIAP degradation, and induced apoptotic death in DLBCL	[283]
transcription inhibitor	CDK9i	enitociclib	clinical activity in a small cohort of patients with high grade DH/TH BCL inducing complete responses in 2 of 7 subjects	Inhibits transcription of anti-apoptotic and pro-survival proteins MYC and MCL1	[288]
PI3K/Akt/mTOR	HDACi + BTKi	chidamide + orelabrutinib	Synergistically inhibited the expression of PI3K (p85), AKT, and mTOR proteins; regulate BCL2 & caspase 3, inducing apoptosis of DLBCL cells		[203]
combinatorial therapy	BCL2i + standard-of-care anti-DLBCL regimen	venetoclax + R-CHOP	In DLBCLs overexpressing MYC and BCL2 (double expressors) the combination did not improve PFS, but resulted in higher toxicity		[238]
	GLS1i + BCL2i	CB-839 + ABT922	deep and durable responses in phase I trial on 89 R/R DLBCL patients	the first evidence that DLBCL cells are dependent on glutaminolysis; the combination promotes ROS formation and cytotoxicity in DLBCL cells	[239]

Abbreviations: BCR, B-cell receptor; BTK, Bruton’s tyrosine kinase; BTKi, BTK inhibitor; SYK, spleen tyrosine kinase; ABC, activated B-cell-like DLBCL; ORR, overall response rate; RTT, response to therapy; gen., generation; UC, unclassified; MCL, mantle cell lymphoma; CLL/SLL; chronic lymphoid leukemia/small lymphocytic lymphoma; FL, follicular lymphoma; PK, protein kinase; TEC, tyrosine protein kinase encoded by TEC; BMX, bone marrow kinase on chromosome X; HUNK, hormonally upregulated neu-associated kinase; RIPK2, receptor-interacting protein kinase 2; GLS, glutaminase; PI3K, phosphatidylinositol-3 kinase; MDM2, murine double-minute 2 protein; CDK9, cyclin-dependent kinase 9; HDAC, histone deacetylase; PFS, progression-free survival; BCL2, B-cell lymphoma protein 2; mTOR, mechanistic target of rapamycin; AKT, protein kinase B; R/R, relapsed or refractory disease; ROS; reactive oxygen species. * approved therapeutics.

### 9.12. Targeting Immune Evasion Mechanisms and N-Glycosylation in DLBCL

Upon transformation, aberrant B-cells may express non-self-antigens or neo-antigens that would make them visible to immune effector cells: T-cells, NK cells, and macrophages able to kill the aberrant B-cells. In order to escape immune surveillance, oncogenic B-cells may try a few strategies: hide from the immune cells (ESCAPE), actively suppress the function of the immune system (INHIBITION), and even modify the immune response (MODIFICATION) to support their own goals [289,290,291]. One prominent hallmark of neoplastic cells is the loss of the antigen presentation capacity, which promotes tumor progression and resistance to immunotherapy. Fifty percent of DLBCLs evade immune surveillance via somatic genetic lesions that lead to the lack of the cell-surface expression of the MHC-I, thus preventing the presentation of tumor neo-antigens to the immune system [292]. Across B-cell lymphomas, loss of MHC-I, but not MHC-II, is preferentially restricted to DLBCL [293]. Despite expressing MHC-I, 40% of DLBCL cases carry monoallelic *HLA-I* genetic alterations that limit the repertoire of neo-antigens for presentation to immune cells. The novel results also suggest a multistep process of HLA-I loss in DLBCL development, including both germline and somatic events, and have direct implications for pathogenesis [292]. Henceforth, the immunotherapeutic targeting of DLBCL must include agents that would prevent or mitigate immune evasion.

The immune system is strictly regulated by glycosylation by attaching highly diverse and dynamically changing oligosaccharide structures (glycans) to almost every immune cell receptor. The B-cell surface is enriched with glycosylated proteins (mostly N-glycosylated) and saccharide-binding proteins (lectins). The latter exhibit specificity towards glycans ubiquitously residing on cell surfaces. N-glycans are pivotal for the development, selection, and maturation of B-cells. An exemplary role of N-glycosylation in B-cell life is the adjustment of events during B-cell maturation and pre-BCR folding, the BCR-triggered signal transduction cascade, and many others, including those that go via the CD22 inhibitory co-receptor and Siglec-G. The altered profile of IgG-bound glycans aids disease progression and remission and is sensitive to applying therapeutic substances and immunosuppressive agents. N-glycans’ roles in B-cell biology have been elaborated elsewhere [294]. Without an intent to dive into the ocean of N-glycome in hematological malignancies, we shall mention a new article here, which reported how administering a hexose sugar, fucose, to mice with melanoma enhanced the efficacy of PD-1 inhibition. Dietary fucose ended up in N-glycans attached to surface protein HLA-DRB1, which engages in antigen presentation, promoting T-cell-mediated anti-melanoma immunity and facilitating efficacy of the immunotherapy [295]. Herein, we take an opportunity to announce the advent of glycomics, both N-glycomics and O-glycomics, particularly in the context of glycans attached to surface antigens and immune receptors of immune cells, APCs, and diverse cell subsets in the TME.

## 10. Programmed or Regulated Cell Death in B-Cell NHL and DLBCL

The term PCD defines physiological death during specific developmental stages and normal tissue turnover, whereas regulated cell death (RCD) is an umbrella term for the diverse cell deaths induced by extensive intracellular or extracellular perturbations. Specific molecules mediate PCD and RCD following a precise sequence of events [296]. While cell death by accident is an uncontrolled biochemical process, PCD and RCD involve intricate regulations and diverse mechanisms [297]. Cell death patterns and PCD-related gene signatures can be leveraged for tumor prognosis and targeted therapies [298]. The idea is to compile all the genes known to be involved in regulating 10–16 PCD patterns (apoptosis, necroptosis, pyroptosis, ferroptosis, cuproptosis, reticulocyte death, netotic cell death, entotic cell death, lysosome-dependent cell death, parthanatos, autophagy-dependent cell death, oxeiptosis, alkaliptosis, and disulfidoptosis) and screen for genes associated with DLBCL prognosis, employing Cox regression. Transcriptome data for multiple DLBCL datasets were retrieved from GEO, while knowledge on genes involved in different PCDs was extracted from the relevant literature on DLBCL; a prognostic nomogram model was constructed based on prognosis-related genes, and the AUC of 1-, 3-, and 5-year survival predicted by this model reached 0.790, 0.816, and 0.825, respectively [299].

### 10.1. Autophagy

**Figure 2:**
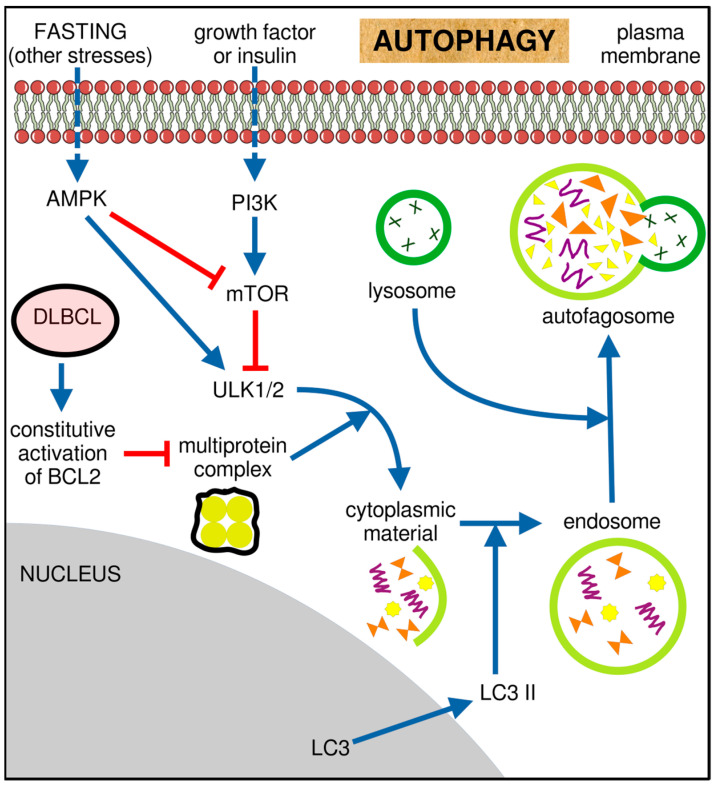
Major processes governing autophagy. AMPK, 5′-adenosine monophosphate-activated protein kinase; PI3K, phosphatidyl inositol-3 kinase; mTOR, mechanistic target of rapamycin; ULK1/2, Unc-51-like autophagy-activating Kinase 1/2; LC3, microtubule-associated protein 1A/1B-light chain 3. red arrow-inhibition.

The primary role of macroautophagy (or just autophagy) is to sustain cellular homeostasis, which is doable via degrading damaged organelles. The suppression of PI3K/Akt/mTORC1 initiates autophagy that starts with the phosphorylation of unc-51-like kinase-1 (ULK1). The ULK1 is critical for mobilizing microtubule-associated protein 1 light chain-3 (LC3) in autophagosome formation (Figure 2). Atypical autophagy facilitates metabolic adaptation in neoplastic cells. ULK1 is a druggable serine/threonine kinase [300]. Interesting novel work revealed the preferential targeting of ULK1 inhibition in GCB-DLBCL over ABC-DLBCL, thus justifying a potential subtype-specific therapeutic approach [301]. Usually, the high gene and protein levels of histone H3 methyltransferase (H3MT) G9a in DLBCL can be reduced by niclosamide. The drug was shown to suppress the growth of DLBCL cell lines by inducing autophagy [302]. This sort of drug opens yet another venue for targeted therapy of DLBCL. Niclosamide has been a proven drug for treating tapeworm infection since 1982 [303]. However, there are also many non-histone targets of G9a, including TFs, chromatin remodeling factors, DNMT1, and DNMT3 [304], which could mean many off-target hits, severe AE, and drug-induced toxicities. Mechanisms underlying autophagy are displayed in Figure 2.

### 10.2. Cuproptosis

Glutathione peroxidases (GPXs) are enzymes that protect against oxidative damage, i.e., antioxidant enzymes, which remove reactive oxygen species (ROS) by oxidizing glutathione (GSH) to glutathione disulfide (GS-SG) while simultaneously reducing peroxide to water. It is well-known that the ratio of GSH/GSSG within cells reflects the extent of cellular oxidative stress. Glutamate–cysteine ligase and glutathione synthase (GS) are involved in GSH synthesis, and both are regulated by TFs, including NF-κB [305].

**Figure 3:**
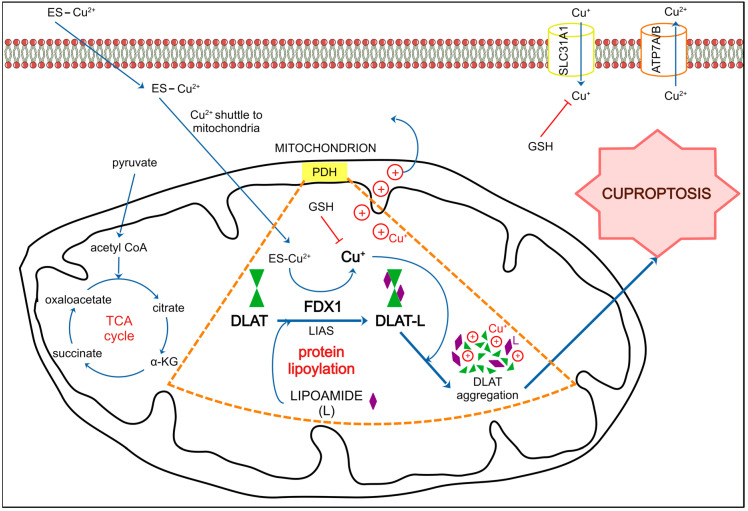
Major processes behind cuproptosis. ES, elesclomol; SLC31AA1, solute carrier membrane protein 31A1; ATP7A/B, copper-transporting P-type ATPase; PDH, pyruvate dehydrogenase complex; TCA, tricarboxylic acid; GSH, glutathione; DLAT, component of the PDH complex; FDX1, ferredoxin 1; LIAS, lipoic acid synthetase; αKG, α-ketoglutarate. red arrow-inhibition.

Copper and iron (in their ionic forms) may bring about intracellular oxidative stress by oxidizing GSH (in the Fenton reaction, no GPX needed), while producing hydroxyl radicals, dangerous ROS that can cause damage to DNA and other biomolecules [306]. The significance of GPX4 in the diagnosis, treatment, and pathobiology landscape of DLBCL has been recently described. Data on GPX4 expression levels in DLBCL, including tumor tissues, peripheral blood, and single cells, were retrieved from several databases, e.g., GEO, and correlation analysis was performed that resulted in the following findings: high expression of GPX can inhibit DLBCL cell proliferation; negative correlation exists between GPX expression and 16 core pathogenic genes; and GPX4 is a candidate biomarker that deserves further exploration in DLBCL [307]. Although it is an essential enzyme cofactor, copper ion becomes toxic in concentrations beyond a certain threshold, which is maintained by evolutionarily conserved mechanisms. Since its discovery in 2022, a novel PCD, copper-induced cell death or cuprotosis, has been a research hotspot. Cuproptosis occurs via the direct binding of copper ions to lipoylated proteins operating within the tricarboxylic acid (TCA) cycle, which causes lipoylated proteins to aggregate (Figure 3), leading to proteotoxic stress and, ultimately, cell death [308]. We propose that nature itself designed this particular PCD type to fit this purpose. We base our hypothesis on the scarcity of protein lipoylation. It is a rare but highly conserved post-translational modification arising via the covalent linking of lipoamide (organosulfurous compound) to the Lys residue in only four multimeric metabolic enzymes in mammals. Yet, these four lie at the heart of the metabolic universe. They maintain proper mitochondrial life functioning [309]. The cellular metabolism of copper, detailed mechanics of cuproptosis, and copper-related tumor signaling pathways have been comprehensively covered elsewhere [310,311], although the authors only elaborated on the involvement of cuproptosis in solid cancers. Zhang et al. (2023) retrieved, from the GEO database, transcriptome data on twelve genes pertaining to cuproptosis, which were discovered in another study [308], and re-used them to create a nomogram that displayed more accuracy in predicting OS in DLBCL patients than the IPI [312]. The cuproptotic events in DLBCL can be explored through other lenses, such as cuproptosis-related lncRNAs, which can then be incorporated into an innovative risk model for DLBCL. The prognostic index based on cuproptosis-related lncRNA could accurately predict the outcome for DLBCL patients, regardless of the clinicopathological heterogeneity. Finally, the high-risk group was more sensitive to vinorelbine, an anti-neoplastic chemotherapeutic that induces cell cycle arrest in the G2-M phase and can replace vincristine in CHOP [313].

### 10.3. Ferroptosis

**Figure 4:**
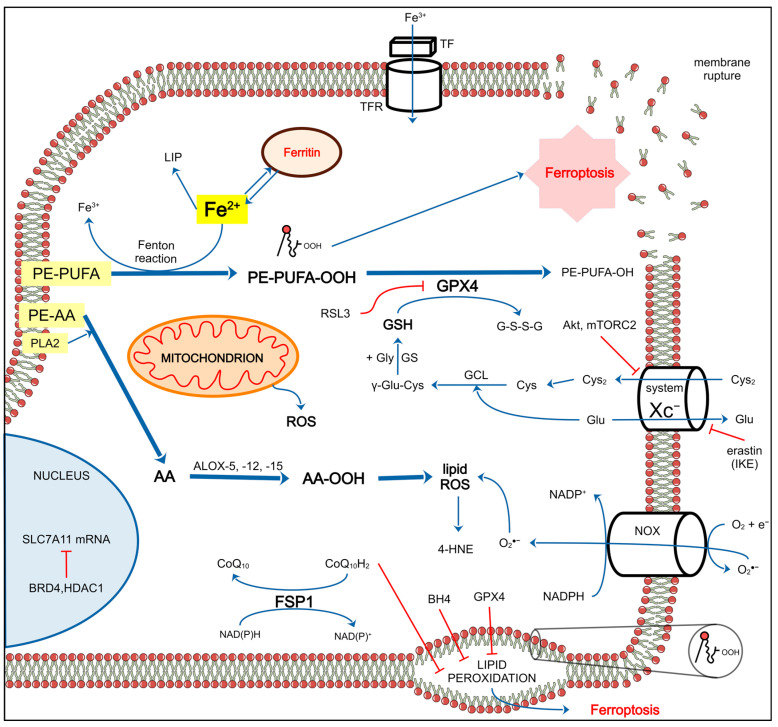
Major processes behind ferroptosis. TF, transferrin; TFR, transferrin receptor; LIP, labile iron pool; PE; ethanolamine plasmalogen (ether-linked phosphatidyl ethanolamine); PUFA, polyunsaturated fatty acid; AA, arachidonic acid; PLA2, phospholipase A2; PE-PUFA-OOH, fatty acid hydroperoxide; PE-PUFA-OH, fatty acid alcohol; GSH, glutathione; GPX4, glutathione peroxidase 4 (phospholipid peroxidase); GS, glutathione synthase; Glu, glutamate; Cys, cysteine; Cys_2_, cystine; GCL, glutamate cysteine ligase; Gly, glycine; RSL3, RAS-selective lethal 3; Xc^−^ system, cystine glutamate antiporter (encoded by *SLC7A11*); SLC7A11, solute carrier membrane protein A11; Akt, protein kinase B; mTORC2, mechanistic target of rapamycin complex 2; IKE, imidazole ketone erastin; ALOX, arachidonate lipoxygenase; NOX, NADPH oxidase; 4-HNE, 4-hidroxynonenal; ROS, reactive oxygen species; BRD4; bromodomain-containing protein 4; FSP1, ferroptosis suppressor protein 1; CoQ, coenzyme Q; BH4, tetrahydrobiopterin; HDAC, histone deacetylase. Red arrows-inhibition.

Ferroptosis is a sort of non-apoptotic oxidative RCD and PCD [314] that starts because of iron-dependent lethal lipid peroxidation [315]. Ferroptosis initiation and execution lie at the intersection of amino acid, lipid, and iron metabolism. Except for p53, which affects both ferroptosis and apoptosis, ferroptosis seems to be independent of other known cell death pathways. Let us mention a few pivotal players in the ferroptotic process: GPX4 (a phospholipid peroxidase), oxygenated polyunsaturated fatty acids (PUFAs) including fatty acid hydroperoxides, ACSL-4, and the peroxisome–ether–phospholipid axis (Figure 4). Hallmarks of ferroptosis are iron excess, ROS release, lipid peroxidation, and cell damage [316]. The danger of Fe^2+^ ion is its ability to catalyze Fenton reactions [317]. While during oxidative stress ROS can injure any biomolecule, DNA, membrane phospholipid, or protein, PUFAs in plasma membrane phospholipids are the preferential targets of lipid (hydro)peroxidation [318]. Oxygenated lipids that trigger ferroptosis may originate from diverse enzymatic or non-enzymatic reactions, but all of them require the presence of iron [319,320].

NADPH oxidase (NOX)-mediated (enzymatic) ROS production may also initiate lipid peroxidation in a cell-type context (Figure 4). Out of all PUFAs, arachidonic acid (FA 20:4ω6) and PUFAs stemming from the same metabolic pathways are particularly vulnerable to oxidation. The arachidonate lipoxygenase enzyme family (ALOX) catalyzes FA 20:4ω6 oxygenation to hydroxyeicosatetraenoic acids (HETEs), such as 5(S)-, 8(S)-, 12(S)-, and 15(S)-HETE, which are products of the respective enzymes ALOX5, ALOX12, ALOX15, and ALOX15B. These are considered key enzymes in controlling ferroptosis in humans [319]. Alternatively, two types of cytochrome P450 oxidoreductases (CYP450) mediate lipid peroxidation in an ALOX-independent manner, creating epoxyeicosatrienoic acids and 20-hydroxyeicosatetraenoic acid (20-HETE) [321]. We have recently conducted a targeted lipidomic study with 17 dnDLBCL female patients, in which we compared plasma concentrations of FA 20:4ω6 and its metabolites-eicosanoids (HETEs, DiHETs) in patients and healthy females. We observed significant elevation in plasma levels of 12(S)-HETE in patients and an upward trend for 15(S)-HETE and 20-HETE. We also created an eicosanoid-based multivariate that could separate DLBCL from healthy samples [274]. Whilst we could not confirm the ferroptosis fingerprint in the plasma lipidomic profiles observed in DLBCL, we are confident there is more to explore in the context of ferroptosis-associated biochemical changes in DLBCL. It seems that lipid metabolism is intimately linked to ferroptosis, so future research should integrate an untargeted deep lipidomic study covering oxidated lipids and oxylipins and a comprehensive transcriptomic study covering lipid- and ferroptosis-associated genes in the same DLBCL cohort, both in treatment-naïve patients and after each line of immuno-chemotherapy. We anticipate that such a study may help explain some of the mechanisms behind R/R disease. We shall elaborate on the role of lipids in DLBCL later.

Unregulated ferroptosis is involved in the development, recurrence, and occurrence of multiple tumors, including DLBCL [322]. Data on 884 DLBCL patients were retrieved from the GEO database, and the relationships between 259 ferroptosis-related genes’ expression and OS were assessed. Ferroptosis-related genes were mined from FerrDb, the world’s first database dedicated to ferroptosis regulators and ferroptosis–disease associations, http://www.zhounan.org/ferrdb (accessed on 20 October 2024). An eight-gene signature prognostic index was established, consisting of *ZEB1*, *PSAT1*, *NGB*, *NFE2L2*, *LAMP2*, *HIF1A*, *FH*, and *CXCL2* [323]. In later research, the prognostic signature for DLBCL was created with only five ferroptosis-related genes: *GOP1*, *GPX2*, *SLC7A5*, *ATF4*, and *CXCL2* [324].

Ferroptosis is currently seen as a novel druggable option in tumor therapy [316,325]. Ferroptosis suppressor protein 1 (FSP1), an oxidoreductase that converts ubiquinone to ubiquinol, has emerged as a critical player in the regulation of ferroptosis, which operates independently of the GPX4 pathway (Figure 4). Being independent of the classical GPX4 pathway makes FSP1 a desirable target for inducing alternative ferroptotic action in ferroptosis-resistant neoplastic cells [325]. We shall outline a few novel findings that deal with ferroptotic mechanisms, prognostic markers observed in DLBCL, and candidates for therapeutic targets. 4-Hydroxy-2-nonenal (4-HNE), a second messenger of free radicals, and the end-product of lipid peroxidation in cells, has been widely acknowledged as a lipid peroxidation marker. 4-HNE may induce gene mutations and alter intracellular signaling [326]. The elevated expression of FSP1 coincides with the nuclear 4-HNE accumulation in DLBCL (both assessed by IHC), and both nuclear and cytoplasmic 4-HNE accumulation and FSP1 positivity are independent predictors of the worse prognosis [327]. The broad anti-tumor effect that dimethyl fumarate (DMF) exerted on both DLBCL subtypes was found to be mediated by the induction of ferroptosis. Especially in GCB-DLBCL, ferroptosis commenced due to a concerted action of the elevated expression of ALOX5 and low levels of both GSH and GPX4. However, in ABC-DLBCL cells addicted to NF-κB and STAT3 for survival, DMF efficiently inhibited the activity of the IKK complex and Janus kinases. Collectively, the findings point to the clinically approved drug DMF as a promising novel therapeutic option in treating both GCB- and ABC-DLBCLs [328].

In an excellent novel work, the same group revealed that the epigenetic reader from the BET family, BRD4, safeguards DLBCL cells from ferroptosis by repressing ferroptosis-related genes (Figure 4). The initial assumption was as follows: to circumvent the infamous DLBCL resistance to apoptosis (immortality), the induction of ferroptosis might be a way, hence a search across the library of compounds targeting epigenetic readers to identify ferroptosis-sensitizing drugs resulted in bingo, BRD4. BRD4 inhibition sensitized GCB-DLBCL cells to ferroptosis induction. So, a combination of BETi with ferroptosis inducers, such as DMF or RSL3, killed DLBCL cells in a concerted manner, both in vitro and in a patient-derived xenograft mouse model of DLBCL [329]. This study also raised questions that are important to address. What is the molecular mechanism behind GCB-cells being more sensitive to ferroptosis than ABC-DLBCL cells? Why are DLBCL cell lines more sensitive to ferroptosis than other tumor cell lines [330]? Indeed, DLBCL cells are more prone to imidazole ketone erastin (IKE)-induced growth inhibition than multiple myeloma (MM) or AML cells [331], which may be due to the dependence of DLBCL cells on the Xc^−^ system for cysteine uptake, since they cannot synthesize cysteine from methionine [332]. IKE is a ferroptosis inducer whose cytotoxic effects on DLBCL cell lines and xenograft models proceed by inhibiting amino acid antiporter Xc^−^ system, which results in GSH depletion, lipid peroxidation and, eventually, ferroptosis (Figure 4). A distinctive lipidomic landscape was revealed in IKE-treated DLBCL cells vs. xenograft models of DLBCL by employing untargeted lipidomics. In IKE-treated samples, the altered lipids chiefly belonged to different glycerophospholipid (PL) classes, such as LPC (lysophosphatidylcholine), PC (phosphatidylcholine), PE (phosphatidylethanolamine), or TAGs (triacylglycerols), which all contained PUFAs. In addition, the study discovered a panel of genes encoding lipid metabolic enzymes to be activated, e.g., enzymes of de novo lipid biosynthesis, PL remodeling pathways, and eicosanoid pathways. The pathway analysis results are concordant with the enhancement of the PL remodeling pathway, which is important for repairing oxidative damage. The activation of enzymatic lipid peroxidation could be seen in this IKE-induced ferroptosis, accompanied by the increased expression of ALOX12 and ALOX15 [332].

While in the plasma of female DLBCL patients we measured increased concentrations of 12(S)-HETE and 15(S)-HETE [274], which are the products of ALOX12 and ALOX15 enzymes, respectively, Zhang and colleagues reported the increased expression of *ALOX12* and *ALOX15*, which is a remarkable overlap [332]. The PL remodeling due to ferroptosis in DLBCL reported by others [332], therefore, also left a fingerprint on the plasma lipidomic profile of DLBCL patients in our study [274].

Ferroptosis induction may now complement chemotherapy, immunotherapy, and radiotherapy in various tumor types. However, it can lead to side effects, including immune cell death, bone marrow impairment, liver and kidney damage, cachexia (severe weight loss and muscle wasting), and secondary tumorigenesis [333].

### 10.4. Mitophagy

Mitophagy, the selective autophagic process that specifically degrades mitochondria, is a vital regulatory mechanism for eliminating damaged mitochondria and maintaining cellular balance. Emerging research underscores the central role of mitophagy in the initiation, advancement, and treatment of malignancies [334]. Mitophagy governs mitochondrial homeostasis in HSCs, influencing their metabolic dynamics [335]. We expect studies on the significance of mitophagy in the context of DLBCL to appear soon.

## 11. Metabolic Changes Relevant to B-Cell NHL and DLBCL

Neoplastic cells can thrive even in a nutrient-deprived TME owing to metabolic reprogramming in the TME, where the immune response directly corresponds to nutrient availability [336]. The flux of amino acids, sugars, and lipids through the metabolic pathways in a tumor is under the control of driver mutations coupled with environmental nutrient availability [337]. Most tumors do not generate energy via the common and efficient TCA cycle but instead through glycolysis (Warburg effect), which is inefficient in energy yield per glucose (Glc) unit. The 200-fold higher glycolysis rate than that in normal tissues helps neoplastic cells produce ATP out of glycolysis even in the oxygen-abundant milieu. This aerobic glycolysis is a hallmark of tumors [338].

### 11.1. Altered Glucose and Glutamine Utilization in DLBCL

At least one of the Glc transporter (GLUT) family members must be dealt with by the tumor to support the augmented Glc consumption while the tumor grows and invades its surrounding. In addition, the upregulation of hexokinase (HK), the initial enzyme in the glycolytic pathway, has been observed in the majority of tumors. Enhanced expression of both GLUT1 and HK was demonstrated in metabolically robust DLBCL cells [339]. A novel single-cell transcriptomics study of DLBCL dissected malignant B-cell populations into B-cell clusters with different activity of TFs. Highly malignant B-cells were enriched with genes linked to aerobic glycolysis. These DLBCL cells, high in glycolysis, promoted an immunosuppressive TME, rich in IFN-primed TAMs but depleted of CD8+ cytotoxic T-cells, which correlated with poor prognosis and an exhausted immune microenvironment in DLBCL [340]. This work confirmed the power of single-cell transcriptomics over classical omics analysis because the former may, within the single tumor, dissect different cell subsets with differential developmental and metabolic programs. The combinatorial effect of two agents targeting two metabolic spots has been recently reported, where the inhibitor of monocarboxylate transporter 1, AZD3965, was co-applied with the inhibitor of mitochondrial respiratory Complex I, IACS-010759, to DLBCL cell lines. When administered separately to DLBCL xenograft models, there was no effect. This proof-of-concept study underscored a potentially actionable approach to target lymphoma metabolism [341].

Besides Glc, amino acids are essential for the proliferation and survival of malignant cells, with glutamine (Gln) being the most common amino acid associated with tumor progression. In the cytoplasm, Gln is converted by GLS into glutamate, which, in turn, gives rise to α-ketoglutarate or GSH for an antioxidant response. Tumors increase Gln uptake by changing the expression of the Gln transporter from the SLC family of transporters, e.g., SLC1A5. Nevertheless, tumors are not picky regarding nutrients; they can survive on Gln, serine, arginine, FAs, cholesterol, or other lipids to maintain their ever-growing metabolic demands. The metabolism of immune cells, including TILs, chiefly relies on Glc, while neoplastic cells prefer Gln in various tumor models. This distinction in the context of nutrients and the expression of genes related to Glc and Gln metabolism is programmable by mTORC1, a master regulator of the cellular metabolic state [342]. mTORC1 is also critical for adipogenesis and the maintenance of fat tissues [343].

A recent review dismantled the intricate metabolic competition for Gln between neoplastically transformed and immune cells, where the authors called it a “tug-of-war”. Therefore, targeting Gln transporters and the enzymes of Gln metabolism holds significant promise in enhancing anti-tumor immunity [344]. GLS1 expression is repeatedly observed in DLBCL biopsy samples and cell lines, so the pharmacological inhibition and genetic knockdown of GLS1 trigger DLBCL cell death, whichever the subtype. In contrast, primary B-cells remained unaffected. Encouragingly, the tandem application of the GLS1i telaglenastat and therapeutic BCL2i (ABT-199) induced massive ROS production and cytotoxicity. Simultaneous targeting of GLS1 and BCL2 could represent a novel therapeutic strategy for DLBCL. Importantly, human primary B-cells activated by plant lectin were mainly resistant to telaglenastat, indicating that only transformed DLBCL cells depended on glutaminolysis [239].

### 11.2. Lipid Metabolic Reprogramming in DLBCL

The role of lipids in the tumor-signaling nanouniverse is an ever-growing research field. Although neoplastic cells metabolize a considerable amount of carbohydrates, it is insufficient to fulfill their needs, so they also amplify lipid metabolism. For tumors, it is not all about satisfying energetic needs; proliferating cells also require diverse complex lipids, such as PLs and sphingolipids (SLs), plus ample amounts of cholesterol to build their cell membranes and sustain a heightened level of cellular signaling [345,346]. Compared to the literature data on lipid reprogramming in solid cancers, data on hematological malignancies are relatively scarce [347]. Unfortunately, that is not going to change soon.

FAs are versatile biomolecules used for energy production through β-oxidation, as substrates for mitochondrial production of ATP and NADH, as building blocks for the biosynthesis of membrane PLs and SLsm and so on. Furthermore, arachidonic acid is a precursor for eicosanoids, crucial inflammatory/immune mediators. ω3 PUFAs are needed for the biosynthesis of dozens of lipid mediators, e.g., resolvins, which mediate a resolution of inflammation. FAs can be found covalently attached to membrane proteins, which keeps them buried in the lipid sea of plasma membranes [348]. Malignant cells intensify de novo synthesis of FAs, i.e., lipogenesis, to keep growing [349]. An obligate step to intensify lipogenesis is to upregulate two rate-limiting enzymes, acetyl-CoA carboxylase and fatty acid synthase (FASN), with the latter converting acyl-CoA to long-chain FAs. As a rule, FASN is overexpressed in tumors [350], and that is also the case with hematological malignancies and lymphomas [351]. Increased expression of FASN in 62.6% of DLBCL tissue samples was reported, which was also correlated with high Ki67 (*p* < 0.0001), marking highly proliferative tumors [352]. Aberrant FA metabolism is supportive of aggressive B-cell lymphomas, including BL and DLBCL [353]. FASN is constitutively activated in DLBCL, which was observed both on the mRNA level and the protein level. The oncogenic pathway of HIF-1α, which is also constitutively activated in DLBCL, directly regulates FASN expression [354]. In a further excellent study by the same group, elevated levels of FASN were repeatedly measured in all DLBCL cell lines, regardless of COO. Blockage of FASN performed by cerulenin (FASN inhibitor) orlistat (FASN and lipoprotein lipase inhibitor), or BKM120 (PI3Ki) was investigated in several DLBCL cell lines and primary DLBCL cells. The constitutive activation of diverse FA pathways was revealed, and, importantly, DLBCL cell survival was strongly reliant on lipid metabolism, regardless of COO. The mechanism of FASN inhibition was, in part, ascribed to PHB/PI3K-induced pathways [355]. This pioneering work marked cycles of for pursuing a valid therapeutic strategy for DLBCL. Selective inhibition of FASN by the novel drug, Fasnall, was demonstrated in a panel of DLBCL cell lines, implying the therapeutic potential of interference with FA synthesis, especially in a subset of DLBCL cells that preferentially utilize de novo FA biosynthesis [356].

FA oxidation (FAO) is a convenient energy source for the growth, progress, and dissemination of lymphoma cells. Aggressive B-cell lymphomas indeed satisfy their energy needs through palmitate oxidation. However, the situation is more complex, as only CD37-deficient lymphomas can use palmitate because the membrane protein tetraspanin, CD37, inhibits a FA transporter by directly binding to it. Large extracellular lipid deposits and lipid droplets inside cells were seen in CD37-negative lymphoma tissues of patients. The immense importance of this study lies in discovering CD37 as a gatekeeper of the metabolic switch to FAO in aggressive B-cell lymphoma. In addition, CD37 represents a new promising target in aggressive B-cell lymphoma [357]; see about CD37-directed naratuximab emtansine later in the text.

The transportation of FAs from the extracellular space to inside of cells is the first step of FAO, which proceeds via respective FA transporters located on the cell surface. The most well-described FA transporters include CD36, solute carrier protein family 27 (SCL27), and FA binding proteins (FABPs). CD36 can transport long-chain FAs, oxidized-LDLs, anionic PLs, and oxidized PLs across the cellular membrane. Augmented CD36 expression is characteristic of many tumors, including DLBCL. Furthermore, the CD36 expression profile is highly associated with disease stage and metastatic status [358]. CLL patient-derived neoplastic cells exhibited significantly higher CD36 levels than normal CD19+ B-cells; these CLL cells in culture, owing to a STAT3-driven metabolic adaptation, were able to metabolize FFAs in the cell medium [359]. Finally, the overexpression status of both FASN and CD36 predicts the OS in RTX-treated DLBCL patients. Poor prognosis was observed in patients with high IPI and overexpressed FASN, whereas patients with low IPI had worse prognosis if they also harbored high FASN expression. The authors suggested that “lipogenic phenotype” contributed to disease aggressiveness in DLBCL [360].

In order to maintain heightened cell proliferation, malignant cells also need increased cholesterol supply to build cell membranes. However, in some malignant cells, including lymphoma, de novo cholesterol biosynthesis genes are silenced or mutated, meaning the tumor must turn to cholesterol uptake from lipoproteins for survival. The cholesterol is picked up from circulation by cholesterol protein transporters, such as the low-density lipoprotein receptor [361]. Despite the rapidly growing evidence of cholesterol’s involvement in tumor pathogenesis and progression, studies on cholesterol metabolism change in lymphomas, including DLBCL, are relatively rare compared to those on solid cancers. We will outline a few of them. A retrospective study informed us that advanced DLBCL cases who were treated with frontline R-CHOP plus statins (HMG-CoA reductase inhibitors) had much better clinical outcomes than those who were given only R-CHOP [362]. Moreover, targeting lipid metabolism is a strategy to overcome BTKi resistance in DLBCL [363]. The binding of cholesterol-rich HDL to the scavenger receptor type B-1 was detected in lymphoma cells dependent on cholesterol uptake [364], which is of importance because it shows the metabolic plasticity of the lymphoma cells when it comes to satisfying their needs for specific nutrients.

EBV can trigger FASN overexpression and augment the rate of lipogenesis in B-cells via ectopic expression of the viral latent membrane protein 1 (LMP1), the chief transforming protein of EBV-mediated B-cell growth transformation. The authors proposed that lipogenesis inhibitors could be included in the treatment of LMP1-positive B-cell malignancies [365].

Our earlier study analyzed plasma FA status in NHL patients and its relationship with disease aggressiveness and clinical stage. In NHL, we have detected significantly higher (*p* < 0.001) levels of palmitic, oleic, and arachidonic acid, total saturated FAs, and monounsaturated FAs. On the other hand, the content of linoleic acid (18:2ω6), total PUFAs, total ω3 PUFAs, eicosapentaenoic acid, and docosahexaenoic acid was significantly lower than in healthy individuals (*p* < 0.01). Very aggressive NHLs left their fingerprint on plasma FA status, so the levels of palmitoleic (16:1) and docosatetraenoic (22:4 ω6) acids were lower than in other NHLs [366]. We have also shown that the total plasma PLs and individual PL classes (PL profile) significantly differed in therapy-naive NHL patients compared to healthy persons. We retrospectively divided the 40 patients into three groups based on the RTT: complete remission, stable disease (SD), and progression (PG). We could not detect differential PL profiles among complete remission, SD, and PG at the baseline. However, after chemotherapy, the total levels of plasma PLs and levels of individual PL classes significantly differed and were associated with the clinical outcome in patients with NHL. Strikingly, all measured PLs dropped in the plasma of PG patients, in contrast to the responders (complete remission and SD), where these PLs were significantly increased [367]. We must emphasize here that the changes in individual lipid species’ levels in the plasma of tumor patients cannot yet be explained because these alterations reflect numerous aberrations in lipid metabolism, even the conflicting simultaneous activation of lipid biosynthetic and breakdown pathways. The other group reported significantly higher serum-free FA (FFA) levels in 361 dnDLBCL patients compared to the control group (*p* < 0.0001), and also that the increased levels of FFA were associated with lower survival in untreated DLBCL patients [368]. The study’s limitation is that only the total level of FFAs was measured. Hence, although the study confirmed the general mobilization of FAs by the tumor, only the levels of individual FFAs warrant a deeper understanding of FA reprogramming in DLBCL.

Unique lipid profiles of in vivo xenograft models of R-CHOP-resistant tumors were revealed, including a higher presence of PI and sphingomyelin (SM) fragments. Of note, fragments of complex lipids are due to the laser breakdown of these lipid molecules during MALDI MS [369]. We have also reported increased levels of the three most abundant SM species in the plasma of female DLBCL patients [274].

Dyslipidemia was detected in 87.0% (*p* < 0.001) of 259 dnDLBCL patients. Specifically, the EZB subtype manifested an increased incidence of dyslipidemia, particularly hypertriglyceridemia, compared to other DLBCL subtypes. Moreover, patients with *BCL2* fusion mutation exhibited pronounced hyperlipidemia (76.5%) and hypertriglyceridemia (88.2%). However, despite being correlated with genetic heterogeneity in DLBCL, the lipidemic status did not significantly affect the survival of patients [370]. The study was the first to link lipid status to the genetic subtypes in DLBCL. The genetic architecture and heritability of plasma lipidome and genetic associations between diseases and plasma lipid species, beyond the classical clinical lipids, LDL-C, HDL-C, total cholesterol, and TAGs, have emerged from big population studies [371,372,373]. The latter explored the link between the genetics of human plasma lipidome and cardiovascular diseases [373]. We expect many similar studies on the genetic associations of plasma lipidome and different malignancies to follow.

Low levels of circulating cholesterol are related to higher tumor incidence and mortality, although we think it may be a consequence rather than the cause of tumor burden. Tumors need a lot of cholesterol to build cell membranes, hence cholesterol is taken up from the plasma depots. The greater the stage, the lower the cholesterol. Low serum cholesterol levels mark poor prognosis in DLBCL. DLBCL patients achieving complete remission or partial remission after 6–8 cycles of chemotherapy had significantly increased cholesterol levels compared to the levels at diagnosis, and HDL-C or LDL-C elevations were correlated with better survival [374]. Since lipid species are not free in circulation but rather bound to plasma lipoproteins, e.g., LDL, HDL, and since the equilibrium of the lipoproteins could be altered in malignancy, we anticipate that the lipid composition of these lipoprotein particles could also be altered. The most novel study reported low serum ApoA1 levels associated with poor RTT and inferior OS in 1583 dnDLBCL patients [375].

The attempts of the community to discover and hit a suitable target within or related to the lipid metabolism for tumor therapy have encountered many obstacles due to the lack of a deep understanding of lipid metabolism and all the layers that regulate it. Whilst the advent of comprehensive lipidomics brought novel insights into lipid reprogramming induced by tumors, these studies mainly dealt with solid cancers. Male and female plasma lipidomes significantly differ [376,377,378], which must be taken into an account when planning plasma lipidomic studies in malignancy. However, it would be interesting to see whether the lipidome of tumor biopsies is also dependent on gender. Regarding hematological malignancies and DLBCL, we are still waiting for the deep sequencing of lipidomes from the tumor biopsy samples.

## 12. Gut Microbiota in DLBCL

With approximately 100 trillion microorganisms in the human gut, representing one to three percent of the body’s weight, the GMB is considered the body’s other major organ and affects health via an ancient evolutionary symbiotic relationship [379]. The genome of the microbial community in the human gastrointestinal system is >150 times that of the human genome. Hence, the microbial genome in the human body can be considered the second human genome. This collection of microbes in the gut is called the GMB; the entirety of their genes is called the gut microbiome (GMBm) [380]. The GMB, consisting of diverse microorganism communities including viruses, bacteria, fungi, and eukaryotes colonizing human body surfaces, has recently been identified as a contributor to inter-individual variation through its person-specific signatures. As such, the GMBm may modulate disease manifestations, even among individuals with similar genetic disease susceptibility risks. Information stored within microbiomes may, therefore, enable the early detection and prognostic assessment of diseases in at-risk populations, whereas microbiome modulation may constitute an effective and safe treatment tailored to the individual [381]. Now, let us present data on GMBm involvement in lymphoma, particularly in DLBCL.

The GMB has emerged as a critical player in lymphoma, affecting host physiology, but the molecular mechanisms underpinning lymphoma that involve gut players are hidden from us. The most obvious, but too simplistic, guess is that dysbiosis of the gut microbial community disrupts the production of gut microbial metabolites, thereby impacting host physiology and potentially contributing to lymphoma [382]. The first revelation of microbial species residing in the gut of untreated DLBCL patients was the work of Yuan and colleagues, where they analyzed fecal bacteria from 25 dnDLBCL patients vs. 26 healthy volunteers using 16S rRNA gene sequencing, which is the standard analysis of GMBm. When comparing two microbiomes, the differences are commonly expressed as alpha or beta diversity. The former reflects species diversity and abundance differences, and the latter refers to the differences in overall taxonomic composition between two samples. The study enrolled comparable numbers of GCB and ABC cases in the DLBCL group. While alpha-diversity analysis showed no difference in species abundance or diversity, beta-diversity analysis exhibited significant alterations in the microbiota composition in DLBCL. DLBCL hallmarks were as follows: a higher abundance of phylum *Proteobacteria* and the genus *Escherichia*-*Shigella*; *Proteobacteria* was the dominant microbiota in the untreated DLBCL patients, which lays a solid foundation for further exploration of whether *Proteobacteria* may be targeted as a novel therapeutic target for treating DLBCL; and at the genus level, *Allisonella*, *Lachnospira*, and *Roseburia* were more abundant in patients with DLBCL than in the controls. A low abundance of *Bacteroides fragilis* in DLBCL patients was also observed [383]. We want to emphasize the strong point of the mentioned study, which is the recruitment of an equal number of males and females. Several animal and human studies showed gender differences in GMB [384], though the results are inconsistent. Of note, *Bacteroides* spp. are generally ‘friendly’ commensals as the majority of the healthy GMB is composed of *Bacteroides* spp., which metabolize polysaccharides and oligosaccharides (dietary fibers), providing nutrition and vitamins to the host and other intestinal microbial residents [385].

Mamgain et al. reviewed the role of GMB in the development of T-cell and B-cell lymphoma, accentuating GMB as an important contributor to developing lymphoma, such that specific alterations to microbiota composition could attenuate the risk of lymphoma [386]. The suspected mechanisms behind how viruses and microorganisms affect our health and contribute to carcinogenesis (and lymphomagenesis) are as follows: (i) the insertion of bacterial DNA into the human somatic genome (lateral gene transfer), known to be enriched in tumor samples [387]; (ii) microbial metabolites or genotoxic small molecules produced by specific strains in the gut may change human DNA, like how colibactin from *E. coli* alkylates DNA [388]; and (iii) a microbial community change may trigger the immune response in the gut. On the flip side, good bacteria help their host with healthy living by promoting epigenetic mutations, e.g., butyrate produced by some gut bacteria inhibits HDAC-6 and -9 and further enhances acetylation at the *FOXP3* gene promoter [386].

A pilot study pointed out that microbial dysbiosis is associated with the aggressive histology and adverse clinical outcome of B-cell NHL, including DLBCL. Not only did the pre-treatment GMB in lymphoma patients have a distinct composition compared to the healthy controls, but the GMB compositions in DLBCL patients were also significantly different from those in indolent patients, with a trend toward reduced microbial diversity in DLBCL patients. Expectedly, pre-treatment GMB diversity and composition were significant predictors of treatment responses [389]. The correlation between the GMB diversity in 35 dnDLBCL patients and their immune status, including peripheral blood immune cell count and cytokine levels, was revealed with *p_Proteobacteria* dominating the gut of patients, while *p_Bacteroidetes* abundance was decreased when compared with healthy controls. The authors proposed that particular bacteria may affect anti-tumor immunity via IgA and that higher *g_Butyricimonas* abundance was putatively associated with DLBCL remission after chemotherapy [390]. A deep sequencing of the gut microbiome in DLBCL patients before, during, and after therapy is needed to decipher the potential therapeutic approach, e.g., adjuvant therapy. Bacterial–human lateral gene transfer has been detected in CLL [391] but not in DLBCL.

Knowing that many dietary regimes, including Western, vegan, gluten-free, omnivorous, and Mediterranean, are capable of controlling GMB [392,393] and that diets might be responsible for 30% of tumor cases in developed countries and 20% in developing countries [386], it is expected to be more explored in lymphomas and tumors in general. A prominent study of microbiota in stool samples discovered that DLBCL patients had profound intestinal dysbiosis at the time of diagnosis. As a rule, the alpha diversity was significantly lower in DLBCL than in healthy patients, and the microbial composition differed significantly between the groups. *Enterobacteriaceae* members were significantly enriched in patients who experienced febrile neutropenia and in those who experienced relapse or progression after R-CHOP chemotherapy [394]. The study was the largest analysis of the prognostic impact of stool taxonomic composition in dnDLBCL cases ever described [395], but we are sure there are more to come. The causal relationship between GMB composition and the efficacy of chemotherapy in DLBCL patients was assessed, and differences in GMB were observed between healthy controls, patients prior to treatment, and treated patients. The chemotherapy efficacy seemed to be correlated with GMB, but GMB diversity across genetic subtypes, ABC- and GCB-DLBCL, was not observed [396]. The origin and role of gut dysbiosis necessitate further studies.

The microbiota landscape of 87 dnDLBCL patients, dissected by metagenomic sequencing, revealed 10 bacterial phyla, 31 orders, and 455 bacterial species. *Streptococcus parasanguinis* and *Streptococcus salivarius* were markedly accumulated in the NCCN-IPI’s high-risk group. Specifically, the abundance of *Bacteroides salyersiae* was negatively correlated with regulatory T-cells (Treg), CD38+ non-rescue exhausted T-cells, NK3 cells, and CD38+CD8+ effector memory T-cells. The abundance of *Streptococcus parasanguinis* was negatively correlated with HLA-DR+ NK cells, CD4+ Treg, HLA-DR+ NKT cells, and HLA-DR+CD94+CD159c+ NKT cells [397], thus revealing GMB and TME inter-dependence. The most recent work on causal relationships between GMB, DLBCL, and inflammatory cytokines revealed four microbiota taxa causally related to DLBCL. Still, DLBCL influenced the abundance of 20 taxa, i.e., there is complex interplay where GMB helps the development of the lymphoma, and vice versa, lymphoma itself affects the GMB composition. Genus *Ruminococcaceae* UCG-002 and inflammatory cytokine monokine induced by IFN gamma were causally linked to an increased risk of DLBCL [398].

Exposure to antibiotics strongly affects the GMB composition. Antibiotics are routinely administered to DLBCL patients before CART therapy, particularly piperacillin/tazobactam, meropenem, and imipenem/cilastatin. This combination administered within the 4 weeks before CART therapy corresponded with decreased alpha diversity, leading to worse survival and increased neurotoxicity. Clinical outcomes were associated with differences in specific bacterial taxa and metabolic pathways [399]. A similar multicenter cohort of R/R lymphoma, including DLBCL patients, showed that wide-spectrum antibiotics exposure (‘high-risk antibiotics’) prior to CD19-targeted CART therapy is associated with adverse outcomes, but this effect is likely to be confounded by an increased pre-treatment tumor burden and systemic inflammation in patients [400].

The immune function in DLBCL patients can be improved by regulating the GMB. To escape the vicious circle of intestinal *Enterobacteriaceae*, systemic inflammation, immunosuppression, febrile neutropenia, and relapse, one would think about first restoring “gut eubiosis” and intestinal barrier fitness before R-CHOP treatment [395]. This issue could be addressed through interventions such as fecal microbiota transfer from a healthy donor to the intestine of a recipient (already tested in the treatment of resistant *Clostridium difficile* infection or inflammatory bowel disease); and/or introducing a fiber-rich diet, prebiotics, and probiotics. A diet enriched with dietary fibers plus specific prebiotics/probiotics could help increase the content of short-chain FA producers in the gut. These approaches are challenging because many studies were performed on murine models. Hence, their findings cannot be taken for granted and directly translated to humans, knowing that mice and humans have different GMBs and adaptive immune systems [401].

We are still waiting for confirmatory results from clinical trials. There is promise for future research in this emerging field of relationships between lymphoma and GMB. Out of hundreds of microbial metabolites that carry the potential to help in therapy due to their immunomodulatory and/or anti-tumor properties, we can mention a few: short-chain fatty acids, inosine, urolithin A, and urolithin B [382].

## 13. Molecular Modus Operandi of Drugs Used to Treat B-Cell NHL and DLBCL

Non-targeted anti-tumor therapy is basically the non-selective killing of malignant cells, which is accompanied by the death of many healthy cells and a high level of toxicity. It has been almost five decades since the first use of a CHOP chemotherapeutic mixture for treatment of lymphomas including DLBCL [402]. All four agents in the CHOP mixture are able to trigger apoptosis in tumor cells. Cyclophosphamide alkylates DNA, thus preventing its duplication or transcription to RNA. Cyclophosphamide is metabolized by liver enzymes, e.g., CYP450, into phosphoramide mustard, an active alkylating agent responsible for the great majority of the anti-neoplastic effects of cyclophosphamide. Since modifications to DNA induced by alkylation are impossible to reverse, they eventually lead to death by apoptosis. The major drawbacks of cyclophosphamide are its immunosuppressive effects and selectivity for T-cells. Doxorubicin (anthracyclin) intercalates between bases in DNA, thus inhibiting the progression of topoisomerase II, an enzyme that relaxes supercoils in DNA for transcription. It may also increase quinone-type free-radical production, thus contributing to cellular cytotoxicity. Vincristine, marketed as Oncovin (hence O in CHOP), is a vinca alkaloid, able to bind to tubulin, thus stopping polymerization into microtubules. Without microtubules in mitosis, chromosomes cannot be separated, so the cell undergoes apoptosis. Glucocorticoid prednisone is used for immunosuppression and as an anti-inflammatory drug due to interference with the migration of polymorphonuclear leukocytes and reversed increased capillary permeability.

### 13.1. Targeted Therapy of B-Cell NHL and DLBCL

Targeted therapy has been a long-dreamt dream in the treatment of malignancies, which is also true for hematological malignancies. Chief among the targeted therapies is small-molecule inhibitors targeting essential oncogenic signaling proteins and enzymes. Tumors can be slowed or even completely eradicated by competitive and non-competitive inhibition of these proteins/enzymes and, hence, the pathways they activate, leading to partial or complete remissions for many tumor types. Unfortunately, for many patients, resistance to targeted therapies ultimately develops and can necessitate multiple lines of treatment. Drug resistance can either be de novo or acquired after months or years of drug exposure. Since this resistance can be due to several unique mechanisms, a one-size-fits-all solution cannot heal all tumors [403]. We have already discussed small-molecule BTKi earlier in the text, so we shall proceed to immunotherapies designed to attack the surface antigens on B-cells. Let us first briefly describe the most therapeutically interesting B-cell markers.

### 13.2. B-Cell Markers as Targets of Anti-Neoplastic Strategy

B-cell markers are surface-exposed protein antigens of B-cells, such as CD19, CD20, CD22, CD37, CD40, CD79A, and CD79B, to list a few. They can be differentiation-specific or pan B-cell markers. Pan B-cell markers are expressed on all B-cells, though not at the same extent, and they include CD19, CD20, CD22, and CD79B. In brief, the key markers of most mature B-cells are IgM and CD19; activated B-cells will express CD30, a regulator of apoptosis. Plasma B-cells lose CD19 expression but gain CD78, which is used to quantify plasma cells. Memory B-cells can be immunophenotyped using CD20 and CD40 expression. We shall not list all the B-cell markers or their nature and roles in B-cells’ lives because plenty of them have been described elsewhere [404,405]. Instead, we will mention only those that are currently used as targets for approved and under-research immunotherapeutic drugs for B-cell lymphomas.

CD19 is expressed on all B-cell subsets, starting from their earliest differentiation stages in the bone marrow and remaining there until the plasma cell stage. CD19 is uniformly expressed across all B-cell lymphomas, including DLBCL; it even survives on small subsets of CD20-negative B-cell lymphomas evolving after anti-CD20 immunotherapy [406,407]. CD19 has been a favorite target for immunotherapy of B-cell lymphomas owing to its efficient internalization, limited off-target actions, and high disease specificity [408]. Tafasitamab is a humanized IgG1 mAb with an Fc region that was engineered with the purpose of achieving a higher affinity for the Fcγ receptor (FcγR) and augmented cytotoxicity, executed by NK cells and macrophages through ADCC and antibody-dependent cell-mediated phagocytosis (ADCP) [409,410,411].

The membrane-spanning 4-domain family A (MS4A) member CD20 is a non-glycosylated transmembrane protein protruding from the surface of normal and malignant B-cells. CD20 is ubiquitously expressed in B-cells, starting from late pre-B lymphocytes, but is lost in plasmablasts and plasma cells [412]. CD20 expression varies to a great extent in the context of different B-cell malignancies, but also among patients with the same malignancy, and even among intra-clonal subpopulations in an individual patient. Although CD20 is a classical B-cell marker, a subset of CD20+ T-cells is repeatedly observed, and these cells express more inflammatory cytokines [413]. Though the clinical relevance of this is not clear, we should be aware of the potential off-target effects of anti-CD20 mAb acting on such T-cells. CD20 is decreased in response to ibrutinib, hence preventing the successful administration of ibrutinib + anti-CD20 RTX. Anti-CD20 mAbs might act through complement-dependent cytotoxicity (CDC), complement-dependent cellular cytotoxicity (CDCC), ADCC, ADCP, or directly triggering apoptosis. CD20 antigen loss or down-modulation in response to anti-CD20 immunotherapy can hypothetically occur through “shaving” (trogocytosis) or selective elimination of the B-cells enriched with surface CD20. These two mechanisms were demonstrated in CLL/SLL, which is characterized by monoclonal B-cell proliferation, but not yet in FL or DLBCL. Another mechanism is the active internalization and degradation of CD20/mAb adducts [414], which occurs only with type I anti-CD20 mAbs, such as RTX and ofatumumab, but not type II mAb such as obinutuzumab [415]. The selection pressure on B-cells treated by RTX is associated with the emergence of malignant B-cell clones that are relatively or entirely CD20-negative [414]. Mutations affecting the CD20 epitope where RTX binds are sporadic [416].

CD22 is a cell-surface type I transmembrane glycoprotein and sialic acid-binding receptor mostly restricted to B-cells [417,418]. CD22 operates via ADCC and, most of the time, inhibits B-cell functions. The uniqueness of CD22 lies in its ability to associate with the BCR physically, hence many think its function is to inhibit BCR signaling. CD22 is upregulated in B-cell malignancies, including most lymphomas. Therefore, we have yet another potential drug target [418,419]. However, monotherapy of R/R DLBCL patients with humanized anti-CD22 mAb (epratuzumab) did not achieve the expected success [420], most likely due to the cohort’s features, where patients were heavily pre-treated (four prior therapies) or had bulky disease. While mAb directed toward CD22 had stopped being developed, that is by no means the end for CD22-targeted immunotherapy, as CD22 will still be incorporated into respective antibody–drug conjugates (ADCs), CARTs, bispecific antibodies (BsAbs), and bispecific T-cell engagers (BiTEs) [419]. Since CD22 is also an endocytic receptor, it is an ideal target for ADCs, which can effectively transport the cytotoxic payload into the cell [418,419].

Other promising B-cell markers in the therapeutic context are CD79B, expressed in more than 90% of B-cell lymphomas, CD30, CD37, and CD70. CD30 is a transmembrane tumor necrosis factor receptor (TNFR), whose expression is restricted to a small population of activated B-cells, T-cells, and NK cells. Lower levels are present in activated monocytes and eosinophils [421]. In one study, CD30 was identified in approximately 20% of 1048 dnDLBCL cases. The CD30+ DLBCL subtype is a diverse entity, with subsets differing in the scope of mutations, transcriptome, TME landscapes, and clinical outcomes, presenting a rationale for future mechanism-based targeted therapy of DLBCL [422]. CD37, a transmembrane protein whose physiological function(s) we have yet to explore [357], is also a desirable target. There is mounting evidence of its involvement in anti-tumor immunity and immune evasion [423]. Healthy tissues present with a restricted spread of CD37, which is absent in early progenitor B-cells and plasma cells [424]. In contrast, CD37 is richly expressed on most subtypes of NHL and DLBCL. Since unconjugated anti-CD30 mAb (SGN-30) exhibited limited clinical activity, researchers aimed to create an ADC, leading to the discovery of brentuximab vedotin [425].

CD70 is a member of the TNFR superfamily and a unique ligand of CD27. CD70 is transiently expressed in antigen-activated B-cells, T-cells, NK cells, and mature dendritic cells, but also in hematological malignancies. The CD27–CD70 interaction is an immune checkpoint [426]. In a healthy physiological setting, tightly controlled expression of both CD70 and CD27 plays a role in the co-stimulation of the immune response, but malignant cells co-expressing CD70 and CD27 promote stemness, proliferation, and survival of hematological malignancy [426,427]. The community has recently “discovered” CD5, typically expressed by T-cells but also by B1-cells (innate B-cell population), albeit at lower levels [428].

B-cells are back in fashion and their phenotypic and functional diversity, biology, mechanisms controlling different fates of B-cell populations, and role in immune memory are hot research topics; hence, deep and comprehensive studies and reviews covering the topics mentioned above have been published in highly cited journals [183,429,430,431,432].

## 14. Naked Monoclonal Antibodies in DLBCL Therapy

Therapeutic anti-neoplastic mAbs can be naked or conjugated to other drug(s) or other mAbs (or their fragments). Naked mAbs are either humanized or fully human. The former are chimeras generated either by a fusion of part-mouse and part-human proteins (-ximab) or by a small part from mouse and a significant part from human Ig (-zumab). Fully human mAb (-umab) is the best option because of intrinsic deficiencies in the mouse immune system [433]. RTX, anti-CD20 mAb, is among the first chimeras with mouse Fv, human IgG1 heavy chain, and human κ light chain constant regions [434,435]. RTX targets loops H1, H2, H3, and L3 of the extracellular domain of CD20 [436,437]. It is worth noting how chimeric Abs with mouse Fv, as is RTX, can still be seen as non-self, leading to a human anti-chimeric Ab-induced immune response, which may cancel out therapeutic mAb. To further reduce an unwanted immune response, the human content of mouse mAbs must be increased as much as possible [434]. Whilst RTX was developed many years ago and has been in clinical use for three decades, it is incredible that we are still learning about its paratopes and actions, e.g., [436]. This fact yet again confirms that we are still far from having deep knowledge or molecular details on what, how, and why anti-neoplastic drugs are doing what they are doing in the cell, tissue, and organism.

Obinutuzumab (GA101, trade name Gazvya) is a glycoengineered, Type II anti-CD20 mAb, modified to augment anti-CD20 activity and circumvent tumor resistance to RTX. The operative idea behind glycoengineering was to overexpress MGAT III and Golgi mannosidase II so that they introduce N-glycans that mostly lack core-fucosylation onto IgG. The unique N-glycan chains bring unique properties to obinutuzumab, distinct from regular IgG1 [438]. Obinutuzumab exhibited superior behavior over RTX, albeit only in pre-clinical settings. The benefit conferred by obinutuzumab over RTX may be context-specific and vary based on the histological subtype and immune integrity of tumor [439].

## 15. Antibody–Drug Conjugates in DLBCL Therapy

ADCs are targeted immunotherapeutics extensively covered in the literature with dozens of reviews on every aspect of their design, mechanics, and clinical landscape (see, e.g., [440,441,442,443]). ADCs are critical therapeutic modalities in oncology, with 90% of targets being antigens highly expressed on neoplastic cells [444]. A typical ADC is a conjugate of a mAb and a cytotoxic small molecule (cytotoxin) kept together through a short linker. The role of the mAb is targeted delivery of cytotoxin (payload). ADCs operate via the Trojan horse strategy: mAb locates a target antigen on the surface of a tumor cell, mAb binds antigen, the complex is internalized into the tumor cell by endocytosis, and cytotoxin is released inside the cell and kills it. The tutorial on ADCs elaborates on the main advantages of ADCs over both naked mAbs and small-molecule cytotoxins, as well as ADCs’ major hurdles. The chief advantage of ADCs over small-molecule-based therapeutics is that ADCs specifically target only their surface antigens, such as CD19, CD22, CD79b, etc., in B-cell lymphomas. However, ADCs will also kill healthy B-cells that carry these antigens. Currently approved targets of ADCs in hematological malignancies are CD19, CD22, CD30, CD33, and CD79b. The phenomenon in which the payload from ADCs diffuses into adjacent tumor cells even if the cells do not express the target antigen, resulting in cell death, is termed “bystander killing”, which is due to premature linker cleavage. Bystander killing can be good or bad, with the latter due to cytotoxin escape into the systemic circulation [440]. Still, to kill the cell, the payload must first cross the plasma membrane of the tumor cell, which is not feasible if the payload is not a lipophilic molecule [440,445]. mAbs for ADC design ideally target antigens with high specificity and high binding affinity, while ADCs exhibit minimal immunogenicity and a reasonably long half-life. Then, it appears that the IgG1 subclass of mAbs is the most popular for incorporation into ADCs for their stability in plasma and potency to activate the classical complement pathway, in addition to the higher binding affinity toward FcγRs on effector cells [440]. The linkers to be used in ADCs are a story for themselves because linkers also affect various ADC features such as toxicity, specificity, stability, and potency. Linkers are either cleavable or non-cleavable in vivo [446,447]. Many issues affect the efficacy of ADCs, such as the average number of cytotoxin molecules per Ab, pharmacokinetics of ADCs plasma clearance, hydrophilicity and chemical nature of linker and payload, and so on. ADC payloads are too toxic to be used alone; they are typically 100–1000 times more potent than cytotoxic small molecules used as anti-tumor drugs. The payload classes are not as diverse, at least not for now; generally, either tubulin inhibitors or DNA-interactive compounds are combined with Abs. The payloads are from any of the three families of cytotoxins: calicheamicins, auristatins (e.g., monomethyl auristatin E, MMAE), and maytansinoids [440]. Calicheamicins are anti-tumor antibiotics with potent activity against Gram-positive and Gram-negative bacteria and human tumor cell lines; they bind to the minor grove of the double-helical DNA, causing double-strand DNA breaks and cell death. Duocarmycins are minor groove binders and natural products that cause apoptosis but are hepatotoxic. Maytansinoids, such as mertansine (DM1) or DM4, are derived from natural plant products. They induce mitotic arrest through the binding of tubulin at the vinca-binding site, so they inhibit tubulin polymerization (microtubule assembly inhibitors). Pyrrolobenzodiazepines (PBDs) are DNA-interactive anti-neoplastic agents (the first-in-class is anthramycine), minor groove binders, and DNA alkylators that cause cell death [448].

The therapeutic landscape of ADCs in hematological malignancies and lymphoma has also been covered in depth. Two ADCs have been recently approved for therapy of R/R DLBCL: Polatuzumab vedotin-piiq, or Pola, in June 2019, but only in combination with bendamustine and RTX [449], and Loncastuximab tesirine-lpyl (Lonca) [450]. Pola is a second-generation anti-CD79b ADC composed of a humanized IgG1 mAb [451], conjugated to MMAE, a microtubule assembly inhibitor. Conjugation is reached through a maleimidocaproyl-Val-Cit-PABC protease-cleavable linker, enabling the deliverance of the drug directly into malignant B-cells [452]. Polatuzumab mAb is raised against the amino acid sequence ARSEDRYRNPKGS in CD79b [451]. It also binds flexible sequences at the N-terminus of CD79b, as was recently discovered by cryo-EM [453]. Not only does Pola exhibit inhibitory action on Akt and ERK pathways but it also elevates CD20 expression in Pola-refractory tumors (a loss of CD79b?). Hence, these tumors become more sensitive to attacking their CD20. Therefore, the combined application of Pola and RTX should be more efficient than either drug alone for the therapy of R/R DLBCL [454]. Patients with intermediate- or high-risk dnDLBCL who were treated with Pola-R-CHP (n = 440) experienced a lower risk of disease progression, relapse, or death than those who received R-CHOP (n = 439) in the same phase III trial [455]. We expect Pola translation to become the standard of care therapy for treatment-naïve DLBCL. Lonca is composed of a humanized anti-CD19 mAb conjugated through a cathepsin-cleavable linker to a cytotoxic PBD, tesirine. Clinical data obtained on Lonca in an R/R DLBCL phase II LOTIS-2 study have been elaborated on elsewhere [456]. Several clinical trials are ongoing to assess its safety and efficacy in NHL in various clinical settings.

## 16. Bispecific Antibodies and T-Cell Engagers in DLBCL Therapy

The classic mAb consists of two identical heavy chains and two identical light chains linked via interchain disulfide bonds and noncovalent bonds. Functionally, an Ab comprises a tail (Fc region) and two Fab regions containing two antigen-binding sites (two paratopes) that recognize the same epitope. Hence, a classic mAb is bivalent but monospecific [457]. When mAb binds to either complement or FcRs on the surface of cytotoxic innate immune cells (NK cells, macrophages, and neutrophils), the immune signaling cascade commences, leading to target cell death via ADCC, CDC, and/or ADCP.

BsAbs are synthetic Ab-look-alike molecules consisting of two different Ab fragments that each recognize a different antigen or epitope [458,459]. The primary role of BsAbs is to bridge a cytotoxic cell and a tumor cell. IgG-like BsAbs contain an Fc region, whereas non-IgG-like or fragment-based BsAbs lack an Fc region [460,461,462]. The former may have issues with tumor tissue penetration, and AEs due to off-target interactions between Fc and FcγRs can never be excluded [457]. More often than not, BsAbs engage cytotoxic T-cells by co-targeting the CD3 invariant subunit of the TCR complex. The most popular BsAbs are CD19 × CD3ε designs; there are also BsAbs tailored to engage activating receptors, e.g., FcγRIIIa (CD16) on NK cells, macrophages, and neutrophils [457,463]. Bispecific T-cell engagers, or BiTEs, are BsAbs devised to bind, activate, and bring effector T-cells in close vicinity to B-cell antigens and are also present on B-lymphoma cells [463]. The cross-linking of B-cell antigens (e.g., CD19, CD20) and T/NK cell antigens via BiTEs launches a series of signaling events leading to B-cell death via a cellular-dependent cytotoxicity mechanism, similar to how CARTs operate [464]. The activation of T-cells by BiTEs is not MHC-restricted. In addition, BsAbs also promote cytokine secretion via the activation of cytotoxic cells, leading to altered TME. The cytokines trigger the recruitment of a diverse assortment of immune cells, thereby enhancing the immune response to the tumor [463].

Immunomodulatory BsAbs, envisioned as a second wave of tumor immunotherapy, are tailored to targeting tumor antigens and/or immunomodulatory receptors on effector cells so that the immune response in the TME is aroused, causing intensified but selective killing by diverse effector cells while maintaining control over the side effects of systemic immune activation. Another type of BsAb binds two different immunomodulatory targets to achieve overlapping or synergistic anti-tumor effects, such as BsAbs that target two inhibitory checkpoints, one co-stimulatory plus one inhibitory checkpoint, or one immunomodulatory checkpoint + one non-checkpoint target [465]. The innovative constructs are tri-specific antibodies (TriTEs), where at least one of the Abs is scheduled to bind T-cells or NK cells via their surface markers, e.g., CD2/CD3/CD37 [466,467]. The current data and perspectives on developing BsAbs and TriTEs in NHL have been reviewed elsewhere [468].

Two approved BiTEs for treating (D)LBCL are glofitamab and epcoritamab, CD20 × CD3 constructs containing IgG1-based Abs. Glofitamab has a unique 2:1 binding configuration, i.e., two paratopes for CD20 and one for CD3 [469,470]. Epcoritamab binds to a different epitope on CD20 than most other anti-CD20 mAbs [471]. Different BsAbs have been designed and tested in pre-clinical settings and in early clinical trials on R/R NHL patients, including those who underwent CART treatment. The BsAbs therapeutic regimen provides disease control to many patients failing CART therapy, with CR rates from 24% to 35% [468]. The salvage treatment with BsAbs may be preferable to standard chemotherapy for LBCL patients progressing early after CART therapy [472].

Blinatumomab is the first-in-class CD19 × CD3ε BiTE that combines two single-chain Fvs without an Fc domain [468,473]. The drug was effective in an already heavily treated R/R DLBCL population if used with other anti-neoplastic agents and as consolidation for high-risk DLBCL after A(H)SCT [474]. While blinatumomab efficiently generates a T-cell effector response, its half-life is very short, hence continuous intravenous administration is required. The quick clearance of blinatumomab is due to the absence of the Fc portion, resulting in the absence of Fc-mediated effector functions [468]. Blinatumomab may cause significant neurotoxicity, as do other CD19-targeting Abs [475], potentially owing to the presence of CD19 in the blood–brain barrier cells [476].

We mention here several BiTEs currently in clinical development. First, we note (i) odronextamab, a hinge-stabilized fully human IgG4-based CD20 × CD3 construct with low immunogenicity [477]. R/R DLBCL patients who were refractory to CARTs responded to odronextamab [478]. Secondly, (ii) plamotamab, another humanized CD20 × CD3ε construct, but modified for better potency and safety, is being tested in an ongoing clinical trial on R/R DLBCL (NCT02924402) [479]. Lastly, (iii) mosunetuzumab, a fully humanized IgG1 CD20 × CD3 construct, has already been approved for treating R/R FL [480]. Mosunetuzumab is being examined combined with CHOP in a phase II study (NCT03677141) on 40 patients with dnDLBCL [481].

## 17. Adoptive Cell Therapy for DLBCL

The first idea of adoptive cell therapy (ACT) was born in the 1980s when the lysis of otherwise NK-resistant tumor cells was achieved by IL2-activated autologous human peripheral blood mononuclear leukocytes, which were named lymphokine-activated killer (LAK) cells [482]. In 1986, Rosenberg’s team described how they found, in tumor tissues, tumor-specific T-cells, i.e., TILs, and then applied these cells in the clinical treatment of malignancy. TIL therapy encompassed TILs’ isolation from a surgically resected tumor, massive in vitro expansion of TILs, and transfusion back into the tumor. The tumor-killing activity of TILs was 50 times that of LAK cells [483]. With the development of genetic engineering technology, the idea of engineering T-cells ex vivo so they express tumor antigen-specific TCR has appeared [484]. Conventional T-cell recognition of antigens is MHC-restricted, i.e., TCR can only recognize and bind to enzymatically cleaved antigens (antigenic peptides) before these are presented to T-cells from the surface of APCs, where they are complexed with MHC. TCR is an α/β heterodimer with an extension, a multiprotein adduct of CD3ϵ/γ/δ/ζ subunits. In humans, antigen-presenting MHC alleles are broadly classified as HLA class I or class II, which predominantly presents peptides originating from the cytoplasm or extracellular space, respectively [485].

ACT can be briefly summarized into three steps: (i) the collection of immunoreactive cells from peripheral blood mononuclear cells or tumor tissues of patients; (ii) cell amplification in vitro; and (iii) cell transfusion back into patients to directly eliminate tumor cells or stimulate the immune response to damage the tumor [486]. In ACT, lymphocytes are infused into the patient after being genetically engineered or without any changes (as in ASCT). ASCT is employed in the treatment of various types of leukemias, lymphomas, and myeloma. Salvage chemotherapy followed by high-dose consolidation chemotherapy and assisted by ASCT wins the battle against DLBCL in about half of all patients. Hence, it is still the standard of care for fit patients under 70 years [487]. Allogeneic (allo)-HSCT is a remedial treatment modality for DLBCL thanks to the intrinsic graft-vs.-lymphoma effect. R/R DLBCL patients (n = 1268) who underwent allo-HSCT manifested OS and PFS rates of 30.3% and 21.6%, respectively, at 3 years [488]. However, we are witnessing a subsided implementation of allo-HSCT as the new immunotherapies rise. Of note, a small study of a subset of R/R DLBCL patients who failed CART therapy displayed favorable outcomes after allo-HSCT [489].

## 18. Chimeric Antigen Receptor T-Cells in Therapy of B-Cell NHL and DLBCL

Autologous CART (auto-CART) therapy encompasses a procedure in which, first, the patient’s T-cells are retrieved from the blood via leukapheresis; then, the T-cells are activated in vitro and then stably transduced to express the CAR (most often through a lentiviral or retroviral vector); finally, T-cells carrying the CAR (CARTs) are expanded in vitro before being infused back into the patient’s circulation [490].

Like naked Abs, ADCs, and BiTEs, CARTs target CD19, CD20, and CD22 in hematological malignancies. The body has an extensive repertoire of T-cells, each with a unique TCR. The idea of combining an Fv region of an Ab with the constant regions of the TCR to create, on T-cells, a chimeric antigen receptor (CAR) with Ab-type specificity (not MHC restricted) has been on the table for some time [491]. CARs are fusion molecules that possess an extracellular single-chain Fv, derived from a mAb specific for a surface antigen on the tumor cell (e.g., B-lymphoma cell) and then a flexible spacer domain that optimizes T-cell and tumor cell engagement, plus an obligate transmembrane domain and signaling modules that trigger T-cell effector functions [492]. Upon engagement, CARTs form a non-classical immune synapse and then mediate their anti-tumoral actions via the perforin granzyme axis, the Fas/Fas ligand axis, and the release of cytokines, thus sensitizing the TME [493]. Regarding their sensitivity, signaling pathways, killing mechanisms, and performance, CARs share similarities and differences with endogenous TCRs, albeit CARTs’ performance is yet to be entirely understood [494].

The initial CAR constructs contained only one co-stimulatory domain (a signaling module), which, in most cases, was the CD3ζ cytoplasmic domain, rarely a γ-chain of FcRs [495]. However, as it is often the case with drugs of the first generation, CARTs targeting CD20 and carrying CD3ζ (CD20 × CD3ζ) did not display significant anti-tumor activity in clinical trials. The first CARTs also suffered from a low proliferation rate and limited persistence in circulation [492]. CARTs were then tailored to boost the immune response in the TME by intensifying cytokine production. That was accomplished by the fusion of additional co-stimulatory domain(s) with the CD3ζ, such as CD28, 4-1BB/CD13, or OX-40. Thus, the formed “second” and “third” generation CARTs surpassed their earlier versions, displaying higher levels of cytokine production. The novel CARTs exhibited much faster proliferation in vitro than CARTs with CD3ζ alone. All FDA-approved CARTs harbor second-generation CAR constructs [496].

Three CD19-directed CART products have been approved for treating R/R DLBCL. Axicabtagene ciloleucel or Axi-cel comes with a CD28 co-stimulatory domain [497]; CAR in Tisagenlecleucel or Tisa-cel is made of a CD8 hinge region, and a 4-IBB co-stimulatory domain [498]; and the CAR of Lisocabtagene maraleucel or liso-cel is tailored like CAR in Tisa-cel, but possesses an extra non-signaling/truncated EGFR domain that can be used to control unwanted CART expansion in vivo [499]. The data from many clinical studies confirmed that CD19-CARTs can induce prolonged remissions in patients with B-cell lymphomas, often with minimal long-term toxicities, and are curative for a subset of patients [500]. While all three approved products target CD19, their mechanism of action differs, which was confirmed. For example, LBCL patient-derived T-cells (before treatment) and CARTs (post-treatment), explored in Tisa-cel- and Axi-cel-treated cohorts, manifested different hallmarks. Whereas the former demonstrated the expansion of proliferative memory-like CD8+ clones, the axi-cel responders exhibited more heterogeneous immune cell populations. Non-responders to axi-cel had elevated CAR Tregs that were apt at suppressing conventional CART expansion [501].

However, still, a significant proportion of patients only partially respond to CARTs. CARTs raised against other-than-CD19 have been tested in phase I trials, including CD22-, CD20-, CD70-, and CD79B-CARTs. Moreover, strategies using two separate CART products, a single T-cell expressing two CARs, or a single CAR binding two different antigens (tandem CARs) are also under consideration [502]. Then, as the newest concepts, the fourth, fifth, and next generations of CARTs have emerged in recent years [503]. For instance, novel CARTs can be tailored to produce immunocytokine IL12, which can simultaneously engage innate and adaptive immunity. CARTs may be custom-made to impede and reprogram immuno-suppressive TAMs and myeloid-derived suppressor cells in the TME [504], and such CARTs are termed T-cells redirected for universal cytokine killing (TRUCKs).

Numerous studies have explored the usefulness of CAR NK cell therapy in hematological malignancies, where CD5, CD7, CD19, and CD20 were utilized as targets. NK cells, as innate immune cells, offer few benefits over T-cells: there is less incidence of allo-reactivity and NK cells secrete different cytokines, which could help alleviate the cytokine release syndrome (CRS) and neurotoxicity associated with CARTs [505,506]. However, the clinical outcomes were less than expected due to the limited anti-neoplastic effect and low proliferation rate of CAR NK cells (CAR NKs). The research efforts in the context of CAR NKs are moving towards R/R AML and MM [506]. After CAR NK cells, the next goal is to equip macrophages with CARs to attack tumors better. As innate immune cells, macrophages manifest robust phagocytic capacity and skills to present antigens and modify the TME. The MT-101 construct, custom-made to target CD5 on myeloid cells and fight R/R peripheral T-cell lymphomas, is currently being explored in a clinical trial (NCT05138458) [507].

The uncertain success of the CART therapy is one of several issues of concern; the major downsides of CARTs are the considerable cost and complicated and lengthy production process. With that in mind, a prognostic index that would predict the RTT by CARTs is urgently needed. The parameters that would predict the clinical outcome after CART treatment are currently under investigation [508]. In one study, DNA methylation profiles of patient-derived CD19-CARTs affected the efficacy and RTT in B-cell malignancy [509]. Whether distinct lymphoma subtypes may differ in their RTT by CARTs is also a mystery. Interestingly, transformed (=secondary) lymphoma cases experienced a better outcome upon two CARTs-based therapeutics, Tisa-cel or Axi-cel, than dnDLBCL. One study found that the majority of the patients who relapsed (71%) and died (71%) after CART therapy had dnDLBCL [510]. This finding urges more clinical studies before the approval of CARTs as a frontline therapy of DLBCL.

Mechanisms of resistance to CART therapy in patients with DLBCL are likely multifactorial and have yet to be detailed. The authors of the recent reviews have elaborated on identified factors contributing to the failure of CARTs in achieving durable remissions [511,512]. Resistance mechanisms to CART therapy can be primary, i.e., antigen-positive, which happen despite lymphoma B-cells expressing the target antigen on their surface; the reason is likely impaired death receptor signaling and dysfunctional CARTs [513]. On the other hand, there is an antigen-negative relapse because of the loss of CD19 [514]. Finally, other factors may be guilty of refractoriness, such as those employed to produce CARTs, termed secondary resistance, primarily due to poor CART expansion or T-cell exhaustion [513].

Production faces challenges due to the reduced starting number of circulating lymphocytes and poor cellular quality because of prior treatments and the immunosuppressive TME. Add a costly and lengthy production process and it is no wonder the search for alternative products is underway. Among the most attractive options are allogeneic (allo)CARTs [502]. An Allo-CART product named ALLO-501/501A has been tested in a phase I trial where it provided durable responses and a manageable safety profile in an R/R LBCL cohort vs. patients treated with auto-CARTs. The phase 2 trial (ALPHA2, NCT04416984) of ALLO-501A, the first off-the-shelf allo-CART product, is ongoing [515]. Other novelties in the production of CARTs will include selecting special T-cell populations, genome editing by employing CRISPR-Cas9 instead of lentiviral vectors [516], and utilizing cord blood as a source of T-cells [502], to name a few.

An ongoing phase I/II trial in CD19+ B-cell lymphomas is exploring allogeneic, cord blood-derived CD19-specific CAR NKs, co-expressing IL15 (NCT03056339) [517]. Dual CAR NKs that harbor both CAR19 and CAR70 constructs are being tested in R/R B-cell NHL (NCT05842707). Pre-clinical investigations of seven different CD19 CAR-NKs have demonstrated that all constructs enhanced tumor killing and prolonged survival in tumor-bearing mice [518].

Research on CAR-based ACT has reached unprecedented heights, as evident from the number of published research and review articles. One has to use the help of AI with bibliometric and knowledge-map analysis to grasp an immense quantity of scientific pieces. Miao et al. (2022) employed CiteSpace and VOSviewer to retrieve literature on CARTs, published from 2009–2021 [519]. The most influential topics were the utilization of CARTs in hematological malignancies, the issues on CRS, CD19, and the anti-tumor activity and efficacy of CARTs, while among the latest hotspots were, if we exclude studying CARTs in solid cancers, universal CARTs, CAR-NKs, CD22, and anakinra (the IL1 receptor antagonist). The most cited were articles published in Blood, the New England Journal of Medicine, the Journal of Clinical Oncology, Clinical Cancer Research, and the Journal of Immunology [519]. We shall soon witness similar studies dealing with all aspects of basic biological and clinical studies of hematological malignancies. A few have already appeared [520,521]. The clinical story of CARTs is a repetition of the pattern where we catch the novel idea of a molecular construct as a candidate for an anti-tumor drug and create an anti-tumor therapy paradigm, despite lacking a deep understanding of how it works. Then, we put it through pre-clinical and clinical trials hoping for the best and solving the issues that arise on the way.

## 19. Immune Checkpoint Inhibitors as Therapeutic Avenue for B-Cell NHL and DLBCL

In a healthy organism, T-cells ignore endogenous or harmless antigens and will respond, by raising the immune response, only to foreign antigens or antigens derived from pathogens. The molecular signal, such as the appearance of a suspect antigen on the surface of APCs, is scrutinized for a chance to belong to pathogens, and a go/no-go decision is made. After initiating and developing the immune response, the reactions will gradually diminish during the so-called resolution phase. The initial immunogenic event induces negative regulators that lessen the response and are proportional to the stimulus intensity. These molecules, or interactions between two molecules, have been called “immune checkpoints” because they discover and reverse overzealous immune responses. The immune system is tailored to paralyze T-cells in a non-responsive state (“anergy”), and upon an intense signal, triggers the TCR solely, without engaging the co-stimulatory proteins.

PD-1, a cell surface receptor of activated T-cells, B-cells, NK cells, and macrophages, controls the T-cell-mediated immune response by binding to its cognate ligands, PD-1 ligand 1 (PD-L1) and PD-L2. Malignant cells often express PD-L1 and PD-L2, which enable them to interact with T-cells, aiming to dampen T-cell activation and induce T-cell “exhaustion”, which, eventually results in the tumor evading the immune response. Classical Hodgkin lymphoma (cHL) frequently employs a unique genetic mechanism to upregulate PD-L1 expression through copy-gains of the chromosomal region where the genes encoding PD-L1 and PD-L2 reside [522]. The good side of this genetic alteration is that it renders cHL sensitive to the PD-1/PD-L1 blockade. While DLBCL cells do not express PD-L1 regularly, there have been reports of *PD-L1* genetic alterations in approximately 25–31% of DLBCL patients [523]. The therapeutic avenue for Richter’s transformation, an aggressive lymphoma originating from a background of CLL/SLL, still represents an unfulfilled need, and these cases often have a dismal prognosis [524].

PD-1 expression is highly prevalent in Richter’s transformation, accompanied by increased surface density of the PD-L1 protein. Conversely, PD-L1 is seldom seen in other types of DLBCL cells [525]. The exception is the highly enriched PD-L1 protein among non-GCB-DLBCL types heavily populated by clonally restricted T-cells, which often tone down their surface expression of HLA [526]. The frequency of PD-L1-positivity in 1253 DLBCL biopsies was found to be associated with non-GCB type and EBV-positive status [527]. Further evidence that the high level of PD-L1 in dnDLBCL tumors does not predict a favorable outcome came from the analysis of PD-L1+ exosomes from the plasma of DLBCL patients before R-CHOP administration. The levels of PD-L1+ exosomes correlated with indicators of poor clinical outcome, such as intensive Ki-67 expression and double expressor status. Noteworthily, the PD-L1+ exosome content only decreased in patients who experienced CR, making plasma PD-L1+ exosomes a candidate biomarker for the dynamic monitoring of the RTT [528]. Paradoxically, while DLBCL patients with PD-L1 amplification had inferior PFS upon frontline chemo-immunotherapy, in the R/R setting, PD-L1 alterations were associated with a response to PD-1-targeted therapy [526].

Generally speaking, immune checkpoint inhibitors (ICIs), for example, mAbs against PD-1 or PD-L1, or some other ICIs, have shown disappointing results in DLBCL [529]. Monotherapy with nivolumab, a PD-1-targeting mAb, although associated with a favorable safety profile, exhibited a low ORR among DLBCL patients who were ineligible or failed the AHSCT. The disappointing results were attributed to infrequent genetic alterations in *9p24.1* [530]. In contrast, an improved PFS was found in groups of dnDLBCL patients expressing PD-L1 who were administered anti-PD-1 mAb pembrolizumab + R-CHOP, when ORR and CR were 90% and 77%, respectively. At a median follow-up of 25.5 months, the two-year PFS was 83% [531]. The findings above imply that exploring PD-L1 gene and protein alterations in a specific biological subset(s) of DLBCL, and how to target them, may be a goal worth pursuing. However, the addition of ICI therapies to first-line chemo-immunotherapeutic regimens had a better result than ICI monotherapy, and improved RTT could be observed in the context of historical control rates [532,533,534,535]. Still, the long-term OS data are immature, so large randomized trials are warranted. Not a single ICI has been approved for DLBCL treatment to date.

Future exploration of tumor immunobiology and advances in mAb (glyco)engineering will certainly produce novel ICIs that hit other immune checkpoints, such as lymphocyte activation gene 3 (LAG-3 or CD223), expressed on T-cells, Treg, B-cells, NK, and myeloid cells. The surface expression of LAG-3 is upregulated after immune response activation to prevent autoimmunity; the mechanism operates in concert with PD-1. LAG-3 overexpression is characteristic of exhausted CD8+ T-cells. Several other eye-catching immune checkpoints are entering research and discovery in the context of anti-neoplastic therapy: TIM-3, TIGIT, CD47, and B7 family members [536]. LAG-3 binds to the MHC II complex on macrophages, TIM-3 binds to galectin-9 on tumor cells, TIGIT binds to CD155, and CD86 binds to CTLA-4. The biological significance of the TIM3-related pathway in DLBCL was investigated by utilizing RNAseq, IHC, and RT-qPCR data. However, the possible regulatory mechanism of the TIM-3-related pathway in DLBCL was revealed by scRNAseq. In DLBCL tumor tissues, CD8+ TILs expressed TIM-3, especially in the terminally exhausted state. Galectin-9, mainly expressed in M2 macrophages, is the key ligand of TIM-3 and can induce the exhaustion of CD8+ TILs through the TIM-3/Galectin-9 pathway. The enrichment of TIM-3/Galectin-9 is related to an immunosuppressive TME, severe clinical manifestations, inferior prognosis, and poor RTT with CHOP and can predict the clinical efficacy of immune checkpoint blockade therapy in DLBCL [537].

## 20. Exploring CD47 as a Novel Target in Treatment of DLBCL

CD47 might be a game-changer in the therapeutic world of hematological malignancies, including R/R DLBCL [538]. CD47 was first discovered as a transmembrane protein of red blood cells, but it is widely expressed across various normal human cells and many malignant cells [539]. CD47 is overexpressed across lymphoma types and hematological malignancies [540]. Phagocytosis, which is carried out by macrophages and dendritic cells, is regulated by CD47 by sending an “eat me not” signal to the signal regulatory protein alpha (SIRPα) receptor [539]. Phagocytosis proceeds after unmasking prophagocytic “eat me” signals expressed only on tumor cells, not on normal cells, except the senescent red cells [541]. The CD47/SIRPα axis is a critical controller of myeloid cell activation, thus serving as a myeloid-specific immune checkpoint [542]. Compared to normal B-cells derived from peripheral blood or GC, an abundance of CD47 was detected in a large fraction of primary patient samples from multiple B-NHL subtypes. Moreover, the higher the CD47 expression in DLBCL tumors, the worse the clinical prognosis and adverse molecular features [543]. Therefore, inhibition of the CD47/SIRPα complex can enhance malignant cell clearance executed by macrophages. Additionally, the inhibition of CD47/SIRPα interaction augments antigen cross-presentation, leading to T-cell priming and intensifying adaptive anti-tumor immune response. Therefore, inhibiting the CD47/SIRPα axis is impactful on tumor immunotherapy. Studies on anti-CD47 mAbs are a hot research topic, and impressive results have been collected and reviewed elsewhere [539].

## 21. Therapeutic Paradigms Approved for Treating DLBCL

Treatment of DLBCL relies on systemic therapy. Ever since 1997, R-CHOP has been the standard frontline treatment for DLBCL patients, with or without radiation therapy. Of note is that RTX was the first immunotherapeutic used in oncology. Before treatment, newly diagnosed DLBCL patients are classified as those with limited-stage (Ann Arbor stage I or II, without bulky disease or B symptoms) or advanced-stage disease. R-CHOP leads to a long-term cure in approximately 60% of patients [2,544,545,546]. The therapeutic algorithm for LBCL disease management is depicted elsewhere [2]. Approximately 30% of dnDLBCL patients who present with limited-stage disease tend to have low-risk clinical features and favorable outcomes, but the risk of the late relapse cannot be excluded [2]. Furthermore, the risk of the late appearance of second tumor, most likely due to the radiation therapy, is always present, hence opening the venue for clinical trials that skip the radiation and only administer immuno-chemotherapeutics [547,548,549]. Current data underscore the abbreviated course of immuno-chemotherapy for patients with limited disease [545]. Regarding advanced-stage DLBCL, R-CHOP is still the standard of care, albeit being curative for up to 60% of newly diagnosed patients [2,545]. Some elderly patients, including those with poor performance status or other comorbidities, may not be eligible for full-dose R-CHOP. These patients are most likely to be treated with a mini R-CHOP in the frontline setting. In patients with a contraindication to anthracycline, it can be substituted with gemcitabine or etoposide [2,544,545,546,550].

Approximately 10–15% of R-CHOP-treated patients show primary refractory disease, which is an incomplete response or relapse within six months following the first treatment, and an additional 20–25% will have a relapse following an initial response, most often during the first two years [34]. These patients are divided into two groups: transplantation-eligible and transplantation-ineligible. The standard of care treatment strategy for eligible patients has been high-dose chemotherapy (cisplatin or carboplatin-based salvage regimens) followed by ASCT. Because of the treatment intensity, the ASCT approach has only been feasible in half of the patients, and the success of ASCT is limited in patients with primary refractory DLBCL and the early-relapsing, non-GCB subtype, as well as in HGBCL [2,545]. Patients who are ineligible for ASCT due to age, comorbidities, or refractoriness to salvage chemotherapy will be directed toward CART therapy, palliative chemotherapy, or exploring novel agents [550]. Many patients treated in the R/R DLBCL setting will need to switch from treatment to treatment, and most of them will succumb to the disease. The FDA has approved several drugs for R/R DLBCL patients over the past three years that can improve the RTT rate and duration of response while minimizing toxicity. An interesting area of research in the R/R space is how to sequence the available therapies [551]. Of note, the progress in treating R/R DLBCL has far outpaced therapeutic advances in many other hematologic malignancies [552].

CART therapy is reportedly one of the most effective treatments for B-cell malignancies and has been approved for the third-line therapy of LBCL [553,554]. The CARTs approved in the R/R LBCL therapeutic space are axi-cel (Yescarta, Kite), tisa-cel (Kymriah, Novartis), and liso-cel [497,555,556]. These three CARTs had similar efficacy in the treatment of R/R (D)LBCL after at least two prior lines of therapy, and six months after the treatment, approximately 40% of patients were in durable remission, as demonstrated in three pivotal trials: ZUMA-1 for axi-cel [557], JULIET for tisa-cel [498], and TRANSCEND NHL 001 for liso-cel [499]. Many patients have been treated with CARTs, and the FDA alerted the community to the risk of developing T-cell malignancies later after CART therapy [558]. Sporadic secondary T-cell lymphomas at such a tiny rate, therefore, likely had no specific link to CARTs, unless a case is shown to be CAR-positive, i.e., derived from the engineered product itself [464]. Most recent views suggest CARTs should become the standard of care in the second-line treatment of primary refractory or early relapsed LBCL [559,560,561]. CARTs may even evolve into first-line treatment for some subsets of LBCL patients. For example, axi-cel was highly effective as part of first-line therapy for high-risk LBCL (either DHL/THL or an IPI score 3–5 [562]. These recent practice-changing trials of CD19-CARTs in LBCL, including phase III comparisons with second-line standard of care and phase II investigations in transplant-ineligible patients or as part of the first-line treatment, have been reviewed elsewhere [563]. Moving CARTs toward second-line or even frontline therapy has been anticipated ever since the reported change in the TME immune contexture of LBCL tumors after axi-cel treatment. This change suggested that CR and OS were associated with the pre-treatment immune contexture [564], which could be seriously disturbed by previous lines of chemo/radiotherapy. Moreover, the high tumor burden, prominent inflammatory status, and extensive involvement of type 1 cytokines negatively impacted the duration of response to axi-cel [565].

Patients with R/R DLBCL who have failed RTX-based regimens are suspects of resistance to CD20-directed therapies. Hence, therapies hitting alternative targets are desirable in these cases, such as anti-CD19 mAbs, e.g., tafasitamab [410]. Tafasitamab (Monjuvi, MorphoSys/Incyte) obtained accelerated FDA approval in July 2020 [566], but only to be used with lenalidomide in adult patients with R/R DLBCL, NOS, as well as for DLBCLs arising from low-grade lymphoma, not eligible for ASCT [411,567]. Tafasitamab + lenalidomide was the first second-line therapy approved for treating this patient population in the USA [566]. However, as it is often the case, in a recent multicenter real-world study evaluation, patients receiving this combination had higher rates of comorbidities and high-risk disease characteristics, and substantially lower PFS and OS, than the patients from the L-MIND clinical trial [567,568]. Interestingly, neither tafasitamab nor lenalidomide was approved as a single agent for treating DLBCL. Lenalidomide will continue to be used for treating DLBCL patients, albeit more likely in tandem with other novel agents than with chemotherapy [569].

Lonca (Zynlonta, ADC Therapeutics) received accelerated FDA approval in April 2021 for R/R DLBCL after two or more multiagent lines of therapy, according to the results of the LOTIS-2 multicenter phase II trial [450]. The interim assessment of the LOTIS-3 study (Lonca + ibrutinib) in patients with advanced DLBCL demonstrated encouraging anti-tumor activity plus a manageable safety profile, with ORR being 57.1% [570]. Lonca has also been evaluated for the treatment of R/R DLBCL in the following clinical trials: LOTIS-3, Lonca + ibrutinib (NCT03684694); Lonca + RTX (Lonca-R) in parallel with R-GemOx (RTX + gemcitabine + oxaliplatin) in a phase III LOTIS-5 trial (NCT04384484) [571]; and LOTIS-8, Lonca + R-CHOP (NCT04974996), phase Ib for dnDLBCL patients [456]. Pola (Polivy, Genentech) was approved in combination with bendamustine and RTX (Pola-BR) in ASCT-ineligible R/R DLBCL patients. Whilst the Pola-BR cohort had higher rates of AE, the combination resulted in a significantly improved CR rate, PFS, and OS compared to the BR alone [572]. In the POLARIX trial, Pola + chemotherapy was administered to a subgroup of dnLBCL patients, and those who had the ABC subtype experienced better results [573].

May/June 2023 witnessed the approval of two next-generation BiTEs, glofitamab and epcoritamab, for the third- or higher-line therapy of DLBCL or for patients ineligible for CART therapy. Glofitamab-gxbm was approved for R/R DLBCL, NOS, or LBCL arising from FL after ≥two lines of systemic therapy [574]. Epcoritamab (epcoritamab-bysp; Epkinly™; Tepkinly^®^) was co-developed by Genmab and obtained its first (conditional) approval in the USA in May 2023 for treating adults with R/R DLBCL, NOS, including DLBCL arising from indolent lymphoma and HGBCL after ≥two lines of systemic therapy. Epcoritamab monotherapy has also received positive opinion in the EU for treating adult R/R DLBCL patients after ≥two lines of systemic therapy. The clinical development of epcoritamab as monotherapy and in combination with standard-of-care agents for treating mature B-NHLs is ongoing globally [471]. BsAbs generally bring about a smaller extent of immune-related AEs than CARTs and can be administered in community settings [464]. Sadly, though, we are still not convinced that BsAbs can cure DLBCL when used as monotherapy.

Finally, another agent, selinexor, received accelerated approval (in June 2020) for treating R/R DLBCL, NOS, after ≥two lines of systemic therapy. It is not immunotherapeutic but rather a reversible and selective inhibitor of nuclear export, an exportin 1 (XPO1) blocker. The inhibition of XPO1 stops the cell cycle, leading to cell death [575]. Of note, venetoclax has not (yet) been approved for DLBCL, despite showing promising clinical efficacy in a range of NHL subtypes.

Bendamustine, sold under the name Treanda, activates DNA damage through intra- and inter-strand cross-linking of DNA base pairs. It belongs to the group of anti-neoplastic and immunomodulatory agents and its cytotoxic effects primarily result from alkylation-mediated DNA damage and possibly, to a lesser extent, from anti-metabolite properties of its benzimidazole ring. Bendamustine may induce a higher frequency of double-strand breaks in DNA than other alkylating agents, but it is not clearly understood how [576]. Bendamustine was first given as a component of pre-transplant conditioning in the setting of ASCT in R/R lymphomas [577].

In the review presented herein, we referenced several representative clinical trials on DLBCL that were retrieved from the website https://clinicaltrials.gov/ (accessed on 20 October 2024). The website encompasses 1916 active, completed, and terminated clinical trials that recruited adult DLBCL patients of both sexes [578].

Regarding a potentially curable disease like DLBCL, let us cite the others: “The more drugs, the better”. What is missing is direct, high-quality evidence to guide optimal therapeutic sequencing when using different therapeutics in an R/R setting starting from the second line [551].

## 22. Bibliometric and Knowledge-Map Analysis of Scientific Articles on DLBCL

**Figure 5:**
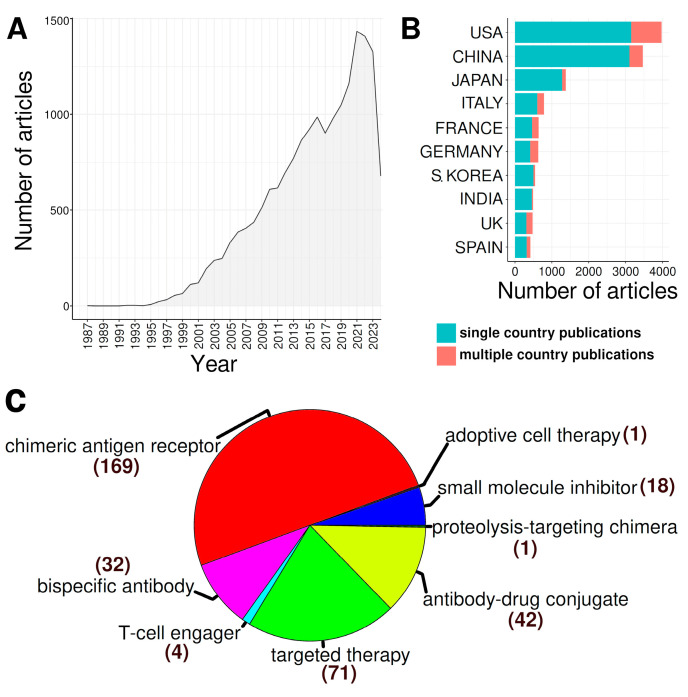
(**A**) The number of publications on DLBCL per year (1987–2023). (**B**) The number of publications on DLBCL per country (2014–2023). (**C**) The number of review articles on various anti-tumor therapeutic modalities relevant to DLBCL, published from January 2013 to June 2024 are shown in brackets. The graphs (**A**–**C**) were created by counting the articles’ abstracts containing the term DLBCL or diffuse large B-cell lymphoma retrieved from the Scopus database. The graphs were plotted in R (v. 4.4.1) using bibliometrix package (v. 4.2.3) for (**A**,**B**).

**Figure 6:**
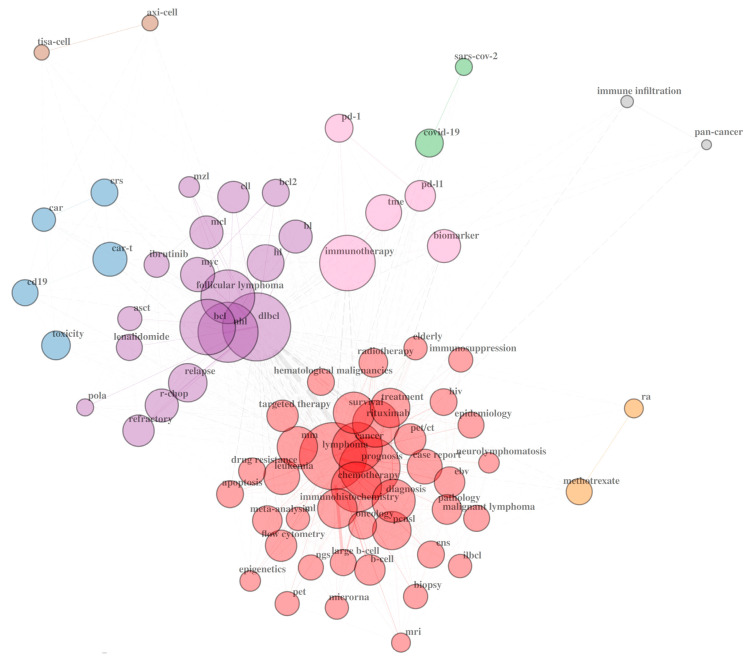
Co-occurrence network of the top 100 most frequently appearing author-chosen keywords in re-view articles on DLBCL published in 2014–2023 and retrieved from Scopus. The graph was plotted in R (v. 4.4.1) using bibliometrix package (v. 4.2.3).

To discover hotspots in the field, we performed a succinct bibliometric and knowledge-map analysis of scientific research articles and reviews pertinent to DLBCL. Such an analysis provides an unbiased look at the cumulative scientific knowledge on this tumor, published from 2014–2023, revealing the most popular and neglected topics. The results of the analysis are displayed in Figure 5 and Figure 6.

Figure 5A shows that the number of scientific articles on DLBCL has steadily grown during the last decade. Moreover, starting from 2017, we witnessed exponential growth of these numbers, which peaked in 2021, followed by a slowing-down tendency in the last three years (Figure 5A). Regarding the affiliations of the authors of articles, USA and China are in the leading positions in the DLBCL research field. There is little cooperation between the countries (Figure 5B), partly due to the issues related to the transportation of DLBCL tissue samples across the oceans. Figure 5C and Table 4 depict the number of review articles published during the last decade pertinent to therapeutic avenues available for treating DLBCL. The most prolific topic was CAR, followed by targeted therapy, ADC, and BsAb (Figure 5C, Table 4).

Figure 6 shows the co-occurrence network of the top 100 keywords extracted from all author-indexed keywords appearing in the review articles on DLBCL published in 2014–2023 and retrieved from Scopus. The three groups of keywords shown in red, green, and blue are produced by the unsupervised clustering algorithm from the bibliometrix R package. The red cluster does not reveal much, whereas the blue cluster encompasses LBCL, CD19, and CARTs, thus reflecting the wealth of literature on this topic. The largest cluster in green comprises the keywords associated with lymphoma types (DLBCL, MM, FL, LBCL, B-cell lymphoma, Burkitt lymphoma, MCL, CLL, HL, and primary CNS lymphoma); general terms (epidemiology, case reports, meta-analysis, elderly, cancer, oncology, malignancy, diagnosis, prognosis, treatment, therapy, survival, relapse, and drug resistance); methodology (PET/CT, NGS, flow cytometry, IHC, and MRI); therapeutic modalities (radiotherapy, targeted therapy, immunotherapy, chemotherapy, R-CHOP, ibrutinib, RTX, lenalidomide, Pola, ASCT, and methotrexate); DLBCL biology (epigenetics, immunosuppression, TME, immune infiltration, apoptosis, PD-1, PD-L1, MYC, BCL2, and miRNA); and, finally, ML and biomarkers (Figure 6). It is evident that the systems biology, deep sequencing, and multi-omics studies on DLBCL that we are awaiting are still scarce since the respective keywords have yet to reach the top 100. Also, PD-1/PD-L1, BCL2, and MYC are heavily covered terms in the articles on DLBCL, but it is high time the community moves toward exploring the roles of other immune checkpoints and cell surface receptors and protein complexes on B-cells, TILs, and TAMs.

The number of review articles pertinent to specific anti-tumor therapies in hematological malignancies, published between 2021 and June 2024, as retrieved from the PubMed database, is shown in Table 4. The hits were reviews whose titles had a combination of two search terms, one from each group. Group I and Group II comprise search terms in rows and columns. Reviews on CARs hold the absolute record with 1218 articles, 510 of which deal with CARs in lymphoma. It is evident that only one review dealt with PROTACs and LBCL (Table 4). Therefore, our study provides an objective view of the trends in a particular research field, accentuating the most sought-after and neglected topics.

## 23. Conclusions and Future Perspectives

The present review is a story of DLBCL, an aggressive B-cell lymphoma curable in approximately 40% of patients, narrated from many different aspects and intended for a broad audience. With an increasing number of elderly people in developed countries, this malignancy shows a tendency of a higher incidence rate. Moreover, viral and bacterial infections present a significant risk for getting DLBCL. Nonetheless, an ample amount of clinical facts and data on DLBCL patients has been collected throughout the world. Still, immense work has yet to be performed.

Regarding genomic analyses, although a sizeable number of tumor biopsy samples have been analyzed and reported, resulting in a large number of publications, there is still room for more of these to be conducted. However, we need the NGS of tumor samples obtained in large cohorts of patients, including males and females of different ages and ethnic groups. The studies should rigorously collect all phenotypical and clinical data, including biochemistry laboratory tests and tests on viral antigens, on these patients so that these can be later correlated with genomic and other omics data. The single-cell transcriptome atlas of tumor samples, such as that in [94], should accompany the abovementioned analyses.

Blood plasma collection from DLBCL patients is also desirable for later lipidomic studies, which would ideally be conducted in well-recognized core facilities in order to obtain reliable data. We are certain that a comprehensive lipidome survey of DLBCL tumors and patients’ plasma, if performed in large enough cohorts, will reveal lipid-based metabolic clusters or putative DLBCL tumor lipotypes. These lipotypes may be able to classify tumor samples independent of genetic subtypes.

Then, the potentially altered expression of crucial proteins in DLBCL, including antigens, cell surface receptors, and metabolic enzymes, should be investigated, preferably employing proteomics studies. Proteomics studies of neoplastic B-cells have seldom been performed [579] but a few more will certainly follow. These studies are important because they lie at the intersection between the scheduled program (transcriptome) and its realization (lipidome of a B-cell). There is a chance that the untargeted proteomic studies will uncover some additional protein marker(s) characteristic of genetic subtypes or lipotypes, such as a proteomic study that discovered two differentially expressed proteins between GCB- and non-GCB-DLBCL [580]. Such a protein marker could become a future therapeutic target.

Many more immunological and biological analyses relevant to pan-cancer biology should be performed on DLBCL tumor and plasma samples. Regarding DLBCL tumor biology, more secrets will be revealed from explorations of the immuno-metabolism and subtyping of immune and stromal cell populations in the TME. Despite several reports, we need more confirmatory studies performed on much larger cohorts of patients. Given the enormous heterogeneity of DLBCL, data obtained using several dozens of phenotyped samples do not suffice for confident inferences. Other avenues that will be expanded encompass RNA modifications (epitranscriptome), the discovery of tumor neo-antigens beyond the exome, glycolipidomic and gangliosidomic analysis of DLBCL tumors in bulk, and isolated neoplastic B-cells.

The big data collected from multi-omics studies must be analyzed and integrated using ML algorithms, preferably deep learning, provided enough data are available. In addition, all data should be deposited in open-access repositories, and in a form amenable to independent data analyses by different bioinformaticians and data mining. Novel searchable databases to accommodate the wealth of omics data that have already been or will soon be produced are in high demand.

Clinical, phenotypic, and genetic data are currently used to predict patients who could benefit from standard R-CHOP therapy. However, alternative therapies for patients with an increased risk of relapse or becoming resistant to R-CHOP are typically proposed based on the clinician’s experience, i.e., based on trial and error; the genetic complexity of aggressive B-cell lymphomas is neither assessed nor considered before therapy. Matching a patient’s genes to drug sensitivity by directly testing live tissues would fit into the “precision medicine” concept [581]. Hopefully, in the future, we will replace the trial-and-error anti-tumor therapeutic paradigm with a rational design of combinatorial therapy, which will employ distinct drugs to target distinct tumor cell populations within the same tumor mass.

## Figures and Tables

**Table 1:** Commonalities, differences, and key features of large B-cell lymphomas (LBCLs).

WHO-HAEM5	WHO-HAEMR4	ICC	Key Pathological and Clinical Features
DLBCL, NOS - Recommended subtyping according to the cell of origin (i.e., GCB- and ABC-DLBCL)	DLBCL, NOS - GCB subtype - ABC subtype	DLBCL, NOS - GCB subtype - ABC subtype	- Diffuse growth pattern of centroblastic, immunoblastic or anaplastic B-cells - Diagnosis of exclusion (panB+) - Hans algorithm for the distinction of GCB/ABC - Large majority of primary nodal origin; do not fit the distinct anatomic site - ABC subtype usually has more aggressive course than GCB
T cell/histiocyte rich LBCL	T-cell/histiocyte rich LBCL	T-cell/histiocyte rich LBCL	- Large malignant CD20+ B-cells (<10%) in a background of predominantly T-cells/histiocytes - Typically affects middle-aged males, advanced stages at diagnosis (lymph nodes, spleen, liver, bone marrow)
DLBCL/high grade BCL with *MYC* and *BCL2* rearrangements	High grade BCL with *MYC* and *BCL2* and/or *BCL6* rearrangements doublehit triple hit	High grade BCL, with *MYC* and *BCL2* and/or *BCL6* rearrangements	- 5–10% of immunohistochemical DLBCL GCB subtype (panB+, CD10+, BCL6+) - *MYC* and *BCL2* amplification without underlying translocation are not sufficient for the diagnosis
		High grade BCL with *MYC* and *BCL6* rearrangements *	- *MYC* and *BCL6* amplification without underlying translocation(s) are not sufficient for diagnosis
High-grade BCL, NOS	High-grade BCL, NOS	High-grade BCL, NOS	Blastoid morphology, more Burkitt-like Genetic findings: LBCL with *MYC* amplification and *BCL2* or *BCL6* rearrangements; LBCL with *MYC* rearrangement and *BCL2* amplification
Primary mediastinal BCL	Primary mediastinal BCL	Primary mediastinal BCL	- Nests of large malignant cells with ample cytoplasm often divided by eosinophilic fibrosis; CD30+, CD23+, p63+, MAL+ - Typically presented in the mediastinum of young women
Mediastinal gray zone lymphoma	BCL, unclassifiable, with features intermediate between DLBCL and classic HL	Mediastinal gray zone lymphoma	- panB variable, CD30+, CD15 and CD45 variable - overlapping features between primary mediastinal BCL and nodular sclerosis HL - Typically arises in mediastinum of young men
Included in HL (nodular lymphocyte predominant HL)	Included in HL (Nodular lymphocyte predominant HL)	Nodular lymphocyte predominant BCL	- Close relationship to T-cell/histiocyte-rich LBCL - Grade1: Fan patterns A, B, and C and Grade 2: Fan patterns D, E, and F
ALK-positive LBCL	ALK-positive LBCL	ALK-positive LBCL	- Plasmablastic differentiation (CD138+, CD38+, MUM1+, panB: may be−) - ALK+, but lack t(2;5) - Extremely rare
LBCL with *IRF4* rearrangement	LBCL with *IRF4* rearrangement *	LBCL with *IRF4* rearrangement	- panB+, BCL6 expression - *IRF4*/*MUM1* translocation - Predominantly children and young adults, good prognosis
High grade BCL with *11q* aberrations	Burkitt-like lymphoma with *11q* aberrations *	High grade BCL with *11q* aberrations *	- Histologically and immuno-phenotypically resembles Burkitt lymphoma, but lacks *MYC* rearrangements, - Aberrations of *11q* usually within complex karyotype
Primary LBCL of immuno-privileged sites: - Primary LBCL of CNS - Primary LBCL of testes - Primary LBCL of vitreoretina	- Primary DLBCL of CNS - Included in primary DLBCL of CNS	- Primary DLBCL of CNS - Included in primary DLBCL of CNS	- PanB+, CD10+ <10% - Recurrent NF-κB pathway mutations, and CDKN2A/B and HLA deletions - brain, spinal cord, leptomeninges/testis/vitreoretina
Primary cutaneous DLBCL, leg type	Primary cutaneous DLBCL, leg type	Primary cutaneous DLBCL, leg type	- Red, raised, rapidly growing tumors on one or both legs, but may occur anywhere on the skin - Typically presented in women in seventh decade of life
Intravascular LBCL	Intravascular LBCL	Intravascular LBCL	- Growth in small to intermediate-sized vessels usually in brain or skin; CD5+ - Hemophagocytic form associated with multi-organ failure
Fluid overload-associated LBCL		HHV8- and EBV-negative primary effusion-based lymphoma *	- HHV8−, not associated with immunodeficiency - Usually ABC subtype, may be CD20− - Presented as serious effusions in elderly patients with fluid overload states (pleura, pericardium, peritoneum)
Primary effusion lymphoma	Primary effusion lymphoma	Primary effusion lymphoma	- HHV8 driven (LANA1/ORF73+), usually EBV+ (EBER+, LMP1 usually−), majority of patients have HIV/AIDS - Immunoblastic or anaplastic, panB− - Presented as serious effusions
Included in lymphoid proliferations and lymphomas associated with immunodeficiency and dysregulation	EBV-positive mucocutaneous ulcer *	EBV-positive mucocutaneous ulcer	- A sharply circumscribed ulcer - presented at cutaneous/mucosal sites (usually in oral cavity) - Often arise on the area of chronic damage, as a result of iatrogenic immunosuppression, and immunosenescence
DLBCL associated with chronic inflammation	DLBCL associated with chronic inflammation	DLBCL associated with chronic inflammation	- EBV+, not associated with immunodeficiency - Large atypical cells panB+, but have plasmocytoid differentiation - Tend to involve body cavities; arise in the area of inflammation (prototypical form is pyothorax-associated)
Fibrin-associated LBCL			- EBV+, arises in the area of chronic fibrin deposition in cysts and pseudocyst cavities (i.e., peri-implant space of breast implants, chronic hematoma, atrial myxoma, endovascular grafts) - indolent clinical course
Lymphomatoid granulomatosis	Lymphomatoid granulomatosis	Lymphomatoid granulomatosis	- EBV+, not associated with immunodeficiency - Angiocentric and angiodestructive - Extranodal sites (lung, CNS, skin, kidney, liver), LN and bone marrow are very rarely involved
		EBV-positive polymorphic B-cell lympho-proliferative disorder, NOS *	- EBV+, with or without known immunodeficiency that cannot be more precisely categorized - altered lymph node architecture and a polymorphic infiltrate that do not fulfill criteria for the diagnosis of lymphoma
Plasmablastic lymphoma	Plasmablastic lymphoma	Plasmablastic lymphoma	- Plasmablastic morphology and immunophenotype; panB usually− - EBV+, EBER+ - Associated with immunodeficiency - Presented usually in digestive tube and oral cavity

Asterisk (*) denotes a provisional entity. Abbreviations: WHO-HAEM, World Health Organization (WHO) classification of hematolymphoid tumors; ICC, International Consensus Classification; NOS, not otherwise specified; LBCL, large B-cell lymphoma; DLBCL, diffuse large B-cell lymphoma; GCB, germinal center B-cell-like; ABC, activated B-cell-like; HL, Hodgkin lymphoma; ALK, anaplastic lymphoma kinase; CNS, central nervous system; HHV, human herpes virus; EBV, Epstein–Barr virus; EBER, EBV-encoded small RNA; HIV/AIDS, human immunodeficiency virus/acquired immunodeficiency syndrome.

**Table 4:** The number of reviews on specific anti-tumor therapies in blood cancers/hematological malignancies, lymphoma, non-Hodgkin lymphoma (NHL), large B-cell lymphoma (LBCL), and diffuse LBCL (DLBCL), published between 2021 and June 2024, as retrieved from the PubMed database.

	Blood Cancer	Hematological Malignancy	Lymphoma	NHL	LBCL	DLBCL
Small molecule inhibitors	2	7	45	7	6	5
Adoptive cell therapy	5	30	10	1	1	1
Chimeric antigen receptors	42	259	510	139	154	114
Bispecific antibodies	4	19	112	35	40	32
Bispecific T-cell engagers	1	11	23	11	8	7
Targeted therapy	14	53	333	52	40	40
Antibody-drug conjugates	6	18	47	22	40	35
PROTACs	2	7	11	2	1	1
Molecular glues	0	1	3	1	0	0

## Data Availability

Data are contained within the article.
